# Supplementary material for: “You see this thing is hard… ey, this thing is painful”: The burden of the provider role and construction of masculinities amongst Black male mineworkers in Marikana, South Africa
Source: PLoS One. 2022 May 23;17(5):e0268227. doi: 10.1371/journal.pone.0268227 (PMC9126392; doi:10.1371/journal.pone.0268227)
Supplement: S1 File — (PDF) [file pone.0268227.s001.pdf]

# SCOPE OF INQUIRY – MEN

**1. Demographic information**

| | |
|---|---|
| Interview code number | |
| Age | |
| Marital status | |
| Educational level (Last grade passed) | |
| Period residing in Marikana | |
| Period working in the mining sector | |
| Which ethnic group do you belong to or identify with? | |

2. Please tell me about yourself, where you grew up and your family.
3. In your view, what are men's roles within families?

   Probe: What are the most important things for a man to do in their families?

   Probe: People often think that women are responsible for tending to domestic needs of the home, like cleaning, cooking, or washing. What happens here in Marikana where the men do not live with their wives/partners?

   Probe: What challenges have you personally experienced in doing things that are expected of you as a man in your family?
4. Please share with me your experience of working in the mines?

   Probe: What does working in the mines mean for you as a man?

   Probe: What is the most important thing to you as a man?

   Probe: What do you have to achieve as a man for you to consider yourself a successful man?

   Probe: How much would you say you have achieved as a man and why?

   Probe: How does being employed in the mines influence how other people view you?

   Probe: How does being married and/or having many sexual partners influence how other people view you?

   Probe: What kind of a man one needs to be in order to fit well among other men in the mines?

   Probe: What kind of men are not popular or considered weak men in the mines?

5. It is known that there was a long strike in Marikana in 2012. As a man who was here during that time, can you tell me more about that strike?

   Probe: Please share with me your own experiences of that strike.

   Probe: What did the 2012 mineworkers' strike mean for you as a man?

   Probe: What did you have to do as a man in order to survive that strike?

   Probe: How important was it for men to be involved in that strike?

   Probe: What particular roles do you think men specifically played in that strike?

   Probe: How important was it for men to be seen as leaders of that strike?

   Probe: Personally, how were you affected by that strike (economically and socially)?

   Probe: What kind of a man was needed to sustain the strike?

   Probe: What kind of a man was seen as useless during the strike?

6. In your view would you say men were changed by that strike, and how?

   Probe: Would you say men had to be more tough, resilient and aggressive for the strike to be successful, and why?

   6.1 There were men who were brave in confronting the police. In that context, were men who were brave and seen confronting the police perceived as leaders of the strike.

   6.2 What does it mean for a man to be brave when facing danger like the men who confronted the police?

   6.3 Was there any fear for you as a man when confronted with police and during the shooting?

   6.4 During the strike, violence tends to be high; and there's violence among mineworkers themselves. How do you make sense of the violence that occurred among mineworkers themselves during the strike?

   6.5 Dissenters (amagundwana) what happened to them or people found to be dissenters during that strike.

   6.6 Dissenters: what do you think made people to beat the dissenters up and humiliate them rather than just stopping them from going to work?

   6.7 What do you think was the message underlying the beating and humiliation of dissenters during the strike?

   6.8 Some dissenters were made to walk naked in public, what do you think was the message being communicated when humiliating the dissenters in this way?

7. Please tell me how men who work in the mines spend time off work.

Probe: What kind of entertainment facilities are available for miners to use for relaxation in this area?

Probe: How do men who work in the mines generally spend their income?

Probe: What kinds of social activities do men in the mines engage in?

Probe: Please tell me about drinking of alcohol among men in the mines (how common is drinking, where do men drink, with whom, how frequently?)
