# Supplementary material for: “You see this thing is hard… ey, this thing is painful”: The burden of the provider role and construction of masculinities amongst Black male mineworkers in Marikana, South Africa
Source: PLoS One. 2022 May 23;17(5):e0268227. doi: 10.1371/journal.pone.0268227 (PMC9126392; doi:10.1371/journal.pone.0268227)
Supplement: S1 Data — (ZIP) [file pone.0268227.s002.zip › Anonymised Transcripts/INTERVIEW 1_anonymised.docx]

**INTERVIEW 711-0159**

***Codes:***

***M= Moderator***

***P=Participant***

**M:** Alright [name]’ I will put it here so that so that we can talk freely. Let us ignore this recorder as we are just talking. Like I said that this recorder is for......as we are sitting here together, whatever we are talking about I won’t lose it you see [name], when I get to my office...**(P..00:25)** so, let us try to speak loud [name]. First tell me, like I said I have some questions I would like to ask you, just answer freely [name] if you do not want to answer you can say no [name] let us skip this one. Be free [name] like I said there’s nothing will happen everything is alright. Your answers, our conversation is for us to understand what happened in Marikana that time, you see [name].

**P:** Yes

**M:** So please, first tell me how old are you, your age, are you married and who do you stay with at home?

**P:** I am married I have children, they are all five… (**01:34**) I broke up with her because we were not married, my first wife passed away, we have three children (twin girls and one other girl) they are all three and they are my children. They were taken care of by the second wife, my first wife passed away and we also buried my second wife. Now, from my second wife I have 2 boys, my old wife I met her when she was young, with the second wife we have two children, [names of children] yes (**02:14)**

**M:** Yes, I get you [name].

**P:** Eh

**M:** So, how long have you been here in Marikana?

**P:** Eh…

**M:** About school [name], if you can tell me, at what age did you dropped out of school?

**P:** Standard 8, I was doing standard 8 and we were calling it standards that time.

**M:** Hmm, I get you very well [name]. Tell me about staying and working in mines, how long have you been working there?

**P:** Eh… I have been working in more than one mine, I worked in Western, it was Western Platinum before (M- eh…okay), I worked in Lonmin, Lonmin is a recent thing (**04:44**) … (M-aha…okay), I worked at Chrome mine (M-okay), in Samancor (M- alright) yes, I worked maybe about 6 here in Mpala.

**M:** Mpala?

**P:** In Gugwini. Yes

**M:** Ohm

**P:** They are three (M-they are three. Oh…), but I didn’t get my money (M: Oh…), no.

**M:** Hmm

**P:** At first, we went there to collect the supporting documents to claim our money that we were supposed to get as a retirement benefit (M: Ehe…), I didn’t get it.

**M:** Oh, you didn’t get it**?**

**P:** Even now.

**M:** Are you still making a follow up?

**P:** To all these mines …. no.

**M:** Huh…. this is bad (P: hmm), if you can tell us [name], are you Mtswane, Mpedi, Mzulu, mXhosa or…?

**P:** No …me…*(coughing)…* my mother is Mtswane, (M: okay…), my father speaks Afrikaans.

**M:** Oh, your father speaks Afrikaans?

**P:** Yes, (M: oh), even me, I took my father’s side (M: uhu…hmm), and my mother ey…I know Sitwane more than Afrikaans.

**M:** Okay.

**P:** But Afrikaans is mine *(saying it proudly).*

**M:** Afrikaans is yours?

**P:** Yes.

**M:** Oh, your father is an Afrikaner?

**P:** Yes, my mother is Mtswane.

M: Okay ke [name]… okay…. alright… (P: Ja), I get you very well [name]…I get you…I get you. When we talk about you, as a grown man and a father, when you look, what are the responsibilities, the things a man should do at home, when you think what are the important things a grown man is supposed to be doing at home?

**P:** At home?

**M:** Yes

**P:** In my house?

**M:** As a man, what are the things you are supposed to be doing?

**P:** Ey… I am supposed to extend this house (M: eh…) and remove these tiles they are not enough (M: hmm) and replace them with the new ones (**07:10…)** (M: hmm), ja… to make it a good-looking house (M: aha) … you see.

**M:** Eh… I hear you, what are other things when you talk about a grown man [name], you see when you say this is a man or a father, what is he supposed to do to show that he is a man in this house?

**P:** He must work, (M: uhu), you see, and respect his job… (M: uhu) eh... and know how to get successful…. without working there’s nothing he can do. (M: when he is not working?) …yes, never without working.

M: Thank you [name].

P (**08:08)** …. just like you, you see these boys. (M: hmm), they are my children, there’s an older one, he is [name] ***(Can’t hear)*** they have children also, the two of them, another is my grandchild. And Adam, he’s daughter is here (……….), I buy them pampers and I am still feeding them because they are not working (M: hmm) …. there’s no job.

M: Hmm…mar…a man who is not working [name], how do they look at him, here in Marikana?

**P:** Ey…you are nothing (M: you are nothing?), no you are nothing, even the woman you are staying with, who looks after you, here in Marikana if you are not working you cannot take care of her, you should at least look for jobs. You see this thing is hard (**09:11**) … so you can say (……...) …*(laughs)…* ey...hay … (M: I get you [name]), ey this thing is painful, (M: hmm) … (**09:40).**

**M:** [name], please let us move and talk, you said you’ve worked in 3 mines, tell me [name] about your experience in working in the mines, how was your experience in working in the mines as a man? Just tell me about your experience, how do you feel about all these years you’ve spent in the mines?

**P:** Ey it was hard that time, ey... (**10:18)** ….and there was no money (M:hmm), hmm … there was no money, the money just arrives now (M:hmm) and the 2010 strike that killed people, that is it (M: uhm), yes, people now are getting money but others died for it (M: others died?), yes they died for money they were demanding (…) , they died for it and they were not fighting they were just sleeping in the mountain and they died there, most of them (M: hmm), that is it.

**M:** So, [name], as you were working in the mines, how important it is for a man to work in the mines, how did it make you in the community as a working man in the area?

**P:** Eh… I saw that there was no other way I didn’t have money, where would I get the money, how was I gonna feed the children. (M: hmm), if I was not working where would they gonna get food whilst sitting down. I got up and go to work.

**M:** As you’ve said that it was not nice to work in the mine, what made you stay and continue working but you saw it was hard and dangerous, you said that (P:eh), what made you to stay and continue working in such a dangerous place?

**P:** I had no money (M: ehh), and that where would I gonna get another job, I was gonna suffer bhuti looking for a job (M: okay), I had to continue until I get this old (M: hmm).

**M:** What made you to leave work?

**P:** I just left because I saw that I no longer have strength to work in the mines, I was underground (M: hmm), I was also working in the construction.

**M:** Do you think [name], working in the mines have prepared you to achieve your goals, the money you’ve earned from the mines helped you to achieve your goals as a man?

**P:** No it was a cent that time, I was not getting enough money (M: okay), per day I was paid R7.00 I worked as an electrician in the mine, I was paid R7.00 per day (M: Jhoo), not per hour but per day . Yes it is.

**M:** So now [name], with such a small amount, did you managed to do the things you wanted to do for your family, your goods as a man as you know that every man has his goals?

**P:** No, it was very small and was not even funny because it was me and my children (M: hmm) (14:21)

**M:** I hear you [name], hmm…so for you to be regarded as a grown man in the community what are the things you are supposed to do for people to respect you?

**P:** Look for yourself (M: hmm…okay), yes you look for yourself.

**M:** I hear you very well [name], I hear you. [name] you’ve said a little bit about the 2012 strike, (P: hmm), tell me [name] about your experience in that strike, what happened there?

**P:** Ey (15:42), the 2012 strike was very painful (M: hmm), those who died were still young and were recently started at work, but they were killed for not going to work, they were staying in the mountain and were not fighting, they wanted the money they were asking. They were staying in the mountain everyday (M: hmm), so the police shot them (16:36) …. Some are still said even now, they left their children and wives, those men were bread feeding their wives and sent their children to school, now they are no more, that is what is painful, and I don’t see the government doing anything about that.

**M:** [name] you saying those people were staying in the mountain, why were they staying there, what were they doing there?

**P:** Hmm…they were just staying, they had their blankets with them, even when it was cold they slept there, they were just staying there (M: hmm), they wanted money.

**M:** So, they wanted money?

**P:** Yes, the money they were killed for (M: hmm), how can you forcefully go to work when you don’t want to, and you want your demand (M: hmm), this is painful and even to me it’s painful.

**M:** Why is it painful [name]?

**P:** Its painful because people from back home are no more in this place (M: hmm) yes, a lot of people I know are gone they shot them and they were not fighting (M: hmm), *clapping hands,* even though they were carrying sticks, but they were not fighting.

**M:** Hmm… about what happened that day, when people were shot [name], tell me where were you that time?

**P:** I was here (M: hmm), eh… where was I suppose to go, this is where I am staying (M:hmm), that time I was retired form work people who worked there are those who were killed ( M: hmm…they were just staying in the mountain?), in the mountain, you see that mountain *(pointing)* (M: yes I can see).. that’s it.

**M:** I hear you [name], tell me [name], you’ve mentioned that they were staying in the mountain because they wanted the money, why were they demanding the money?

**P:** They said it’s a small amount of money, they wanted another percent increase, (M: hmm), yes you see this.

**M:** So, as they were staying there, they wanted an increase that is what you are saying?

**P:** Yes, they wanted a 10 percent, that is what they get today, yes, they wanted the money, they said we are not going if we don’t get this money and they stayed in the mountain they were not going to work (M: hmm), that’s it.

**M:** When did the violence started [name]?

**P:** Eh… the violence started when they refused to go to work but to stay there in the mountain, it was hard for them to get the food even us we were just drinking water (M: you were just drinking water?), yes, what were we supposed to do because water is life (M: uhm), **(21:18**)

**M:** You, [name], for you to survive that strike that day people were shot, how did you survived not to be the one of the people who were shot?

**P:** No, I was not working I was **(21:45)**

**M:** So, [name] there are other things we heard that the time you were in mines, let me put it this way, everybody who was working in mine was supposed to stay in the mountain during that time of the strike, all men?

**P:** Yes, they wanted you if you are working in the mine some were taken out from the shacks, if you don’t go there they forcefully take you there.

**M:** Other workers?

**P:** Yes

**M:** Tell me about this [name], we heard that there were some people, while others were staying in the mountain, there are also those who didn’t go to the mountain who were going to work, where you’ll find that the time others were staying in the mountain, others said no we are going to work.

**P:** Hmm…. Those were killed, were killed by those in the mountain saying that we are fighting for you while you going to work and we said we are not going to work, they were killed for that.

**M:** Tell me about these stories [name], why they were…the stories you’ve heard that time about those who were going to work, what happened to them the stories you remember?

**P:** Let me say, they said they are fighting for them to get the money (M: hmm), but they continue to go to work ***nizenzamagundwana*** and killed them.

**M:** Oh…tell me about Amagundwana, why did they call them Amagundwana?

**P:** (**24:13)**

**M:** So those who killed Amagundwane were also other workers, the people who knew each other before?

**P:** Yes, they were also workers they worked together (M: hmm), others went to the mountain.

**M:** Okay. Then what happened to Amagundwane?

**P:** They killed them (M: Hmm…but…)

**M:** We heard that other Magundwane [name] were killed, so what happened to others because we heard that some were undressed and were naked, what happened, how were they being treated?

**P:** I don’t know they were just killed ,….hmm… eh…when you are wearing a uniform but when you are lucky, you are lucky , it’s your lucky that they just undress you taking off the uniform and go with you to the mountain.

**M:** Oh, so other Magundwane were caught…. how did they identify that you are a Gundwane?

**P:** They said it’s a person we refer as not going there, it’s a Gundwane (**26:15)** … (M: okay), todays things…I dropped from work long time ago (M: hmm), it was bad it was not possible to go around this place… (M: it wasn’t?), no, it was impossible to go, there was a gun shooting only.

**M:** What makes you [name] to think that other workers from the mountain used violence to other workers, what made them to end up killing and slaughtering them, when you think why did they chose to use violence?

**P:** Eh, **(………)** I don’t know how it came up to them to kill others, that I cannot say it exactly what made them to kill others (M: hmm) but it means what they did was clear they stayed in the mountain while some were going to work **(27:49)**

**M:** When you look [name], because it’s been a long time staying here you see, you’ve raised your children here, these people, the other workers who ended up undressing others, were the people who were already violent or here in the community were the people who were bad?

**P:** It is…it is…you can see the first time violent people, people who abuse other people (M: hmm) Because you can never have a thought of killing the person who has done nothing and kill him (M: hmm).

**M:** What forced Amagundwane [name] to continue to ***ukugumba*** while there was a violence that occur?

**P:** Ey its their choice, they chose to work, they did not want to strike and refused to go to the mountain.

**M:** Those people who were staying in the mountain, were perceived as brave or cowered to those who didn’t go to the mountain, how would they perceived you if you are a man and you don’t go to the mountain, would you be regarded as a cowered man or…what was happening?

**P:** They were brave (M: hmm), you were regarded as brave if you go to sleep to the mountain (M: okay), its bravery to sleep in the mountain.

**M:** When you heard [name] about the bravery you talking about, there were those who, when the police carrying the guns coming to them… (P: They went to apologize to them and told them we are not fighting **30:30**), oh, so you saying [name] those people were not fighting they were begging forgiveness?

**P:** Yes, they didn’t want to fight (M: oh), hmm.

**M:** That day the police shot them, were they fighting?

**P:** No… (M: hmm) (**31:38**)

**M:** After they were shot [name], how was the community after people were killed in the mountain…how was it, tell me as the person who was not there?

**P:** It was bad, people were crying…it was bad with cries and starving (M: hmm starving?), starving…hmm... (**32:32)** it was not the nice life… (…..), mothers were crying because they lost their husbands (M:hmm).

**M:** That time [name], people were…as you said they were starving, how do they survived, how people survived?

**P:** There was another car of [name], it was written [name], they provided them food at the stadium because they were starving (M: hmm), It was [name’s] car it was a very big car (M: hmm), others are still getting and other are not (M: hmm.).

**M:** You, [name] as a man that was there that time how did the strike affected you?

**P:** It is still painful even now (M: hmm).

**M:** Tell me [name] how do you feel?

**P:** About the Strike?

**M:** Yes.

**P:** Ey, I don’t like it, it was painful (M: it was painful.), yes very painful, (…….)

**M:** Those people [name] who, as you said it was painful that time, those people who were known that time as Amagundwane here, other were beaten and killed, how are they surviving with people who were on strike now?

**P:** Ey, I don’t know (M: hmm.) I don’t know how they are surviving, really.

**M:** When you heard, how many Gundwane’s were killed?

**P:** Hmm, I don’t know…

**M:** [name] it’s been a while since you staying here, when you remember where were they being caught, the Gundwanes, where were they beaten and killed?

**P:** At work (M: hmm.), they found them in the streets (M: hmm.) … (**36:20-37:14**)

**M:** Were they sympathetic, the Gundwanes?

**P:** No choice… (M: hmm), no choice

**M:** I am thinking that when maybe I was a Gundwane, when they caught me and explain to them that, no I want to work for my children, do you think…(P: even if you say so, they’d say what about us we are the same (M: ehe…) hmm..it is like that, (M: It Is like that [name]), hmm you’d never go to work while they were in the mountain..(**38:08)**

**M:** Do they wait for them on their way back or when they were going to?

**P:** Going to or coming back it’s the same thing.

**M:** They came down from the mountain and fetch them?

**P:** They see you, they were on top there, (M: uhm), they saw that you are wearing a work uniform (m: okay).

**M:** So, what did they do, were they going down from the mountain?

**P:** They asked you that, we are here and where are you going to? And when you saying you are going to work, they will kill you. (M: hmm).

**M:** Th police [name], when you hear about that day, what made them to kill people, you said those people were not fighting so what made them to be shot?

**P:** Eh…. the commissioner of the police, (M: uhm), the one who was called…. it’s a woman… (M: Phiyega) ... [police commissioner] she was the one who said they must shoot (M: oh…hmm).

**M:** What they were saying as you were there when they started to shoot, what did the workers said, how they were doing?

**M:** Eh… (**40:23**).

**M:** Who was the coward [name] (P: hmm) you are speaking about the coward now, what do you mean?

**P:** I mean those who were staying at home were coward (M: oh…), ehm… (M: Those who were staying at home?) yes.

**M:** Those who were staying at home how were they looked by others?

**P:** They went to fetch them (M: oh...), like the way I have said it to you, they went even to the shacks knowing that you are working they go with you, asking why you are staying there (M: uhm…hmm).

**M:** I get you [name], when you don’t want to do what they were saying what would they do to you when you saying you are not going?

**P:** They beat them up (M: oh…) they beat you up and you’ll go, like it or not.

**M:** But why…when I watch, I don’t see the woman who were also working there, been taken to the mountain. Were those women also going to the mountain?

P: hmm…others (M: ohm…okay).

M: oh …I get you [name] (P: eh...), [name] I think we are done with our conversation you see [name]…

**P:** I am thankful also for explaining your questions clearly.

**M:** Eh [name], from the questions I have asked, do you have something you want to explain further, or you want to talk more about, from the things we’ve talked about?

**P:** No, I want to go on now (**43:44**).

**M:** Let me thank you very much [name] and thank you very much for your time, you have helped me, firstly, for allowing me to your house, secondly, you gave your time for us to talk.

**P:** I also like that **(44:12),** I didn’t know you…yherr [name] (M: hmm…laughs…[name]), [name] (M: that’s me [name]) … (**44:39**)

**M:** Thank you very much [name], let me try to talk to others.
