# Supplementary material for: “You see this thing is hard… ey, this thing is painful”: The burden of the provider role and construction of masculinities amongst Black male mineworkers in Marikana, South Africa
Source: PLoS One. 2022 May 23;17(5):e0268227. doi: 10.1371/journal.pone.0268227 (PMC9126392; doi:10.1371/journal.pone.0268227)
Supplement: S1 Data — (ZIP) [file pone.0268227.s002.zip › Anonymised Transcripts/INTERVIEW 2_anonymised.docx]

**INTERVIEW 7110160**

**Codes**

***M = Moderator***

***P= Participant***

**M:** Okay, we can start my brother you see.

**P:** Yes

**M:** So please speak loud so that this recorder catches us

P: This recorder…okay

**M:** Sure, please my brother like I said be free, talk freely, we are two men sitting here we are just talking like I said if you want to talk about something please say and if you don’t want to talk about something say that I don’t want to talk about this, you understand? I am trying to say the information, the question you think you are uncomfortable to talk about Bro…you are free not to talk about, you see Bro.

**P:** Yes

**M:** So, Bro please tell me again, you have already talked to me about this, but tell me, where are you coming from, where are you staying and who are you staying with here in Marikana?

**P:** In Marikana…(M: yes), oh no I am (**name**) I am staying in (**place**) I was born in (**place**) I am staying with another sister but we are not married we are just staying together, she’s from Eastern Cape, so I don’t have parents I am staying with her so she is the one who looks after me because I was sick in 2013 (M: hmm), yes I retired from work by medical board (M: okay), eh…after the strike you see the 2012 strike (M: hmm), it started when I was still working (M: hmmm) it took six months (M: hmm), so after six months it was January 2013 I got sick you see (M: hmm) I was suffering from TB I found that the mine is releasing me, I was given a medical board (M: hmm) so even now I am not working eish I am hardly surviving, I am supported by my brother , this strike I can say it didn’t affect me like the 1985 strike, I was still working I was in **(….)** (M: hmm), eh…there is another mine that is called Eastern (M: okay), yes I was there and that time it was during the apartheid time , the time [prime minister] was governing (prime minister), yes …so [prime minister] abused us very much (M: uhm), we were then arrested that time in [name], there is a police station called [place] (M:hmm), ey they had beaten us up very hard, those police of [place]….so 2012 strike, no it didn’t affect me, you see, (M: hmm), but this one of 2012, ey many of our brothers were affected and some of them died (**02:51**) (M: hmm)…hmm.

**M:** Let us talk about these strikes my brother, this one of 1985, what happened?

**P:** This one of 1985, (M: hmm) we were striking for money the manager that time was [name], there was a manager wo was there, [name], so we were striking for money so he refused to give us the money (M: uhm) so we vandalised/destroyed a lot in the mine you see (M: hmm) so I was arrested we were sent to [place] all of us who were arrested ey, the [place] police were trampling on us (03:36) we spent 3 months full there in 1985.

**M:** In jail?

**P:** Yes, it started in July and I got there on the 1^st^ of July.

**M:** Okay

**P:** Yes, we were asking for money (M: oh) yes, no this 2012 one I wasn’t there.

**M:** You were not in 2012 strike?

P: I was not there in 2012 strike, during that strike I was sick (M: okay) but the way I heard because I am staying here, I heard from those who were there about what was happening.

**M:** Tell me Bhuti about anything that you know about the strike of 2012, everything you know where did it started what was happening and what ended up happening?

**P:** The strike of 2012, (M:hmm) they were fighting for increase you see (M: yes) then they found that the manger was refusing with an increase (M: hmm) (**04:48**)..then on the 16^th^ of August the strike started (M: okay) until it ended on the…….I think…it took 6 months, September, October, November, December, January, it was February yes (Mhmm), around March it was the end of that strike, so others got back to work and others were dead, eish that strike hurt a lot of people (Mhmm), so I was not affected because I was in (**name of the hospital**) (M:hmm) I was in hospital that time.

**M:** when you say it hurt a lot of people Bro(..) what do you mean when you are saying so?

**P:** When I say that (M: hmm), ey they shot a lot of people some were hurt by the police because they were fighting back (M: hmm) yes.

**M:** Those who were shot, why they got shot Bro (**name**)?

**P:** Ey, they were demanding money (M: hmm), about 2012 strike, I cannot tell about it I was not there I was in hospital I heard from people who were there on that strike.

**M:** Okay, when you heard, like when we were talking with other brothers Bro (…), they said those men, the workers were staying in the mountain (P: in the mountain?) yes…tell me what they were doing there, why they were staying there and what happened when they ended up got shot?

**P:** Ey, about shooting I don’t know Bhuti (M: hmm) I would be lying because I only know that strike was for people who wanted money (M: hmm), about the shooting with the police it’s what I don’t know (M: hmm) I just saw it from the news.

M: Tell me I heard that, they were called as Amagundwana why were they been called as Amagundwana?

**P:** Eh…I only know one who was killed in the mountain, it was the Gundwana, there was a meeting there, yet he was busy recording who was talking, and they saw that he was recording (M: hmm), they killed him in the mountain. Majority of them died because they were spies you see, when they talk about something they recorded it and mix it (M: hmm).

**M:** This, this…. oh, so this thing of them being called as Amagundwana where does it come and what does it mean?

**P:** It means they were spies (M: oh) yes, they were buying the whites and were told to check who is in the front and so on …Most of them were arrested here (M: arrested?), yes in jail others got hurt and went to hospital and others were shot dead.

**M:** Did they got shot because of the Gundwanes or?

**P:** That time they were shot by the police (M: hmm…okay), yes that is the little I know I don’t know it very well.

**M:** You don’t know it very well?

**P:** Yes.

**M:** So, those people who were Gundwanes Bro(..) where were they staying, were they also workers, who were they and what happened to them?

P: They were also workers (M: oh…) yes, they were staying separately (M: okay) here in the mine people are not coming from the same place (M: okay) but most of the people who died are from the Eastern Cape.

M: Why is it them mostly, what did they do?

P: They were the ones who were in the forefront of the struggle.

M: Let us talk about these people who were in forefront of the strike Bro (name), maybe from this strike and that of 1985, who are the people who lead the struggle, what makes you to be in the forefront of the struggle?

**P:** Let me say that you are the speaker of the workers, you’ll find that they demand maybe 10% from the manager and the manager refuses to give and offers to give a certain amount and then they tell people to strike and say we are not going to work (M:hmm), eh…so Amagundwana are those who are the spies (M: hmm), they listen to what they say and send it to the management….yes

**M:** So Bro(..) like you said that in 1985 you were there, you were arrested by the police you were not in this 2012 strike, those who were in the forefront of the strike and those who ended up staying in the mountain, are the men who know themselves that they are brave, I am trying to understand who was suppose to be in the forefront of the strike or those who are cowards are left behind, how does it all go?

**P:** People who go there are those who are brave they are the ones who go there (M: hmm), eh…that one of 2012 I don’t know it very well because I wasn’t there I was in hospital (M: yes).

**M:** From the 1985 strike, for you to ended up being arrested you were also in the forefront?

**P:** No we were striking we got arrested as many of us, you see (M: yes) I was involved in breaking of the bars and the kitchen we were burning things like that you see (M: uhm) then we saw that the police are coming shooting the teargas.

**M:** What made you Bro(..) in that time to decide in the burning of things what made you to decide about that?

**P:** We wanted money so… (**11:42)**

**M:** I am trying to understand, I get you Bro (…) in your broader scale that you were demanding money but what made you to end up using violence, burning and breaking things, what made you to change the way of doing things? At first you told them that you want money (P: yes) you ended up doing another strategy of burning things, breaking and etc, what led to that?

**P:** The police were shooting us they were shooting the workers (M: hmm) even the one of 1985 people died but they were not the same as the 2012 strike (M: hmm) it never took a long time.

**M:** Hmm…so even from the 1985 strike there was also a confrontation between the police and the workers?

**P:** No, but the workers did not go to work while the police was forcing them to go to work.

**M:** Hmm, so when did they shot them what happened to end up shooting them?

**P:** They were gathering in a meeting and then they separated them by shooting teargas and they refused to move (**13:37),** so I don’t know because I was not there during 2012 strike I was in hospital (M: yes Bro…okay), yes.

**M:** Let us move [name] and talk about you as a man, you see.

**P:** Yes

**M:** Tell me about your experience in working in the mines [name] you’ve worked in the mines for a while tell me about your experience how is it to work in the mine?

**P:** Ey it’s hard to work in the mines.

M: Hmm, why are you saying that just tell me and be free, explain it to me, my problem is that I never experienced it and I have never saw it, just tell me and tell someone who doesn’t know [name] why are you saying it’s hard?

**P:** Ey, it’s hard because I was a general worker you see (M: hmm), eh I was not like the (**14:06)** and our foreman were trampling on us at work, you see (M: hmm) eh, (**14:15**).

**M:** What other things that make it hard there besides trampling by the foreman?

P: No there are no other things without this…no there are no other things.

**M:** But tell me [name] like you saying it was very hard to work in the mines but what made you to continue working there while you know it’s not nice?

P: Ey it was hard because I was supporting my siblings, my two brothers and I don’t have parents, my mother passed away in 1983 (M: okay) eh… so I had to drop out of school and go to work so that I can support my brothers and push them to go to school that is what made me to continue working, now if I wasn’t retired because of sickness I’d still be working now (M:hmm), so I was retired for medical reasons and I was underground I was affected by those chemicals (M: yes), so I got medical but before I was given a medical the doctor told me I am given a surface job you see, I got out of mine to work in the surface, so there were people who were called as HR ,you see (M: yes), Human Resource, I suffered a long time trying to tell them and I had the supporting documents from the doctor which states that I’ll not be able to work underground, the doctor said I must be given this(…..) they said there’s no space (M: jho), hmm then I got a medical retirement (M:hmm), eh.. now I have 6 years without working.

**M:** is it possible to go back?

**P:** No, long ago, jobs were sold (M: hmm), those HR staff are criminals…hmm

**M:** You told me [name] that after your mother left, you had to work (P: hmm) what made you to think that it is your responsibility that now you have to work?

**P:** Eh…because we were suffering from back home (M: hmm) so I had two brothers who are coming after me they were at school and I had to leave school and go to work (M: oh), hmm.

**M:** Okay I want to talk to you [name] as a man, a man at home what is he supposed to do to show that he is a man or in the community?

**P:** When you are a man you are supposed to get married, build your own house and have your own family.

M: If you have not done that how do the community perceived you?

**P:** Ey, you are not being recognised.

M: Tell me, what do you mean when you say you are not recognised?

P: How will I say it, because you will never get a wife and you will never have a family (M: hmm) you must show that a man should work.

M: Bro [name] for a man like you for an example and other men who are in the same situation, that you are not working, now how is your status here in the community when you are a man who is not working and who doesn’t have money?

**P:** Ey my status is very bad I survive by making piece jobs you see (M: hmm), so that we eat before we sleep because even my brother, he is working (M:hmm) sometimes at the end of the month I go to him and ask for money because the woman I am staying with is also doing piece jobs (M: hmm) eh..she’s cleaning for other people and making their laundries (M: okay) so that we have something.

M: As you are saying its hard for a man who is not working, how people look at you, do they look you good or bad, are they saying you are not a man, I am trying to understand such things, that if you are not a working man how people look at you?

P: You know they do not consider you as a person they undermine you (M: hmm) …hmm.

M: How do you feel about this Bro [name]?

P: It makes me feel painful I do want to work but eish I always get piece jobs, you know piece job or contract, they end but I struggle to get permanent job (M: hmm) but I still try to find the piece job (**20:15)**

M: If Bro(..) you’ve tried but no luck, how does this make you feel as a man who can’t bring something at home?

P: Ey I feel bad it makes me feel painful.

M: Explain to me Bro [name] how does it feel when you can’t try, you see?

P: My brother helps me, (M: hmm) he provides me with something I need every month, then I wake up and try to find a job, Bhuti, when you are not working no one sees you like a person (M: hmm) …hmm.

**M:** You Bro [name], what are your wishes or goals as a man, for you to be proud of yourself what do you want to achieve?

**P:** My wish is, I want to get married, build my own house because where I am staying it’s my home you see (M: hmm) this one is for my mom not mine, I want to build mine because another brother of mine passed away ,the last born (M:hmm) then there’s another one but he’s not here he’s with my aunt in [town] you see.

**M:** The other brother you talked about?

**P:** My brother the one I was talking about is working he has his own house he is staying in (……) (M: oh…here?) yes here in North West (**name of the place**) is this side (pointing), when you are going towards the mine, the Eastern Mine, yes, he is there in Eastern, (**from 22:21**).

**M:** Is he working there?

**P:** Yes, he is the one who is supporting me.

**M:** Oh…so he is the eldest?

**P:** Yes, he is older to me.

**M:** Hmm, alright Bro [name], so you told me about your goals you want to achieve in life. In this situation you are facing now, how far do you think you can achieve these goals?

**P:** You know this will happen because even now I have hope, there are other people from (place***)*** they come to check every after 3 months, they come to check us all those who retired for medical reasons, they asked us how do we feel, and I always say one thing that I want to go back to work you see (M: okay), because the doctor said I must not work underground I am suppose to work in the surface (M: hmm), so they said they will also try (M: alright) because after 3 months they come with soap, dawn…cosmetics.

**M:** Where these people come from, are they from the mine?

**P:** They are form [place] offices (M: oh) from [place] offices (M: oh) they check all the people who retired for medical reasons (M: okay) but they come after 3 months, you see even now, last month they were here (M: okay Bro) there is something called **isityo**, (M: okay), you mix it with water (M: hmm), when you have money you can buy milk and mix with it but they do not come for that they come to give us cosmetics (M: oh…all those,) all those who retired for medical reasons they check how are we doing.

M: Hmm, I get you very well my brother (P: eh), from here in the house, being with no job/without work while staying with a woman, how does that make things in the house?

**P:** Ey it does not make it any better.

**M:** Tell me more Bro [name].

**P:** Ey I don’t feel well you see because the person I am staying with at least she is trying you see (M: hmm), she finds the piece jobs even now she is not here she went to try find something out, then on month end I go to check from my brother or sometimes he comes to check what do we eat (M: hmm) I still try to find job (M: hmm) and I don’t feel good about being without job.

**M:** Does this affect your relationship with the sister you are staying with, this thing, because you are not working?

**P:** No, it doesn’t (M: hmm) we understand each other because during December she usually go home (M: okay) hmm…yes, she is living in Eastern Cape.

**M:** Hmm and then she comes back?

**P:** Yes, she comes back (M: oh…I get you very well), because we met each other here she came here to look for a job (M: hmm) yes.

**M:** Oh, like you said that sometimes it is her who brings food, how does it affect your relationship, how does it makes you feel as a man in the house or her as a woman?

**P:** No, it does not make her alright because I stay longer without working and it also doesn’t make me feel good (M: hmm).

**M:** What makes you to feel bad my brother?

**P:** Because there’s no permanent job, you see (M: hmm) and because my brother sometimes ey he’d tell me I don’t have money so and so, he is the one who pays the life insurance (M: hmm) he also has his house, you see (M: hmm) and children?

**M:** Hmm, I get you my brother very well, let us go back a little bit, you’ve said that the people from the community they do not look you good if you are a man who is not working, how do they show that they undermine you my brother?

**P:** Ey, you know people even if they do not show you, but you can see (M: hmm), hmm.

M: I get you very well Bro [name], eh…thank you Bro [name].

**P:** Okay.

**M:** Thank you very much for your time.

**P:** Yes

**M:** Thank you very much for the time you’ve given me, to sit with me here and discuss, like the way we discussed (P: eh), this is the first time you see me Bro [name] but you are free and you have given the respect, thank you very much about that. I don’t know about the things we have discussed if you have anything you like to repeat, or you want to discuss it furthermore or?

**P:** No, well, I am covered about them, the only thing I can say, I don’t know if you can try to find me a job (M: oh), hmm.

M: I hear you my brother (P: yes) hmm…no Bro [name] we are hoping as you said, we are people who are doing research for you but we are hoping things will be better Bro [name] and I am also motivated about what you are saying, at least there are people from [place] who come to check you, because if you retired for medical reasons it would be better to go back to work, you see, you are still young.

P: Yes, this is what I am wishing for

M: Hmm, to go back and work…no let’s hope Bro [name] it will be alright (P: hmm), let’s hope (P: okay). Thank you very much my brother
