# Supplementary material for: “You see this thing is hard… ey, this thing is painful”: The burden of the provider role and construction of masculinities amongst Black male mineworkers in Marikana, South Africa
Source: PLoS One. 2022 May 23;17(5):e0268227. doi: 10.1371/journal.pone.0268227 (PMC9126392; doi:10.1371/journal.pone.0268227)
Supplement: S1 Data — (ZIP) [file pone.0268227.s002.zip › Anonymised Transcripts/INTERVIEW 5_anonymised.docx]

**INTERVIEW: 711_0147**

***CODES:***

***M: MODERATOR, P: PARTICIPANT***

M: Alright Sir, thank you very much for allowing me this time to talk to you. As I mentioned before, we are doing a research, we trying to find out about the lives of men working in the mines. I will not publish your identity. After transcribing this, we will delete the record. So don’t worry about your identity being revealed.

P: Alright.

M: Can I start by asking your age please?

P: [Year].

M: You were born in [year]

P: Yes.

M: And where is your home.

P: I’m from [place].

M: Are you married?

P: Traditionally.

M: Oh traditionally?

P: Yes.

M: And what your last grade at school?

P: I ended at a very low standard. Standard 7.

M: Oh you passed standard 6?

P: Yes. I had to leave school because of circumstances.

M: How long have you been staying here in Marikana?

P: From 2007.

M: Ok. And how long have you been working in any mine?

P: This is the first one and I started here in 2007.

M: Which ethnic group are you from?

P: I’m a Xhosa.

M: Ok. I will now ask you to please be open with me and tell me where you grew up.

P: I grew up in [place] and only left when I was coming to work here. I didn’t stay anywhere else.

M: Ok. Please tell me about your home and your family? How many are you in the family and etc.

P: I’m not coming from a rich family. They all depending on me, I’m the bread winner at home. My father passed away. My mother is alive and is a pensioner now. My sisters are depending on me.

M: How does that make you feel?

P: It’s painful to know that you are the only one working and the whole family depends on me. And the money we get here doesn’t even get to R10 000.00. Only when we work over time, then we get a little more than that. I rent here and the kids are at school and I must send money for food and clothes.

M: I hear you say it’s painful. As a young man how do you feel having such responsibility at this age?

P: I’m the man everyone is looking up to and depending on, so even if I complain I can’t complain much because there is no one else to take these responsibilities.

M: I know your family is back home, but I want to know if you have people that you go to when you have a problem here?

P: It’s these men here. We help each other. When I need something I go to them and they help me. Even if I need money I borrow from them and pay them when I get my salary. I don’t need to go to loan shacks but I have people with money here.

M: So who do you stay with here?

P: I stay alone and there is no mistress, no.

M: *(laughing)* No mistress?

P: No.

M: Alright I hear you. In your own view, what is a real man’s responsibility?

P: It’s providing.

M: Please be broad.

P: I mean a man’s responsibility is taking care of the wife and his children. Clothe them and feed them. The kids must not go hungry, or wear worn out clothes.

M: How do you view a man that doesn’t do those things?

P: We view him as a stupid man. There’s a lot of men here that are like that. They finish their money here and don’t send anything home. We see their wives coming here to collect the money. That is even more common on us Xhosa people.

M: How do these men spend their money if you say they don’t send it home?

P: They go drink and spend it here on the shacks. On the 10^th^ of the month the money is already finish. And this person didn’t send a cent home though he knows he has a family back home.

M: Other men that know this man is doing this, how do they view him?

P: They seat down with him and remind him what he is here to do and is not supposed to spend money like that. They tell him ‘man we are sent here, we are not here to play, don’t forget.’

M: (laughing) I like the way you put it.. ’We are not here to play’ can you please explain more there.

P: We are not here to work for ourselves, we working for our families.

M: So some men are sent here but they get here and play?

P: Yes.

M: How easy is it for men here in Marikana to do what expected of him?

P: It depends on knowing what you are here to do and do just that instead of finishing money here.

M: I hear you Sir.

P: Yes.

M: Now as you mentioned that you are the bread winner at home and the family depends on you only. How difficult is that sometimes?

P: It’s very difficult. Something happened sometime back. My sister’s child passed away and I had to burry him. I borrowed money from [place] and buried him. He was almost 10 years old. His father didn’t care about the child. I am not complaining about that. And also other things happen, but all these things just make you stronger and grow.

M: Oh! I’m sorry to hear that Sir.

P: Yah.

M: If you didn’t manage to burry your sibling how were you going to feel?

P: I was going to hurt very much, very, very much because who was going to burry him except me.

M: When you just looking Sir, men form here, how do they use their money, on what do they use it when they get paid?

P: Some send it home and some play with it here in the shacks. But we are different as people.

M: Please explain to me, what do you mean when you say they play with it in the shacks?

P: They get paid and go drink in the tavern, that’s playing with money. You drink from morning to noon finishing money as if you were just given this money, but you worked hard underground for it.

M: What do you think make people misuse money like that?

P: I can say it’s stress maybe on other people.

M: When is this stress coming from?

P: It depends. These stresses are different. But the stress the other guy had was caused by the argument between him and his wife. He said the wife took money to her home. But I didn’t understand why he would stress because of that because the wife can’t let her mother starve but there is money in the house. That shouldn’t have been the cause for him to go drink the whole night at the tavern. That was not the solution. He should have just sat down with his wife and that problem, although it was not a problem the way I see it. When you are working you are not working for yourself only, you working for others as well.

M: Alright, if we move away a little from the money issue. Men from around here what do they do when they are off work, like on weekends?

P: They go drink beer. That is how they spend their off days. They don’t go to the soccer field or rugby field where they go play and have fun. They just drink. But problems differ. I can’t work and go drink all the money. I also drink but only occasionally.

M: Why are so careful about the way you use money?

P: It’s because I work very hard for it.

M: You have mentioned more than twice that you work very hard here. I have never worked in the mines, could you please explain to me the working hard part.

P: We work underground, and we use a lift to go down there. That lift get stuck halfway sometimes, especially now winter is about to begin. It’s like from here to [place] walking underground, that’s working hard.

M: The way you describe it, it sounds like it’s dangerous.

P: It’s dangerous because it gets stuck when it’s cold.

M: I hear you Sir.

P: Yes.

M: When you have a problem with your wife how do you solve it?

P: I seat down with my wife and discuss the issue. But I don’t know if it’s just my luck or what because I don’t have much problems in my marriage. I have seen other men having marital problems and some even get married three times.

M: What do you think is causing those problems to be minimal in your marriage?

P: I don’t know. Maybe it’s because she still listens to me. We have been married since [year] but she still listens to me. Women have a problem of copying from others and staying there is a problem in bed, but it would just be a way to tell you that they have met another man. But I don’t have those problems in my home.

M: Alright I hear you Sir. Let’s now move away from your marriage and discuss women in general. I know you don’t have a mistress, but conflicts happen even in the community. So when those problems arise how do you as the community solve them, especially here in [place]?

P: There was an office here where they used to solve issues like that, but now they moved because the lady that was working here was retrenched.

M: Oh shame. Now what do you do when you have problems here in the yard?

P: Here in the yard we seat down and discuss the problem with the landlady.

M: Oh ok. Now Sir like I said, some of my questions touch on the 2012 strike. You were here when it was happening. Can you tell me more about that strike.

P: Eish! That strike was a huge blow because it affected us a lot. We were not fighting as workers, we were just fighting for our rights. We wanted more money, but we were told about percentages and we are not educated we don’t understand the percentage talk, we understand one hundred rands not percentage.

M: *(laughing)* Ok.

P: That strike really made us struggle and go hungry. I’m worried even now because they saying there might be another one in July. I don’t know what’s going to happen.

M: You are saying to me this strike made you go hungry…

P: Yes Sir, especially on debts. We have debts and people had cars but those cars were taken.

M: So if the strike caused all that what made you to continue with it for so long?

P: You know when you want something, you endure even if it’s difficult, just to get what you want.

M: I hear you. Please tell me more about that day of the strike, where were you and what was really happening?

P: I was lucky that day because I was seating up there on the mountain, maybe I was saved by my ancestors. While seating there my wife called and she was pregnant at the time.

M: With this small one?

P: Yes with the small one. So she called me because she was leaving to go to her sister’s place in [place] that day. I went to help her prepare and arrange transport to take her to [place] taxi rank. After she left I just heard people screaming. When I looked I saw it’s the police vans and others are on horses, not police, soldiers on horses. The shooting started and it was really ugly. And we treated badly, sometimes you’ll be sleeping at night and you just hear the door being kicked and it’s the police. We started fearing for our lives.

M: So as a man what did you do in order to survive?

P: I ran, nothing else, I ran.

M: Ok.

P: And I was saved by that call from my wife otherwise I would have been amongst those people that were shot. Maybe I would have been dead or have a limping leg now. But God protected me.

M: We saw on TV that men had weapons and they were using them. Do you think that strike changed people?

P: People learned that strike is not good. Yes it helps but lives are lost when the police comer. And some police are our home boys, they sent from Mthatha. And they send soldiers as well, and it’s those strong and powerful soldiers that are sometimes sent to America.

M: Ok. What I mean is before the strike men were not violent maybe, but after the strike do you think they changed and became violent?

P: Yes that happened. During the strike men organized a witch doctor to prepare something to make the men bullet proof and they say that thing works. So they ate the aloe but it was eaten by the men that were in the front row. I would have also eaten it but I was at the back.

M: Ok.

P: And that thing didn’t want cowards, you must not turn your back because you will put others in danger.

M: The ones that didn’t eat?

P: The ones that ate. You will make them weak.

M: As you are talking about men that are cowards, tell me about the men that were seating there although they saw it was dangerous, what made them seat there?

P: We were seating there because we were not fighting with anyone and we knew if we seat by the stadium the owner will complain. But by the mountain, no-one will complain because it’s a place for goats and cattle.

M: Ok I hear you Sir.

P: And another guy from there is still disturbed mentally since that day. I think the strike affected him badly.

M: Yho shame. As you are mentioning this guy that got affected like that by the strike, how much do you think the strike affected other men emotionally?

P: I won’t talk about other men, I will talk about myself. I didn’t even eat that day because my heart was very painful. Seeing something I didn’t expect. Gunshot noises kept ringing in my ears. I can’t talk about other people because I don’t know how they felt.

M: Ok. How long did that effect remain?

P: About two weeks.

M: But you are fine now?

P: I’m alright now.

M: You are alright?

P: Yes. I used to see that on TV, I never thought I will ever see it in front of my eyes.

M: I hear you Sir. So how did that experience make you feel about working in the mine?

P: I felt like leaving the job, but then again I remembered that I have a newborn baby that I need to financially take care of and I can’t just abandon that responsibility.

M: I hear you. So what kind of men do you think were needed in order to survive that strike for that long?

P: Men that trust themselves. You know it’s very sad when you get you salary and it’s finished the same day. You can’t even buy things cash, you always paying installments.

M: I hear you Sir. Thank you for sharing your experiences of that day. Now let’s move to you, since you arrived here, how do you see this place, working here as a man?

P: Like in North West, [place] or working under ground?

M: Working in the mine generally how is your experience?

P: I wish my child never have to work here. It’s not right here, it’s too difficult working here.

M: What do you mean?

P: There’s machines that are very heavy and we use them every day. When you off work you can feel that your body is very sore because you are always using these heavy machines. The mine creates old men.

M: When you say the mine creates old men, what do you mean?

P: I mean men come here young but the hard work here makes them age quickly. Those are the things we complain about. You work very hard but the money is little. And on the other side you get a call from home saying your child is sick. You need to arrange money now for the doctor.

M: How do you feel as a man when you fail to provide that money?

*(Background noise, a woman shouting at the child)*

P: You feel very sad. It’s not a good thing to fail. You tell them you will send money next week but the child is sick now.

M: How do you feel then?

P: I don’t feel alright failing like that. And borrowing money is not a good thing.

M: I hear you Sir. Another different question now. What is important to you as a man?

P: I would love to send my children to tertiary and they become lawyers. I would like to be that father that has children that are lawyers. My father didn’t have children that were lawyers because we didn’t finish school.

M: Alright. I know people have dreams. What is your dream and how far are you from reaching it?

P: It’s to have a beautiful house. There are beautiful houses I see and even ask where does the owner work. You find that the owner is a principal or a teacher. But I’m still very far from achieving that. I built a hut and a three quarter house. I’ve been saving money to buy bricks because I can’t be a young man that builds a house with mud bricks.

M: *(laughing)* You put it like that Sir.

P: I want to save more money and build another house.

M: Please tell me, back home they know that you are working, as a man how do they view you in terms of respect?

P: You get respect when you are working. I have noticed that back home if I’m not working and I go with my young brother to a ceremony, the old men will send me to do chores and not young brother because they know he is working, he’s not always at home, so he gets respect and not me. You see, things like that.

M: I hear you. How does your family view you since are the bread winner.

P: They respect me. I get my rightful position.

M: I like that part ‘my rightful position’ please explain further.

P: It’s nice to be able to meet the needs of the family and get the respect you deserve.

M: Alright. Being a young man and you already married and have a family of your own, where does that put you in the community?

P: It puts me in a respectable position.

M: I hear you Sir. I know in any community there are social clubs. Which ones exists here?

P: We have [place] funeral policy and also one here in [place]. As for soccer clubs eish! I don’t know because I last played soccer back at home. When I got injured here I stopped playing since then and hated soccer.

M: *(Laughing)* After your injury?

P: Yes.

M: Ok. Now please tell me ,these social clubs do you form them according to you clan or?

P: No everyone is welcome from all the clans.

M: So there is nothing like, this club is for Pedi’s, Xhosas or Sothos?

P: No nothing like that.

M: Ok. As you know there are different clans here, how common is it here for these different clans to get along?

P: The only clan that has a problem is the Tshangans, they always say we have apartheid but it’s them that have apartheid. We get along with Sothos and Tswanas.

M: Alright. We are almost finished Sir. I want to get to the topic of dating. I know other men are married here but have mistresses, how popular is that here?

P: It is popular. You know as a man you have needs so you must have someone on the side. That doesn’t mean that person is taking your wife’s place. It’s just a person to satisfy you in bed and then go in the morning.

M: Where are these ladies found?

P: Here on the street you can just meet someone you like and likes you back.

M: How common is it to get these women in the tavern?

P: I did it once but I was still staying inside the mine then. I got a lady at the tavern and she liked me. I bought her drinks and went to ask for a room from another man to just do a quick thing with this lady. We did our thing and left.

M: Ok. Would you say that’s a common thing men do here?

P: No it’s just a thing of the moment it’s not a common thing.

M: Ok. We grew up hearing from our fore fathers that men here use to sleep with young men. Does that still happen?

P: No, no, that stopped before I even started working here. But I also heard that it used to happen a lot. Where a men will even buy the young man a cow or give him promotion.

M: Buy that cow here.

P: Yes. But it doesn’t happen anymore.

M: When you hear what do you think caused it?

P: I think it’s witchcraft because how can you sleep with another man.

*(Background noise, man shouting at the children.)*

M: Alright I hear you.

P: Men eat healthy here so the hormones get crazy, so men need to release. In the mine when your wife visit they would give you a room in another block to get privacy with your wife. But now they allowed us to stay with our wives here.

M: Alright now you are allowed to stay with your families here?

P: Yes.

M: Thank you Sir. I am now finished on my side. I don’t know if you have any questions or something to add.

P: No I don’t have anything.

M: Alright. Thank you very much for your time. We will switch off the recorder now.

*The end.*
