# Supplementary material for: “You see this thing is hard… ey, this thing is painful”: The burden of the provider role and construction of masculinities amongst Black male mineworkers in Marikana, South Africa
Source: PLoS One. 2022 May 23;17(5):e0268227. doi: 10.1371/journal.pone.0268227 (PMC9126392; doi:10.1371/journal.pone.0268227)
Supplement: S1 Data — (ZIP) [file pone.0268227.s002.zip › Anonymised Transcripts/INTERVIEW 6_anonymised.docx]

**INTERVIEW: 711_0148**

***CODES:***

***M: MODERATOR, P: PARTICIPANT***

M: Thank you a lot sir, like I said... all this conversation will be confidential between me and you and the recorder I used it as reason of capturing so that I can get all this conversation for not ending up forgetting ,do you understand and miss all the information you trying to give me and after that ,you wasted your time and sharing...As I said sir, I would like to remind you sir that all this conversation is... for research only. Do you understand? There is.. there is no other reason I have that makes me do that.

P: Yes *(coughing)*

M: As I repeat those things I make it ...please be comfortable do you understand? Just helping us researchers to understand lives of mans. No' not thinking we have other intentions than this. No sir

P: Oh

M: I am going to ask you to be just comfortable and speak to me as much as possible ...so that we get information we need so that in order to understand men living here in Marikana ,you as men who are living in Marikana who have experience of living here and you as mans that living here in Marikana you give us the experience and tell us about it so that we look at it and analyse it. so that we understand and we can write a report about what is base according to the report you give us and information, do you understand sir?

P: Yes.

M: So we going to ask you to speak , feel free and speak little bit louder so that record.. the recorder can cache what we going to speak about, do you understand sir

P: Yes.

M: Thanks ...sir I’m going to ask you...(SOUND OF A BOOK OPENED).Sir I’m going to ask...ok...(SOUND OF BIRDS ON THE BACKGROUND)sir I am going ask your age what age are you?

P: My age ?

M: Yes

P: [year]

M: Ok [year]...about marriage, are you married sir?

P: Yes I am married

M: Are you married white marriage or

P: No, I am married traditionally.

M: Ok...ok..ok sir what highest grade did you pass at school?

*(noise in the background, cars hooting)*

P: Standard 7

M: So what time...when did you start living in marikana in which year?

P: October [year].

M: Alright , when did you start working in mines?

P: I started [year].

M: When you call yourself what tradition do you belong in xhosa,zulu or

P: I'm Xhosa

M: Ok sir, thank you a lot .Ok then…

(*the phone rings*.)

P: *(answering the phone)*

M: Thank you sir and can you please tell me about yourself sir, where did you grew up and about your family sir and the one you have as you are married and where are you are from? like... a person you don’t know ...your history let me put it like that.

P: Eeeh... I grew up in [place]...eeh...our father was working so...we were 7 at home so we studied and we gave up with school aaah we gave up with school and I started looking for job. I came to [place] looking for job...looking for job where my father was working. mmh...so...I saw I struggled with a job and I tried drivers licence...I passed drivers licence. I get into taxis...I worked in taxis for long time. Yes...until I married my wife and as time was going it seemed like the job is not right and I have children and I can’t be able to support my children ey I stopped working at taxis and I worked for myself and I hold on that job in order to raise children ey...by now the company retrenched us, we were looking now and going around, ok.

M: Thank you a lot sir. As you said you were 7 at home ,are you the youngest of the 7 or?

P: I’m a [number] born...there is my [number] older brothers.

M: Ok sir, what are they doing now?

P: The older one is not working and the other one is working in [place] in the mines.

M: Alright thank you a lot sir. You speak about you studied as you said you just drop in the middle in standard 7 as you were talking about you gave up, can you explained to me what happened that makes drop up in middle at school?, what were the reasons?

P: What happened to me? I impregnated another girl so I needed to pay damages so I drop the school that’s why I left the school I needed to pay ey...so I dropped the school that way and I never returned at school. I tried to work so that I can pay this child...I damaged other persons child. Since I dropped I never return again so I continued working until... I can't go back to school.

M: Thank you sir, to your older brothers what happened to them not finishing with their studies? or your siblings

P: Them... I can't remember what happened to them no one in them progress in school side, cause the older one is stayed is no longer working.

M: Thank you a lot sir ,life...when I am talking about living life ...how is your life at home you as you have lots of brothers at home not working because you didn’t continue with school? how do you continue with life? How do you get food?

P: Oh we are depending with children’s grant, we live with money. we have been helped by the children’s grant(noise, music playing at background)I can said we are living with children’s grant are the ones make us eat before we sleep because ey...jobs are scarse more specially for uneducated ey...we are suffering now...it’s much difficult...we are suffering a lot now

M: thank you about that sir, sir can we please talk about you and you not going to be hurt when I said you are this man. can you tell me about yourself as this man you are coming from this family you have and you have your own family now you raising your own children...the way sir that makes you put food on the table, I hear you speak about grant, what plans do you do in order to put food on the table can you tell me about your strikes what do you do sir to prevent hunger.

P: No I’m trying... sometimes they call me there is a special job and go take that job maybe for 2 days and 3 days and get that money bring it at home so that children can eat ...and get that special job.

M: Sir how does this suits you, you as man?

P: It hurts...it hurts its pain you wish seeing yourself getting a better job so that you can raise your children in a right way... this is pain it’s not nice but you have to endure. We endure because there is no other way ey.. it’s not nice (MUSIC PLAING AT THE BACKGROUND)

M: What motivates you in order to wake up every morning as this man saying is going to be better. What makes you have that heart?

P: Yah if maybe When I get that 2 days I told my heart ay no it’s not the same as long I bring something to children .for only children to say their dad bring them something at least eeeh... I give myself hope that one day I will get a right job children are growing well ey...

M: Can you tell me about your children and family that you are living with here can you tell me more about it? Who are you staying with a...

P: Here...here I am staying with them .there is also brother in law that I’m staying which is working but not permanent in that place but still holding and him he also help us with something to eat and buy groceries cause we are living together here.is the one called earlier on yah

M: How do you stay together sir?

P: we stay right a lot, we stay right a lot and there won’t be something we might cause disturbance in between each other

M: Ok sir I here you. What are your children doing?

P: aah...my children aah ey...cause there is this one who can’t walk so that one is disabled can’t walk, can’t talk... so she was sick when she was 3 months ...2 moths old ,as a baby she grew up and grew up, we thought she is going to be right, no...baby is still not right we took her everywhere coming from [place], [place], there’s no place we didn’t take him to but he didn’t become well *(noise background people speaking with each other*) is still disable ay*(crying*) ay... it’s that situation

M: I hear you sir, so what...are they going to school or what are they doing?

P: others are going to school which are at home.

M: ok sir lets continue about manhood, how do you see manhood as this man you are sir. You look at what important things as a man, as you this man you are what makes you feel you are strict to your children or to your wife...what is important that a man should do in his family that is showing you are a standing man?

P: Important things you should do, the main is to work, to work do you’ve see when working you have to support your family ,must not suffer but you working that’s main aim that. You have to look after your family and your children must not struggle, your children you must take them to school, try by all means your children must success at school and your family you look at it ,but your family is suffering but you are working put hands there and there even if it’s not there one you are living with, you have to guard your family... that’s men's responsibilities that yah so that you can have things you wish to have that are important not staying in taverns, staying in sheebeens...that’s not a man that, hats not a man that that’s are important things ,raise your family when working yah, raise your family and guard your family. children at school...school it’s very important than everything in the world there is no important thing than school as I look as a man I am and now I still crying for myself. if I didn’t have children I would return at this age which I am...school ay no I still crying by now for it, because if I go here you would hear they want grade 12even here in the mines you can’t GO IN IF not they education first in everything. its if you struggle ,struggle but educated education is important these days a lot, lot, lot there is nothing than that I, didn't see it. Nope I don’t see it no I don’t see it.im saying every day to my children ey school...there most important thing is school

M: As you said sir, as man you do important things and you put man have hopes you live as man with hopes, what kind of hopes is that? You sir which hopes don you have in life to be a man which is older and say maybe hopes are fulfilled as I have as man which hopes do you have as a man?

P: My hopes when I can work. If getting a van, getting a bakkie and have proper house, having sheep, cattle, pigs etc.*(noise background)*have a good condition bakkie that’s my hopes if I can get proper job.

M: Why are those things important to you sir?

P : They are important because those things can make me live right when I’m at home not working. And raising my children I go anywhere they want to go. I won’t struggle. that’s my wishes if I can get those things, if I don’t get bakkie ,get at least truck, I can be successful ,my children won’t suffer if I can have at least a truck.

M: In families sir there are roles, woman’s roles and man’s roles, which things do you think are your responsibilities ,which role do you play as father at home and you said you have big family in [place]. Which things as a man, can be on hold when there is something at home and you are not there?

P: things...like my brother is unemployed let’s say maybe he want to take his child to initiation school so his child is also mine so I need to help there to put hands try to help my brother because his unemployed and I’m working, I’m making an example I supposed to help him there yah so that his ...his child thing progress right things like that I should look at it as this man I am, so that I can help were my brother is failing I need to correct it yah not saying he must see by himself but I see his not working how will he do those things while he is not working you see he was not like that on purpose

M: For you sir, I see all your hopes are beautiful and alive, you see what you want to do on your family how difficult it is for you...to fulfil your hopes, really, really on my heart I wish I can find a right place so that I can fulfil my hopes in my family like now there heart is speaking although I can’t do anything there heart is speaking ey...if I had a right place I could help you there my brother there and there.

*(music playing in the background)*

P: Ok I hear you sir ,can we please proceed sir ,I hear you, you are a man who have his own home a wife ...is there anything that might cause conflict in between you and your wife, and tell me when things like that occur, how do you solve it in your family ...with your wife and when you don’t see things eye to eye?

P Yes ,fights like that occur... I call my siblings and they come and discuss with them ,take decision when we have a fight with my wife...call them ,if its difficulty I go and ask fathers law to come and solve that, if we failed to solve it.it gets solution by that way. I will not say no, I will fix it by myself. Call them and tell them hear we don’t get along about this and this and this and I put it ,talk about it and fix it till it’s right when something like that happened.

M: What makes you like to call people to come solve it, cause other people would maybe do it the other way, why you ...you choose that way?

P: What makes me choose this way...is that solving things alone you break marriage...so this person you speak things to her that should speak because anger...anger raised and speak things that might cause this person try pack clothes and go... so that way doesn’t build a home it destroys it...so that’s that reason makes me call a family to solve this

M: I hear you sir, if sir there’s a problem hear in the community or conflict or problem with your neighbour or at work...other man there’s a conflict it speaks to you not the way he should ,how do you solve that?

P: Do you see what you talking about happened yesterday hear, yesterday hear we were staying there was this child from back home staying hear across the street she was hear yesterday ,we saw other ‘makoti’ *(woman who have just married traditionally*)she was very in a hurry and she turned and...this child said I know this lady...I say do you see my child the way this lady going is hurt, and there’s someone who hurts her and now is going to a place that is forest ,she is going straight to the trees do you see? I said my child step aside this person have a problem she tried to run after her and shout when she was there, she just look back and proceed so I said run after her they went deep to the trees and her also we didn’t see them after then disappear it stays long time they come back at the sunset when it was becoming dark. She starts at home when she was there she found out her husband wasn’t there. She brings her hear and we ask her and told us there were some problem with her husband, she is the person that you can see she is pregnant, so there was this conflict. I ask something and her husband to be called because what he is doing is wrong to this kind of person. Her husband arrived... now they talk about their issue, we try to show them the right way...right life is this, and this talk to her husband don’t do this worse and worse this person is pregnant maybe sometimes pregnant woman trend to want this ,this and this or don’t like you and just not loving you(laughing).we solve the issue and we see it seem nice now and they get along .About what you saying if comes things like that you asked people to come talk ...life situation is going like this and this right....when you see people interrupting each other call that peoples family to come don’t leave them and resolve the issue, do you see

M: When... I hear you sir...if situation a man comes straight and come with disrespecting you I putting like that sir what do you do sir? disrespect you with your dignity

P: I call other... call neighbours ey there is this man and this man is doing this and this and this, me I don’t want to fight and if I fight it’s a jail ...I’m going to jail I don’t want to fight if I fight with him that’s cases that do you see ...I’m going to be involved in cases, so I call the neighbours to tell them this man is doing this and this, so neighbours talk to them and resolve it, you are coming to corrupt this person and I stayed calm for the law cause I don’t want problem to go to court talk cases

M: Sir I am happy we are progressing very good, thank you a lot, tell me sir as man who is living in this community, when you have a spare time ,when not working or not at home meaning your free time how do you spend it?

P: My free time I clean the yard like as you see that place with burning papers... I pick up that papers and burn it .clean the property ,repair the yard or repair what is not right in the yard clean the house also and nothing...if maybe chat...go to my brother in law chat with him and is staying down there in the hostels and there’s nothing more if I go to sheebeens no' I don’t that’s all. I just go around here .That’s my free time. I stay under the tree or go to the house and watch TV that’s my free time, I make tea and drink it ,there is nothing more.

M: Sir you said so in the beginning and you said something again about sheebeens. Why it’s like you push away the sheebeen thing?

P: Ay the sheebeen thing as I see it...it’s like it takes you nowhere, it’s like taking you to hell. Sheebeen it’s like is not right place to me, yes. I don’t know if is just because I don’t drink. As I see sheebeens is not right place, is not a right life, yes I don’t know if it’s me seeing that cause I don’t go there or don’t drink maybe... I look at it as if I will go there I will suffer band my children will suffer going around begging for food .so ...no sheebeen I don’t like it(laughing) no I put a big X to sheebeen. that no

M: What...maybe here in the community men...what makes you hate sheebeens? ...tell me about men living here as you say you are going around the house repair there and there. What other men leaving here doing?

P: No ...if they staying...going their yard you will arrived the yard is dirty ,there’s nothing beautiful inside, no hear you can see it stays alcoholic ,you won’t arrive the is right. Found that persons children suffering because money being spend in sheebeens, children achieve nothing and his wife suffering don’t get money because ending in the sheebeens you see so ....me sheebeen I don’t encourage ,I know we go there but I don’t encourage it even if you struggle don’t go there...no I don’t encourage it

M: How popular is that here as man who is been here for long? How popular for mans who is working or let me say man form here who is coming out of work ...weekend their free times how do they spend it?

P: it’s too much popular here, especially month and hear what is playing, playing the playing(music playing loud meaning)full you can’t even go there inside ,yhoo you can’t be able to go in ...wha whaa whaaa(meaning music)yhoo you can’t be to go in (laughing)its full inside, month end its to full .spending money forgetting about the family, forgetting about the family at all. Person coming with a thousand rand coming back with nothing ...a day come back finished oh no

M: As you stay here you know we are mans ...especially we are drinking mans...when we go in this places we have side girls that we get on that places how is that life here?

P: It is that life here, its other life that is much here...its other one that is finishing them side girls, its worse .yes ey... they are full in the sheebeens they are going with them in the sheebeens, that's worse that...this life you talking about now is worse,worse,worse worse

M: When you think what makes it worse?

P: its popular because they say there is too much money in the mines.so those ladies...they come from different places as...when (union organisation) get in here and the money raised...other day I hear quantum arrived in Marikana its only ladies its coming from [place] this quantum(taxi)throw those ladies in the in the train spoor they say they are coming in this place with a lot of money, do you see they coming for business here those ladies so I mean...

M: Which business can you explain to me… non-citizen come here and they come with what intentions?

P: They sell bodies cause they want money from man, to sell bodies...cause...as a man we have a weak point when it comes to ladies. people which are using their minds we don’t know. As you see me as I arrived 2009 I never have a side girlfriend and I will never have it and I don’t think I will have it and those checking places I always go and I’m not scared to go there. If I see something like tent checking high blood, HIV, I go running these places and I’m not scared even if you say we can go now to text I fear nothing , I’m not scared

M: What makes you not scared?

P: Is because I know I’m taking good care of myself, I don’t go fool around no I’m not scared(laughing)I mean life of here is that situation...lot of man who are working they lose...those ladies who now a fulfilling here because a person ending up not supporting their families ...she have this person now that he is staying here which is not her wife, because lot of them they are staying with them now like is her wife and cook for him do everything lot of them do that ,they stay with them they stay with them in their houses it’s like a person wife

M: Mean fulltime here in the house?

P: Yes, fulltime and cook for him go to work come back been at home. People do that here a lot of them, they stayed with them

M: When ...maybe when the wife is coming back what?

P: When wife coming back take side girlfriend to rent for her other house take her out of the house where the wife is knowing ,rent for her somewhere.do you see that is beaten that, he rent her and rent for that person, children back home needs money, children needing support, money for school.do you see that waste they are doing, it's a waste more than a waste(laughing)

M: I want us to go quickly at work as you worked here in the mines, for you how do you experience working in the mines?

P: Ey me...working in the mines I don’t feel nice about it, it’s just to endure because of uneducated because it’s not a nice place, it’s not nice its we endure cause you need to raise or support your children other than that it’s not nice.

M: Why it’s not nice?

P: You work hard, you work very hard

M: Can you explain why you say you work hard?

P: Do you see that long cement like over there, there is something like cash over there that your climb on, you stand on that thing. that thing takes 100 people, so that thing it goes with one rope that looks like...electrical wire so you used that thing to go down...it’s a risk ,if one day this thing could breakup, it’s end of life. And again were you going it’s just a stone ,water, their wind is a force, no sun, no wind, their air is ventilation is own made with force, so you walk over stones can even fall for you any time and fall over you ,beat and kill you so it’s not a right place but we endure because we are not educated but it’s not nice to work in place like this, it’s just for only families and children in order for them to eat. We endure otherwise it’s not nice. When you in side you are dirty, dirty, dirty your clothes filled with oil ,do you see that clothes we wear, you will never wear it here. There are special clothes that we wear there we undress as soon finishing working and leave it just there, they stay there...you have to change and wear other clothes when coming here ,you will never come out with that clothes cause you will be filled with clothes and leaking water...sweating while working there you won’t be normal as us if you are working there like now(laughing)it’s not nice. When you see them driving you will think they are working in the right place, you can see it’s not a right place ,it’s not place to work people, it’s not nice just enduring. You end up seeing ,how a man can work to a place like that, working painful like this and again end of the month your money which you worked painful for it going wasting it in unnecessary things, useless things, 'yeerr!!'*(angry*) it’s just that other men don’t think, sometimes you ask yourself this person eating his money like this after all this hard work and you feel sorry for him and think he might have been given the money to his family to do better things than this ,do you see? By those words it’s not nice we endure. It’s not nice at total.

M: Sir as a man ...as man who is working how does a community look compare to those working with those not working?

P: People like that they look at that person and said that person he should be working but we and up similarise that person with the one not working sometimes just because of waste he is causing but he is working, things like what we have just talk about, there are people like that and see this man he should had work but his money wasted there and there, it’s just that there is something beating this man and is beaten there and there

M: when....I hear you sir, do you know dignity in man, is it giving dignity working as man working in the mines, do you get dignity in the community and back home in [place].

P: yah, yah, it gives you dignity. You do get dignity but your dignity is to do things that gives you dignity and if you do not do things that doesn’t gives you dignity people will comment and say yhoo! yhoo. This one working in the mines Johannesburg but look what he have? What is he doing with the money? they laugh at you, do you see ,things like that as a man it’s not wanted. What is wanted from a man is that when working things you do must be visible to people(must see what you doing with your money)there must be things that will make people give compliments to you. Like Mawawa is working really guys do you see he have house there are his children going to school, do you see ?, he doesn’t fail on raising his children, have own cattle so a man must be seen with those kind of things but if you have nothing we going to gossip about you and say this man ,what is doing in that Marikana? ey we don’t see what is he doing there it's the same as us sitting here. which means no dignity if you are like that, you have dignity with doings things you doing people must see it and then you will get dignity and start saying ey...Mr [name] is working we can see him ? He doesn’t play there in marikana. Man must be seen by his works.

M: I hear you sir ,can we please talk about experience of 2012 about the shooting of workers here, I know sir that you were that time as I look you arrived 2009 here .can you give me your experience sir what happened....how, what did you see there?

P: It was painful that, as I see cause really I was nearby, I was there...me what I saw ...people were just sitting there going to do their meeting. While people were sitting there I saw police arrived and arrived inserting a tape, they close with that tape and people were on other side ,police told people to move from there and people said no we are not going anywhere, after than I saw police putting raise wire and me I was near ( blowing noise)I told myself I’m going now when was little bit far I heard a gunshots and when I see it was dark hearing sound of gunshots, shooting ey, others were beaten by the water and I run I never went nearby, no I never went closer again. I heard mostly people fell there and saw it later that there are mortuary cars coming picking people there ey, it was that. I never go there again so it means I saw that thing like that ,I just heard that and that but I never went back there.

M: Sir about your experience as you were there, those people were sitting there and why and what do they want.?

P: They wanted money, they were mourning about money. They wanted 1500 in their pay their demands was money shame there’s no other thing. They were not fighting with someone only complaining about money. They wanted money.

M: As a man sir who is working ,living here what did that strike mean to you?

P: EY it makes me cry....if maybe I got a job but not in mine ,I should had gay it hurts me a lot that thing of dying many people like that (CRYING). It made me afraid that thing of dying a lot of people I don’t want to lie. it touches me very bad

M: How were you feeling sir?

P: It made me scared a lot of people dying ,dying for their rights and not beating anybody.

M: Sir can you tell me where you there in the build-up of that thing? As you see why that strike took so many months was it nesesarrly.5 or 4 ,how people where they feeling the man as you said the man must do something in order for their families to eat, how the man were saving how did happened that time?

P: It was bad that time as if...hunger , people were starving here and been helped by company called give us(Dr give us)(noise children shouting),it was really bad people will die cause of poverty now, It was a bad here people were helped by ‘*give us here’*

M: I hear you sir I know people used to work suddenly not working now for 5 months and there’s nothing to eat now what makes men hold on that strike while they see poverty is here?

P: What makes them endure is that they want increment in their pay they sacrifice and tire their self they want this increment their employers put it. That’s why they trouble themselves. cause if they can move back they increment them and what they want they shouldn’t get it that’s why they hold on for that time until they get want they want, really at last it was like that.it just went better ...so they see the increment.

M: Sir there is something I’m going to say that I want you to give me your opinion.as you see on TV and newspaper was it there something of carrying weapons by the workers ...on your opinion do you think other mans that lives here did you see any changes to the men get like lions you know what I mean sir, like this man is polite and turned to be something?

P: Those man have been changed. There was a man like that, those men told themselves no matter what can happen they don’t care. they don’t care now, they throw themselves. others were scared to go nearby, others were forward and see that they are throwing their selves now *(child playing at the background making noise)*

M: Sir tell me…we are almost finished. We will finish just now. I heard there was a thing of men dating other men here in the mines…

P: Yes there was a thing like that, especially down there underground.

M: Underground?

P: Yes. I never heard of it here in the location. That means it happens underground.

M: what do you think was the cause?

P: Ey that thing I really failed to understand it.

M: What made it to stop now?

P: I think it’s because there are women now in the mines working with us underground. So now I’m sure they do it with these women underground. They are down there even now, maybe doing it there.

M: *(laughing)* Down there?

P: Yes I think they doing it down there.

M: *(laughing)*

P: It’s like that. And now i haven’t heard there’s a man dating another man since there’s women now.

M: Alright this is my last question now. You’ve mentioned the issue of mistresses and you even put an X on it. Now please tell me how does that work? What’s the arrangement, is it about money or?

P: It’s about money. These women are here to make money. A man stays with a mistress and spend money on her, lots of money. You find that when this mistress is going home this man buys her a bulk of clothes and food and those things are going to the mistress’ home. And when there is a ‘stockvel’ money coming out, they divide it in half. If it’s R 20 000.00 this man gives the mistress R 10 000.00. And his family back home is suffering. That is not a good thing at all.

M: So they spend money on mistresses?

P: Yes. And these women are here for business.

M: Please tell me this a temporal thing or they end up staying as a family?

P: No, it’s just a temporal thing cause she gets money and then leave the man to go find another one with money. These women are here for money. They go with whoever has money. I will be staying with a woman here, but she doesn’t sleep with me only, when I leave for work she leaves after me and go to another man and when she knows I’m about to come back, she sneaks back as if she didn’t go anywhere.

M: Yho! That’s how they do it?

P: Yes. They coming to spend the money of these stupid men. Cause there are a lot of stupid men here, that don’t know what they are here for.

M: *(laughing)*

P: It’s better when you are spending money on your family than finishing it on mistresses while your family suffers.

M: Now as men on the right standing, how do you view men that do that?

P: No we don’t look at them right, we don’t look at them right at all.

M: I hear you Sir. Thank you very much for you time and for welcoming me so warmly making me feel at home. Is there anything you would like to add or ask on what we were talking about?

P: Yah!! But I don’t know if you can include it in your report. It’s the electricity issue, we don’t have electricity here, we are struggling. And now we are doing the illegal connections, which is not right. Don’t know if you can help us there by including it in your report.

M: I will note that Sir.

P: Thank you.

M: Thank you Sir. Are we finished now or there’s something else?

P: No there’s nothing else.

*The end.*
