# Supplementary material for: “You see this thing is hard… ey, this thing is painful”: The burden of the provider role and construction of masculinities amongst Black male mineworkers in Marikana, South Africa
Source: PLoS One. 2022 May 23;17(5):e0268227. doi: 10.1371/journal.pone.0268227 (PMC9126392; doi:10.1371/journal.pone.0268227)
Supplement: S1 Data — (ZIP) [file pone.0268227.s002.zip › Anonymised Transcripts/INTERVIEW 7_anonymised.docx]

**INTERVIEW: 711_0149**

***CODES:***

***M: MODERATOR, P: PARTICIPANT***

M: Thank you [name] for coming’ relax feel free it is just a conversation between two men.

P: Ok.

M: How old are you or what year were you born?

P: I was born in June [year].

M: Are you married? If yes’ was it traditionally or a white wedding?

P: Yes both

M: What is your highest qualification?

P: Standard 7.

M: When did you arrive in Marikana?

P: In 1997

M: When did you start working in the mines?

P: I started in 1997 around June, I’m still ok working there.

M: What nationality are you? Or what do you call yourself e.g. Xhosa, Venda or Sotho?

P: I am Xhosa

M: Thank you [name] I first wanted to get your background information, we are now moving to the next section.

P: Okay

M: Please tell me anything about yourself, your family, where grew up, and how you grow up?

P: I [name] grew up in a Village called [name] in [place]. We are a family of [number] and I am the eldest. I had to go look for a job because my family depended on me. That is when I decided to come to work in Marikana and I am still here working. I had to work so that I can provide for my siblings. As I was working I started a family of my own, I have a wife and three girls.

M: Why did you feel that you have to be the one who has to take care of your family back home?

P: My father was working on the road and was not earning enough to take care of us. I felt that I was matured enough to help my father to take care of us things were better, even my sister who was born in [place] became a [professional]. My two brothers are also here in the mines, one of them is also working at a mine called Lonmin and the other one is at [place] then my one sister got married and is staying whit her husband in cape town whilst the other one is at school at [place].

M: [name] I don’t know how this is going sound but how did it happen that you leave school at standard 7 and what are the reasons?

P: What happened was they never said I must stop going to school. I could see for myself that I should stop then I asked another man to look out for a job for me this side, he then said I should come, they are not hiring at [place] you are can stay with me at the hostel the man said, and I left home. While I was here I wrote a letter to my father because letters were written at that time to let him know that I got a job that side. Everything was okay I got hired at the mine and my father happy that his child is working. In 2007 my father died, I buried him, and I was left with one parent.

M: Oh ok thank you [name]. Another thing how important is it to your family and you as a man that you were working at that time and at that age? Hence you say you had to come this side so that you can be able to get a job. How was that important to you as a man [name]?

P: Hmmm it was very important to me that I get a job because my hopes was to have a wife and a job so that I have money to help where I can.it would have been bad luck if I’d gone another way and not get the job.my dad would then have been very disappointed in me and said I took you to school then you chose to go your way.

M: Ok [name] I hear you now but I would like to know for you to be seen as a an honored man what is it that you are supposed to be doing or for people to see you recognize you as an horned man I’m not sure if horned is the correct word but i mean to say a respectful man.

P: For you to be an honored man you must be married, A man that is married is always followed by children. Then when the children are there they will call you father, which means they are scared of you and you also need to set an example and all that makes you as a man feel honored. You become an example to them and they can easily say there is our father and not call you other names. You must also have life stock in your yard like chickens, cows, sheep you name them so people can see this is a home belonging to a man. Having life stock also puts you in a position to say something in meetings amongst other man, because you are also a man now *(laughing)* then you would hear them say we think we should go with what [name] suggested. Your voice must be able to be heard and you should appear to be a man working towards something that can be noticed.

M: What are the things that you are supposed to be ding in your community that makes people or even your family see that you are a man amongst man or let me put it this way what are you as [name] doing that can make you say I’m a man amongst others?

P: When you are a well-respected man or an honored man when any kind of problem arises people are being called to come together whether it is a family issue or a community problem for instance if someone has died this person had no children or no family then we as the man of the community comes up whit some kind of an arrangement that can suit the situation where I live we burry with a cow then I as a man that has life stock would just easily say no you can have the cow whiteout any payments then in the future the little that you have grows because you are a helping hand to the community and your family.

M: [name] for you to be able to be able to reach all this targets as to watching over your family ,your house hold the community ,working in the mines and plying your manly role what is it that you do or how do you do all this things at ones?

P: sorry I did not understand.

M *( repeats previous question while [name]’s phone is ringing)* we can take a break now. Are you ok [name]?

*(after a while)*

P Yes I am.

M: Alright [name] now I am going to ask you a personal question. How do you manage to do the things that are expected from you?

P: Well I can say it easy because I am working now, I have a stable income. I had my cousin went to work in [place] and passed away there, I had to make means to get him back home. I could do that from the money that I saved for emergencies. I then took R10 000 from the emergency money and bought 6 sheep and the funeral went on. I was seen as a problem solver.

M: So when you do these kind of things, how does it make you feel as a man?

P: It makes me feel good and respected. My family knows that they can really on me when they have problems

M: Thank you so much [name] we appreciate you being so helpful. But we are still going to continue with our talk. I would like to ask you decided to take a wife but in relationships we are likely to go through misunderstandings when you go through these kinds of situations what do you do.

P: Well we find ourselves in these kind of situations but I always find ways of dealing with them in polite ways for instance I’m a man of god I don’t drink but sometimes she does drink then I just not say anything until she gets sober. Because it disappoints me to see her drunk because I am a religious man and my wife should follow in my footsteps together with my children.in some cases I don’t wait till the next day or when she is sober I have to then sit her down and resolve the matter especially when history repeats its self. When it gets difficult to resolve I then have to call an older person than myself then the three of us sits down we let the elder speak .if nothing changes we than call our parents and let them take it from there.

M: [name] what do you do in cases where you feel someone is disrespecting you or not speaking to you the way you would like to be spoken to like in your work place, your home or even in your community?

P: I just distant myself from someone who doesn’t have respectful ways of talking before it could get any worse.

M: Distancing yourself from someone because they disrespected you in front of people may result in you losing your dignity why do you choose to handle it in such a manner?

P: I handle it that way because I wouldn’t want to end up in jail just because I wanted to be a winner I never want to go to jail and let my children suffer *(laughing).*

M: *(laughs)* Yes I hear you [name]. You have been here for quite some time now its 1997 if I’m not mistaken you work and you must rest well so when you rest how do you spend your time like when others are out drinking or ball playing or watching football what is it that you do?

P: When I used to drink I suspended my free time with my friends there at Marikana west we would buy our drinks, put them in the cooler box get in the car and drove off to have a good time but now that I don’t drink I’m in church all the time even on Saturday’s .when I’m not in church sometimes I go visit my brother there at Marikana west.

M: In the time that you’ve been here from when you were drinking and now that you don’t drink what differs according to the way you see things?

P: When I was drinking I was out of control because most of time I was drunk me and my friends used to work night shifts and when we come from work we would go to [place] then we would come back on that moment we sit on our money(laughing)we eat our money. When you get home you realize there is no food and there is a tense vibe bear in mind you must be at work Sunday because we used to go to work at Sunday night for Monday then there would not be food you feel exhausted then you decide not to work now at work you end up having a bad record because they knew by now when you’re not in work on Sundays you have a hangover *(laughing)* and my friends always made fun of me because I used to do things that I don’t remember when I’m sober so I decided to rest a while and not drink then I tried going to church when I realized that I was better off without drinking.

M: When you were drinking did you do the things that were expected from you like taking care of your family you being the oldest one or did you neglect doing what you were expected to do?

P: Yes I was neglecting my ditties a lot because there would be things I plan to do like for instance extending my house I would by all the material than I would run out of cash because I still have to get sand still have to pay the builders but because I had to drink and give money to my mistress and take money home for the house hold I would end up not having enough.

M: Let’s talk about the kind of life where you have to leave your partner to go work far or where you meet someone else for someone you met here have you experienced one of those cases and how was it for you?

P: Yes I have experienced that but in my I case it was different I worked far from home but we had rooms to stay in I went home every weekend and that was a problem to me because when you stay in two different places you have to be responsible for both while the children on the other hand also need their own things maybe school shoes which are expensive yes we can always get them fixed but not fix them till they can no longer be fixed(laughing)so I mean it’s best to go with your family to where ever you need to wok so you don’t rent two places or buy grosser for two so that you can budget better.

M: How did you manage at that time and how did it makes you feel to have to rent for two places then again taking care of your family?

P: It made me feel bad especially when I get a call from home asking for money I did not have because now back home they know I got paid and I haven’t giving them anything because I don’t have I had to provide for my other lady her needs my needs and her rent. Now I hear reports about my children needs this and that it makes heart ache very much.

M: How often does it happen that people here in Marikana are having double families and why do you think people are doing it?

P : Well it is because people live far from each other for too long and there is no space provided for family’s now even whit your first salary you can’t go home because its far and the money is just not enough.

M: Is it a popular thing here that people have different wives?

P: Yes it is a very popular thing because most of man here has other women but those who don’t have you can count. It’s not something to hide.

M: These lady’s for someone who is not from here where can they be found or where do they come from?

P: The one I stay with here calls the sister, the friend, or the cousins saying they must come things are looking good here so that is how this lady’s comes here. When they arrive someone is organized for you then you stay with your partner.so does the next and the next.

M: What does these man end up doing for these women in these relationships?

P: they rent the places they stay in with these women they provide for food they buy them clothes and gives them money for things they need on daily bases the comes times when the women has to go back home for holidays still they have to be given some money so that their family’s back home also gets something then again these women has something called society groups where money is being add together the whole year until the last month of the year then the money is being split the last month meaning that these man have to provide for that monthly fee as well .So this means you are making things difficult for yourself because this means when these women get pregnant you have to feed that child also while your wife on the other side does not even know about this children if you don’t support the child they hand you in for child support and you still left with no money.

M: Thank you so much [name] I hear you on that one I think man are really faced whit these kinds of situations, because of working issues [name] take me back to the mine working issue. You’ve been working on the mines for years now so what did you experience working on the mines meaning what would yo say to some ones that has never been on the mines?

P: Well when I got here I was really surprised because I had to go into a small hole like a mouse because a mouse goes in this hole and gets through on the other side I never thought those holes were meant for us I was shocked when I was told to get inside first I refused thinking it was a joke but at last I did then there was this heavy objects I had to take from the outside and take them inside they were meant to keep the walls from falling and I head to take 20 of them inside but when I reached the 10nth one I was not sure as to whether I was going to make it because it was really hard.my left shoulder already had bruises because I was using my left arm to carry these objects but I went on working knowing it was my only options because I didn’t have the best qualifications for other jobs I knew my family depended on me. But after 4 years things got better and I got used of my work .as time went on I decided to try the driving job I went to the boss and told him there were tests I needed to go through and also something called the ‘*dova’* test to check how slow or how fast your brain can be the secretary filled out some documents and so I did driving test I made it so I got the job the other thing that made me leave my previous position was because we used those light hats to see if you light went out in the middle of that dark mine you had to sit right were you were and not move because of the dark.in 2002 my boss gave me a higher post which was a supervisor post I now had to watch over the drivers I went for a training and I past my test and I’m still a supervisor, and the money is better than before now.

M: You said that the money that you were earning before was very little what made you stay?

P: There was no other way because the other jobs that were available was better jobs that needed qualifications and I did not have those qualifications. So I had to sacrifice until things got better for me.

M: [name] you coming here to the mines to work for so little money I’m sure you had dreams what were you dreams or what do you want to archive?

P: I would like to build my house with block breaks so that even if it rains it does not fall and I would love to have enough life stock and a car so that I don’t have to walk by foot. I also would love to have some horses and children also which I have and if so happens that I should be at home then I’d love to have taxis especially when you have been pensioned.

M: So how far from archiving your dreams are you now?

P: A house I have children I have but only girls I would love to have boys. The other thing is that I feel like getting myself cows and sheep but I want them to be here where I live with my wife because most of the time I’m here with my wife meaning there will be no one to look after them back home if I get myself a double cab Bakkie with a trailer whenever a recusal issue comes up I can get back home with my cow or sheep on my trailer. I had a private small car but it rolled over but I was lucky nothing happened to me.so now I would love a bigger car as well but the best thing I have archived is having built my own home because a home is the best thing anyone could hope for and it’s important to have one.

M: You’ve mentioned something about you having three girls and no boys and that you would love to have them how important is it to you that you have boys?

P: Because when you have girls only they are being married and has to leave home where on the other hand the boys marries and brings their wives home meaning without boys this house may one day when I and my wives dies the home dies too because no one will come visit an empty house.

M: So in your man wood how important is it to you that you have this boy since you are the oldest at home?

P: It is very important that you have a boy so I must try my best to have one. Like me at home if all of my sisters have to go I would be the one still running our home so when my sisters wants to visit then it’s open for them because there is someone or if things does not work out at her in laws they don’t call her names like you don’t even have a home.so I must have a boy.

M: The fact that you are working at the mines in what position does it puts you amongst the other man that are not working back home does it makes you a better man or not?

P: You are being seen as an honored man because you are working and you are not always there so when you go back home it shows that you are working because you are different from the man that are always there.

M: Thank you so much for being relaxed and be so kind but I would love us to move back to the strike and the tragedy that took place here in Marikana in 2012 I am sure you watch the news everyone knows about it and we were all shocked I hope you were here when it happened now I would like to hear your view of that strike.

P: That strike was not supposed to happen because it was not the place or time to strike about money issues it was not a legal strike but because we had money issues we decide to go to the boss and talk to him about raising our salary.

M: Yes.

P: So there was this one man who was one of us as workers who said no man, we have a union and if we go there without our union those people are going to ask where our union is and chase us away. Five men were sent out to go to the union offices to go and talk to those people but they came back saying there was no one in the office so we decided to go to the bosses which was in [place] in [place] ourselves.

M: Ok.

P: We went there in huge groups but when we got there they have already planted danger tips. Somehow they knew we were coming so they told us not to trespass and the security also told us to stand back. And they asked where our union was. Our union that time was [name of union]. We told them they were not in the offices, they then said go back to them. So we went there with no luck then again all of us came back to them, and this time it was the whole squad. So when they saw us coming they thought we came back to fight them so they stroked first, and we all ran away and we gathered on the field by the gate.

M: Yho!

P: They were beating us up and the boss said we should speak to those people who were there with him, but it became a problem because now we did not know who to speak to. While standing there we saw police vans and the security cars coming from where we were standing, you could see something coming from far but closer to the shacks and bushes. When they come you can’t see so we could not see where they were so we went up to the top of the mountain so we can see everything going on from there. We sat there by the huge rock. No one went to work we were on strike. Even where we were sitting we were not welcome, they came there and told us to leave we refused we told them to ask the boss when he will raise our money because we needed answers and we were hoping for an amount of 12 500 .00 so we can fix our problems at least that amount was going to cover our needs.

M: Yes, yes I hear you Sir.

P: We were told that the boss can’t come here because he has a group he is sitting worth this is not the time for him to be talking about money the police said we should leave that place we told them we are not going anywhere because you chased away from there now you are chasing us away from here again so we sat the all day long some went their own ways but came back again because everyone had this pain *(noise sounding like a train*) we were on our own now our union was not helping and we were an end member because the [name of union] was in [place] and was a very small group so we asked someone called [union leader] there in [place] we asked him to go to the union on that side and that he should come with him so he can talk to the boss here and ask when the money is going to be raised and when he will deposit the money. The people from that side said it is not possible for him to represent us because it is said we are armed of which we had no choice but to sharpen the spears we had because the police already fought with us when they arrived. So at last Mr [union leader] was told not to come to us for feedback because we are going to kill him but he said no he will come to us and give us feedback so he came and explained to us what was said and he told us that even his union cannot represent us because we were no members of them therefor it will be best if we all go back to work then we can join his union. He said ‘leave this place because what I have heard there is that you are going to be shot here so I would not like to see blood being poured and families suffering so please leave.’ We said to Mr [union leader] he must leave us we’d rather die here if we must die we won’t leave here without answers what is so difficult for someone just to come here and give us answers because this person that is here has point. Where people lost their lives on that day of the 16 2012, the next morning the 17^th^ we were told we must go to work we were surprised because people died just the day before.

M: Yes, I hear you.

P: We refused we said they can come and finish us here and see what happens then so our president was not welcomed here because we went straight to those people first and he never came to the people like he was supposed to. He is the president of the people but he failed to come to the people. We only saw on the news that the president was here we never saw him. The person who came to us was Mr [politician] and we appreciate that a lot because as soon as he heard what was happening in Marikana he came and he listened to us and he said ‘AMANDLA’ to us. As for MR [president] he came too late because we were already gone, but we heard he spoke to those people and he promised to fix the whole situation and that everything was going be rectified around that time but we are still waiting for his promise. He has not come back to us *(laughing*) We were promised houses but those houses were built in Marikana those houses were meant to be built for the workers because they were only built after we complained of not having places to stay. These houses are causing a lot of misbehaving because people stay in there by force.

M: Thank you so much [name] I like speaking to someone who was there. You said Mr [union leader] said you should leave there then people responded they’d rather die there how were these people feeling were they afraid why would they prefer to die there when they know they were going to be killed?

P: They had nowhere else to turn to the only way for them was to better die for what they wanted because the police was all over the place holding guns it was difficult to run anywhere and the white people got their way but not us so we stood together through every step of the way so our president on that time was [union leader] he was the one who said he has no people standing on the mountains his people are at work so we had no one by our side so we were talking saying we will all die here if these people won’t help us but never did we really believed that we would really be called because we all voted and we knew those apartheid years were long over so they really amazed us we were shocked to see we were really getting killed.

M: Where were you when all of this happened and how did you feel?

P: I was there I went there early from home because it was said [union leader] is bringing us feedback because he never came back the previous day so he came by at 8 o’clock and he said he could not go in there because when he got there they had a meeting than he said he was going to go back because he did not want to stay away and leave us wondering where we are all kinds of police was there then he went off again he then came back around about eleven to twelve and I was there saying to myself hey these people are going to kill us today(laughing)they are going to shoot us I thought to myself the was another friend of mine there I said to him hey lets go here because we heard we cannot be helped so we went off when we got at his shark because there was a shop we both drinks then we heard gun shots we ran up the mountains again we just saw ambulances people were short some people were running in the bushes and the hippos were running was stabbing them with the sharp part of it in the front and was running on top of them that is why a lot of people died people tried running away but could not when you’ve been shot in the leg they came back to finish you we were fortunate because we left before the shooting even started.

M: The people that were on the mountain do you think they were afraid or where they going because they had to?

P: No we were all afraid because we knew a gun kills but we had no choice but to pull through because we were forced to go to work with no explanations and we’ve already not been at work for so many days they were going to treat us even more bad back at work if we have given up so easily so what was the use of losing so many days if no one can give us a straight answer at the end of the day we just had to stand on our grounds

M: Tell me Sir, from the people you were living with even the brothers from your church was there any violent or angry looks on their faces that was caused by what was going on that day?

P: Yes people were very angry when you looked at someone you knew they were different people they were fighters they were very powerful.

M: What do you mean Sir, what were they doing if you still comfortable with that?

P: How can I put this you could just pick up the anger because when one man says people let’s go here then you could hear the majority saying no we are not going anywhere and you could see in their faces this person would kill me now(laughing)

M: We are almost done now but I’d like to know from the news when we were watching this incident there were no women involved there can you tell me why there were none even though they were also working in the mines?

P: They were not forbidden to be part of the strike they did not go because it was far and up the heels they only came when people was killed

M: A man that was a coward do you think he was gone and didn’t make it that day?

P: No a man like that was never going to make it because a coward is afraid of pain and death so no a man like that was never Gonne make it( laughing)but I must say everyone has that afraid side to them because when I saw all of those police I decided to leave

M: So one can say those man was afraid sitting on that mountains?

P: Yes they had that fright in them hence ‘I’m saying never did anyone think that they would really be shot on because the apartheid years were gone and they all voted they thought police are for protecting little did they know police has the right to kill so I’m saying there was fright in them

M: (laughing) So in your minds and hearts for you people that are still leaving here and working in the mines how did this incident left you feeling?

P: It left us very sad and disappointed in our government which is under the [ruling party] no one needed to die on that day if we are going to be led by a government like this it is going to be really bad because what was so difficult to send the deputy precedent just come and speak to the people because he knows how employers speaks to the employees we are [union] which was built by the deputy president what is the deputy president’s name but they failed us as workers it was them who got us killed rather than to give us the 5000.

M: {Deputy president’s name].

P: Yes [deputy president’s name].

M: Bhut [name] I must thank you for giving me all this answers I don’t think it was easy for you to speak to a stranger lime me about something so huge thank you again and I promise like I’ve said before this is not going to affect you in any way it is only for research about the mines and the mine workers like I’ve promised at the beginning I am not going to use your name that is why I have been calling you [name] since there are a lot of [name]’s out there but I promise to cut that name out as well once again thank you very much for your time before I switch this tape of is there anything you would like to add from this conversation we had any last words?

P: No I would just like to thank you for speaking to me although I did not know you but now what I would like to know are there any changes that can take place now because we were not even given that money yet but the way we live here now is better than the way we were living then now we don’t know whether that thing from the mountains is still going on or not?

M: I am not going to lie and make any promises living you having false hopes I am not from the government but I am working for a researching company that does research for the government and report back we are going to make the analyses of the information we got from the people and we will write our reports to the members of the government hoping that the governing members when we write and report this articles they know there are people like you living under these situations and that are not being treated the way they must be and I’m sure that the government when we’ve report this and they see it will also feel it was wrong and it will never happen like that again it was wrong that is all I can say because you people are our elders and we do wish that things get better I can only put it like that because I don’t want to leave you with false hope can we close now Sir?

P: Yes we can close thank you very much.

*The end.*
