# Supplementary material for: “You see this thing is hard… ey, this thing is painful”: The burden of the provider role and construction of masculinities amongst Black male mineworkers in Marikana, South Africa
Source: PLoS One. 2022 May 23;17(5):e0268227. doi: 10.1371/journal.pone.0268227 (PMC9126392; doi:10.1371/journal.pone.0268227)
Supplement: S1 Data — (ZIP) [file pone.0268227.s002.zip › Anonymised Transcripts/INTERVIEW 711_0150_anonymised.docx]

**INTERVIEW: 711_0150**

***CODES:***

***M: MODERATOR, P: PARTICIPANT***

M: Thank you Mr [name] for your time. I have the questions I prepared. I will hold this record like this so that we can be able to hear what we talking about and I will ask that we please speak a little bit loud and clear so that our voices can be heard on the recorder. I will start by asking your age.

P: [age] because I was born in [year].

M: [year]?

P: Yes.

M: Are you married?

P: Yes.

M: Are you traditionally married or civil married?

P: Traditionally.

M: What is your passed last grade at school?

P: Standard 2.

M: Standard 2?

P: Yes.

M: When did you start staying here in Marikana?

P: 2010.

M: Ok. When you started working, where did you work?

P: In [place]by the mountain.

M: Which year?

P: 2006

M: And you’ve been working in the mine since then till now?

P: Yes. From the mountain I went to [place] where I worked with bricks and then came here to contractor. All these I’m talking about are contracts.

M: When you identify yourself where would you fall, under Xhosa, Mpondo or Sotho?

P: Mpondo.

M: Ok. Alright, thank you Mr [name], I got all those details. Now to start with our conversation, please tell me where you are from, where did you grow up, how did you grow up and your whole back ground please. Please feel free. Stress more on your family, how many are you in your family and how did you grow up? Just like that Mr [name].

P: My name is [name], surname is [surname] and clan name is [clan name]. I come from [place]. We are [number] at home, three boys and three girls. I grew up with my father around and I saw no problems, everything was alright. But things changed when they passed away, because my mother passed away in [year] and my father in [year]. I saw no problems when they were still around but when they passed away I saw there was a problem. I started seeing difficulties and had to start looking for a job.

*(Too much back round noise, sounds like machines.)*

*(greeting exchange)*

M: Ok.

P: So I came to look for a job here in the mines up until now, struggling and working in the contractors. Like I said I was never a mine worker. So I’m still working like that.

*(Back round noise interfering with the recorder)*

M: You haven’t seen anything better?

P: No.

M: Alright. Let’s talk about your other brothers as you mentioned that you have two brothers. Where are they and what are they doing?

P: My brothers are working and they started working while our parents were still alive. They were lucky, they started working while parents were still alive. My brother worked here in [place] and the one after him worked at [place]. I’m the last born at home.

M: Oh ok.

P: The older one worked until 2002 and then lost his job, I don’t know how. He then went to work in [place] and he is still working there until now. I don’t know which year he started working there, but he is still there working in the mine. And my other brother, the one that was working at [place] was transferred to [place]. He is still working there.

M: It’s also mine there in [place]?

P: Yes he works in the mine.

M: They are not working on contractors?

P: No they never worked for it.

M: Oh ok. As you were saying this is your house, who do you stay with here in the yard? Who is the person you see as a family here in Marikana?

P: I’m a little bit confused, do you mean the person I go cry to when I have a problem?

M: Yes.

P: Alright let me answer you this way, firstly here in the house I stay with my nephew, my elder sister’s child. I don’t have another person I can say it’s family here.

M: Who do you go to when you have a problem or an emergency?

P: I have my home boys that I go to down there.

M: Ok.

P: I state my problem and they give me what they have if they have something. If they don’t have, then they don’t have.

M: But you support each other? Would you say there’s a support system going on between you and your home boys?

P: Ey! My brother, what I mean is when I go to some with a problem I feel that yah! I’m talking to a caring person here. But other we just greet each other and it ends there. I can’t really say we all support each other. I’m always in the house, I just go visit others when I just feel like chatting. You understand?

M: Ok. I hear you. At home what is it like since the parents are no longer there?

P: It’s very difficult, I don’t want to lie to you. They left their house in a good shape but now eish!

*(Participant’s cellphone rings and he attends to it.)*

P: But now that the owners are gone, the home is no longer in shape, it’s falling apart. I now have my own house.

M: Ok. The reason I was asking about back home is because you told me you are three brothers at home, so as a man what are the things you need to do in order to show that you are a man amongst men?

P: The way I see it, to show you are a man you need to work for your home, improve things, send the kids to school and help your family with the things they need. You must not be a failure. I think that shows you are a man or at least it is how I see it.

M: When you looking Sir, how do you think helps you to manage all those things at home?

P: I want to say back home I have one hut, I managed to buy sheep and keep them even though they not that many. Now I was starting to plan to buy cows but that is still in progress.

M: Ok.

P: As for education, I would really like my children to get to tertiary level but for now they are still young because the first born was born in [year] and the second one in [year]. So I’m still checking, I might get a job and achieve all the things I wish for.

M: When you thinking about all this things, how easy do you think you can manage to achieve them?

P: I don’t see these things difficult at all if God can make a plan for me to get a job. I think I can manage to do them the way I want. I don’t see difficulty there.

M: Difficulty in finding a job, how does that affect you?

P: It affects me a lot. But this is where life has put me, so there is nothing I can do. I will just have to endure up until my time comes and if time hasn’t come, it hasn’t come then.

M: Ok. Now Sir as you mentioned that you have children and I’m sure you play a role in your children’s life. How do you feel now… because of work you might find it difficult to provide everything they need. Does that make you feel like you are a failing father?

P: I see difficulty Sir, I don’t see it easy. Because there are things I wish I could do for them but I don’t manage to do them. Because of money I fail.

M: What kind of things you wish you can do for them but don’t manage to?

P: Firstly, when you have kids there are rituals you need to perform, and those things need money. Sometimes a child wants something, a certain fashion and ask me to buy it but I fail to because I don’t have money.

*(Noise interfering with the recorder.)*

M: How does that make you feel as a man when you fail to provide what your child asks for?

P: I don’t feel good because it’s not right when a child asks something from the father and the father fails to provide. If you can’t give the child at that time, then it’s better if you promising as to when you will buy it.

M: Oh. I hear you Sir. Thank you. You’ve been here for a couple of years now, can you please share your experiences of working in the mines. How do find working in the mines?

P: Working in the mines Sir…firstly, we get on the lift, that’s what we use to go down. As soon as we get off the lift we start walking under rocks, we then get to a room called wet room, where we are reminded about the safety rules. While we still waiting there before talking about rules, the manager walks in front to go and check if things are still as good as yesterday. When he comes back, we get reminded about the rules and then we go together with the supervisor to double-check if things are still as good as yesterday. When we come back, we take our uniforms and we then take a cloth, make it wet and clean the dust off. The we pull the machines and start working. All this is happening underground and it’s not safe there. We just survive through God’s protection, Otherwise when you go to work you don’t know if you will be coming back or not, because the rocks can fall whenever they feel like.

M: I hear you Sir and you are not the first person to tell me that. What makes you people continue to work in a dangerous place like that?

P: What makes me continue enduring this it’s because I have a family at home. That is what makes me continue. So even if I want to give up, I just think of the problems I have at home, so I decide it’s better to just be strong and endure the situation here. Because if I leave here, my children will go hungry, so it’s better to endure. It’s the problems that keep me here.

M: Oh I hear you. Now as a man working in the mines how do you see people viewing you?

P: Firstly, I view people differently, so I don’t notice how they view me.

M: Alright, let me ask it this way, as a man working in the mine, do people respect you or they just look at you as just a man working in the mine?

P: Some people give you the respect you deserve. I’ve noticed one thing nowadays, people respect you according to how much money you have. If you don’t have money, you don’t get any respect.

M: Alright. As for you as a man how do you view yourself as a man working in the mine? I mean in terms of respect from your family and the community.

P: I have no problem with that. I liked myself with others. I respect other people.

M: You mentioned that you like staying in the house when you off sometimes. Let’s please go back on that. There is time for work and time off work, what do you do with your time off work?

P: There is nothing that I like, but other people like soccer and…but I will not include church because it has it’s own time, you see. Otherwise I go to church but it’s not everyday.

M: Ok.

P: So people that love soccer are always in the soccer field, and it’s always full there, that’s where they spend their time. Some people go spend their time in the tavern drinking. As for me if I’m out of the house, I just go visit another man around here or just sleep in the house if I don’t have a visitor.

M: I hear you mentioning that people go to the tavern but I don’t hear you saying you also go there. You don’t go there?

P: No Sir I don’t go to the tavern.

M: Why is that?

P: People have fun there.

M: Can you please explain further about what you mean by that.

P: You see people drink there and have fun but you know things done at night don’t end well. You hear that they were fighting and stabbed each other. And if we seating there and I knock your beer down by mistake and say sorry, you drunk and don’t care about that sorry instead you just take your knife and stab me.

M: So about the tavern, how popular is the thing of men and young men going to have fun in the tavern?

P: I really can’t comment on that because I stay in the house.

M: Alright Sir. I wanted to link that question with the one about women. I’ve spoken to other men and they told me that it happens that though men here have ones at home they also have mistresses here. Do you see that happening much here?

P: I don’t have information about that, so I can’t really say it happens or not.

M: So you don’t see it happening here?

P: No I don’t know about that.

M: Alright I hear you. As you mentioned that you have a wife, I know that in relationships there is times where you don’t get along. Now I want to turn to you, when there’s a problem between you and your wife how do you fix it?

P: *(laughing sarcastically)* I know that happens but we created differently as men. Some when they have an argument they go out to the tavern and drink beers. Another one tell the wife to leave the house and go away. But as for me if there is something I don’t like I talk about it and explain that I don’t like a certain thing that is happening. If the person doesn’t listen I beat the person and then seat down again and talk about it so that we understand each other. I must show the person that I really don’t like this or that. Getting angry and keeping quiet won’t solve the problem.

M: So please explain to me about the way you saying you fix the problem.

P: There is many ways to fix a problem and I don’t think we should go deep into that.

M: Alright. I know you said we should go deep into this and I respect that. I just want to ask you, this method you use to fix the problem, does it work for you and your family?

P: Yes Sir it does because up to today I have never had a problem of a wife that goes to her home because we had an argument. She is my home girl but you will never find her packing and going to her home.

M: Oh she’s your home girl, you married your home girl.

P: Yes.

M: I know you were not here yet in 2012 when the strike happened, that’s another thing I wanted to ask you about. How much do you know about that strike where a lot of people were killed in the mountain?

P: I don’t have much I can tell you because I was not here, I was working there in [place]. I only heard that there was a strike here but I don’t know how it happened and what happened? I will be lying if I say I have details of what happened.

M: You will be lying?

P: Yes because I was not here. The only strike I know about is the one that happened here recently when we stayed at home for five months not working. I didn’t see anything wrong happening, there was not even any singing. We just met at the stadium and then went our separate ways. We went home and others stayed here.

M: What was the strike all about?

P: If I heard right, the discussion was about the money issue.

M: Ok. So you were not on that strike where people got shot?

P: No.

M: Thank you. We are moving on well. Now I want to ask you about here in Marikana, how must you prove here that you are a real man?

P: No, I don’t see a need to prove you are a man here. I think when you are a man you need to stay in your yard and do your house chores. I don’t see a need of bragging about being a man here.

M: What is normally the fault when a man is declared stupid?

P: No Sir I haven’t noticed those things. I seat and chat with people but not much, so I haven’t heard them saying anything about that.

M: And for a man to be popular what would he have done to gain that popularity?

P: I have no idea about that Sir.

M: Ok. Now let’s talk about social clubs, are you a member of any social club?

P: No Sir, I know nothing about social clubs here. I just here they slaughtering a cow there but I have nothing to do about that.

M: I mean the community clubs like stockvel and so on.

P: No I don’t know anything about that. The only thing know is about funeral polices where they help you with the funeral arrangements.

M: Alright. Before we finish Sir and we are about to finish, don’t get tired. You’ve been here for a long time now, have you heard or seen this thing of men dating young men here, how it happens and when it happens?

P: Sir as for me, when I arrived here in 2006 I only hear when people talk about it. I don’t have much information about that because I only heard used that it’s a thing that used to happen sometime back. But now it doesn’t happen anymore.

M: So when you hear, what was the cause of that?

P: I don’t know.

M: Alright I hear you Sir. Now as we are concluding this interview I want to ask about this issue that we hear about, we hear that men hear get themselves mistresses. How much do you know about that? How does it happen? How common is that?

P: That’s a very common thing and it’s everywhere, the whole of Johannesburg. In most of these shacks men are staying with their mistresses. It’s only few men staying alone.

M: Where do they get these women? How does it happen?

P: I think when you are just walking on the street, you meet a lady that you start liking and you state your story, asking where she’s from and what brings her here. If you hear she is coming back from home and staying with her sister and you are staying alone, you then decide to take her if you agree with each other. And you then now have someone to do a few things for you when you come back from work. I think it happens that way because I never stayed with a mistress.

M: Alright. No ,thank you very much for your time Sir. I got the information I needed through our conversation with the three of you today. With I will come back and chat with others. We are now finished, is there anything you would like to say, add or ask concerning our interview?

P: No, thank you.

M: Alright, thank you very much for your time Sir.

*The end.*
