# Supplementary material for: “You see this thing is hard… ey, this thing is painful”: The burden of the provider role and construction of masculinities amongst Black male mineworkers in Marikana, South Africa
Source: PLoS One. 2022 May 23;17(5):e0268227. doi: 10.1371/journal.pone.0268227 (PMC9126392; doi:10.1371/journal.pone.0268227)
Supplement: S1 Data — (ZIP) [file pone.0268227.s002.zip › Anonymised Transcripts/INTERVIEW 711_0151_anoymised.docx]

**INTERVIEW 711-0151**

***Codes: M= Moderator***

***P= Participant***

M: I will put it here so that you can see it. This is the speaker.

P: I can see it.

M: You will speak on it. So bhut’ [name] I have drafted some questions here, as you have seen that I have already interviewed other people.

P: yes.

M: I will ask you these questions they are not so many but at the same time they are not few

P: yes.

M: I want you to be free bhut’ [name]

P: Yes, yes

M: Because I am in your place so be free to say anything.

P: Yes, yes we are just having a conversation.

M: Anything we talk about here, I will not disclose because it is part of my job. We have rules and…

P: And regulations.

M: And regulations that I am working within them

P: Yes, follow.

M: So I am…I am…everything will be kept between the two of us. When we are typing this we will take out our names and it will be just two men that were having a conversation

P: Mhmm.

M: All I am trying to say is that be free. Your life and experiences that you are going to share with me is very important to us, they are important so that we can be able to learn about men’s life and how you came about being here at the mines

P: Hmmm.

M: How did you grow up bhut’ [name] and what kind of a place did you find the mines to be, how are you surviving here in the mines, the ways of survival?

P: Hmmm.

M: What are your challenges bhut’ [name]? And all that, so please feel free bhut’ [name].

P: No, no I am free.

M: Firstly bhut’ [name] ohm…give me a…a…a ohm…your age. How old are you/

P: I was born in [year].

M: [year]?

P: Hmmm.

M: Okay. Are you married? Traditionally or legally?

P: At first I was legally married, this is my second wife - my first wife passed away in September [year].

M: Alright bhut’ [name]. Is the one you are married to now your second wife?

P: Yes it’s my second wife.

M: Are you traditionally married or…?

P: Yes we are traditionally married. We just bought the rings and she is still using her surname.

M: Oh…she’s still using her surname…okay.

P: I still want to sort out somethings. I don’t want to marry someone and she leaves me. My first wife left me with two children (M: oh). The first one finished standard 10 and she got pregnant, when she became pregnant for the second time I said “you are now a woman and I cannot take it anymore because I am a mother and a father to you”. My son was born in [year] and he is at the university now…university and he is doing his last year (M: oh that’s good) at the university in [place] (M: oh the last year) yes he is finishing now. I can now relax (M: laughs), I can now relax.

M: You said your first wife passed away?

P: Yes my first wife passed away in [year].

M: Oh [year] bhut’ [name].

P: Yes, [year].

M: I am very sorry bhut’ [name].

P: This…this one is the second one.

M: She is the second one. When did you marry her?

P: I married her in…in [year]

M: [year].

P: Hmm [year].

M: Oh, okay. What is the highest grade that you have passed?

P: I stopped in standard 4.

M: Oh in standard 4.

P: Hmm. I don’t know anything about the grades

(Someone came in and talked to the participant)

M: Okay bhut’[name], which year did you come here in Marikana?

P: I came here in Marikana in 1993.

M: Here in Marikana?

P: Yes here in Marikana.

M: When did you start working in the mines?

P: I started in 1993 because most of the time I was a taxi driver.

M: Okay, where were the taxis you were driving going to?

P: Going to…[place]

M: [place].

P: Driving a long distance from [place], [place], do you know where [place] in [place] is?

M: in [place]…is it here in…in

P: Here in [place], up there.

M: Oh, okay. And you started working in the mines in 1993?

P: In 1993. Then when I stopped driving for white and black people who were not paying us and not giving any bonuses (M: laughs) and you don’t get any sleep for the whole night. December 1993…that is when I stopped working in the taxi industry and I handed out the keys (P: hmm) in December 1993.

M: And you came to the mines?

P: Yes I came to the mines. You know there is that busy time in December (P: hmm), there is no sleeping time, you are going up and down and you don’t even wash, you wash only the parts that are smelling (P: hmm...hmm). You don’t wash and he (the boss) is sleeping and that time we were earning little money, long distance drives were getting R150.00 and there was no bonus. You will deliver the load and go back again (P: Tjo!). When you come back you give him (the taxi owner) lots of money and I though no it’s the same thing I should stop.

M: How would you describe your race? Would you say you are Xhosa, Mpondo, Mpondomise, Zulu?

P: Oh, okay. I would say according to my language I am Xhosa because there is no Mpondo in the book

M: No, it’s there just read through

P: Mpondo or Mpondomise…because we write Xhosa since it’s not in the book

M: Oh, so you are Mpondomise?

P: Yes, I am Mpondomise, I am Mpondo because I live in Mpondo land. I am Mpondomise but I am Mpondo because I live in Mpondo land (P: you are Mpondo because you live in Mpondo land), yes.

M: Okay, there is no problem let us live it like that. Just as I have said bhut’ [name] I want to understand you livelihood from when you were young (P: ja, from when I was young). Please tell me about you, your livelihood (P: my livelihood), where did you grow up? How was your family? How many were you at home? Just give me that back ground.

P: When I grew up my mother was separated from my father while she was pregnant with me. My father was not a learned person but my mother was, she stopped at standard six but my father did not go to school at all. According to what I have heard is that my father married my mother by force (M: oh, okay). My mother stayed with him but later on she left my father but unfortunately she had already conceived me (M: your father has made her pregnant), he has already made her pregnant. My mother gave birth to me at her home (M: okay), after giving birth to me, they say when I started sitting my mother got married to someone who was a military police (M: okay). I grew up living with my grandmother, when I was six years old (M: okay) my father came to fetch me and he gave the [surname] family a cow to pay for the damages. I then grew up living with my stepmother and my grandmother. I grew up not knowing my mother (M: alright), my mother’s home was not very far (M: Hmmm). I only started knowing my mother’s home in….in 1980 (M: oh, in 1980, you were grown up by that time) yes I was a bit old at that time. I grew up and ran away from school and went to [place] to work. I worked in [place]…worked in [place] and went back home but went back again to [place] to do a driver’s license. I got my driver’s license in 1987 (M: okay bhut’ [name]) and I drove the trucks (M: okay), those that go to the forests and also fetch the sand (M: okay). I drove and drove there, then I stopped and went to drive the taxis (M: okay) I stayed there driving the taxis until I came to the mines (M: you came to the mines). Yes, I am now getting old working in these mines (M: getting old in these mines), yes getting old here. As there was that commotion in 2009 where you had to register your name, so I registered my name and got my money and went home. I am now coming….as I am here now I am coming from the second time, I came back in 2011 (M: in 2011). Yes and the mine accepted me again, I am now prepared (M: okay) to retire while I am here in the mines (M: okay).

M: Thank you very much, you have mentioned that your parents separated while you were still young (P: hmm), when you were not yet born. So do you have any siblings?

P: I was the only child at my mother’s home but when I got to my father’s home I grew up with my aunt’s children (M: okay) and my stepmother passed away without having any children. Here at home there were 4 children and I was the 5^th^ one. I grew up with the children of my stepmother’s brother (M: hmm), there were 2 boys and the older one were girls and my uncle’s child who also grew with us was also a boy.

M: You told me that you ran away from school, tell me what was the reason for that?

P: The reason I ran away from school…I was to tell you so that you also tell your children (M: laughs, okay bhut’ [name]), when you get involved with someone who not studying…when your child gets involved with someone who is not studying know that he run away from school. I grew up smoking weed (M: hmm), but the time I am at school I would be thinking about my friends eish who are not studying and when I look at the place where we smoke weed in then I would see the smoke and think that busy with school here whereas my friends are busy smoking (M: laughs). I told my step mother that I am tired of going to school and she said I must write a letter to my father and tell him that I am tired of school but what she is sure of I will not stop going to school while I am still under her roof. Then I started doing some piece jobs…do some piece jobs on Saturday and when the schools are closed, then I ran away from home to [name] and I stayed there for a year and my father would hear about my where about and he would ask people to tell me to come back and I went back because I am his only child and he wished that I would study and finish but it was not like that (M: mhmm). Yes it was my father’s wish that I would study and finish but it was not like that because I got mixed with people who are not studying (M: okay) if I had friends that were at school I wouldn’t be here, I would be eating my money at home in Eastern Cape.

M: Oh, I hear you bhut’ [name] but if I may ask, as you said you would see others smoking

P: No, it was just an influence by friends at that time because school children were not allowed to smoke, they were caned so if I want to smoke I would go below the garden before going to school smoke, smoke, smoke and smoke there and then get an orange peel or anything to flush away the smell of the smoke in my mouth.

M: Okay I hear you but then you ran away to [name] (P: to [name]), what was your plan?

P: My plan was to find a job (M: okay). You know back then you would go school at a very old age so I saw that I am in standard 3 and people who are of the same age as me are working and they are wearing nice clothes, they are not asking anyone and I have to ask my father for everything (M: hmmm) and my peers are buying things by themselves and I thought to myself I am getting late my peers are working, this standard 4 that I am doing is not doing me any good so I ran to [name]. My peers come back with nice tekkies that they bought for themselves and it showed that I am not working (M: laughs), so I ran away to [name].

M: As your peers were working and you were not, how was it? And how would they look at you? How were you living with them?

P: No it was nice being around them (M: okay) because I used to play soccer and they knew that so and so…there were others also…so they knew that we were good in soccer, so some of them when they are going back to work they would leave their soccer boots to me and tell us to continue playing soccer and never stop (M: oh, okay).

M: So were they not looking down on you?

P: Some of them because people are not the same (M: alright), some would look down on me because I don’t have money and some would not, people would never be the same in the world.

M: How did that make you feel as a young man?

P: I wouldn’t mind (M: okay), yes because I didn’t chosen that life so I didn’t mind because I knew that I get food when I get home so I didn’t mind what other people had. Your wealth is your own and for your family not mine so I used not to mind. It is more like when you tell yourself that you will not mix with people who are not working, that also does not affect me because I know that someday you would ask for a cigarette from me and I would give you because I don’t know the beginning and the end of life. Some people who did not pay attention to me back then, today they are able to ask me for an overall and I would give them even I it’s only the trouser (M: sharp). I am not holding onto something he did long time ago, he has forgotten now and I still remember.

M: Bhut’ [name] you also mentioned your children, your boy and your girl (Participant want to smoke) when you were talking about them you mentioned something about the school. Why is the school important to you when it comes to your children?

P: That I mentioned what?

M: You mentioned that you wanted them to go to school

P: No, there is only one that is at school, the girl got pregnant and I ignored it and took the baby to stay with her step mother then again when she was in standard 10 she got pregnant again so I told her that I cannot take it anymore because it clearly shows that when she got pregnant the first time it was not a mistake, it was intentional so she better stop going to school (M: hmmm). I will continue paying for my last born who was in standard 7 at that time (M: alright) as I said that he is doing his 4^th^ year, it is his last year now. I have not stopped carrying for my girl also because I told her step mother that she must tell her to register for the grant money for her children. I also told her that her responsibility will be to buy food for her children and if they are sick she must inform me so that I can give her money to take them to the doctor (M: hmmm). Money for food and other things are not my responsibility.

M: How much is important for you to take you boy to school?

P: It is very important because I still trust him although people are not trustworthy. The reason I trust him is that he still listens to me (M: hmm) number 2 he does not smoke nor drink (M: okay) those are the things he can do when he has his own money. I know that he does not drink and smoke but when he gets a job maybe he will tell me that he will not stay at the locations because of his work or he want to buy a house in town and when I ask what about the house in the location he will start saying I bought it already or say my company bought it for me. I am also giving him a bright future so that he will not end up like me and work in the mines (M: work in the mines), yes he must not end up like me and work in the mines.

M: Why don’t you want him to end up like you?

P: Sorry?

M: Why wouldn’t you like him to come and work here?

P: Because of the way we work underground.

M: Please explain it to me

P: The conditions that we work under are very difficult because of job scarcity so it would be better if he is working there because he is still looking for a job in the field that he studied for (M: okay). It is not easy working here (M: okay) for example you would maybe come here next week to ask for bab’ u[name] and they would tell you that he has passed away, the mine collapsed on him, you get me (M: hmm). Who knew I would be like this? Because I went to work with nothing and came back with an injury (M: okay). Working in the mines is not nice, no tata it is not nice at all even when I stopped in 2009 and took all my money I thought that I was going to get something back at home. When I got home I bought a bakkie (van) but I saw that the money that I am making is not enough for me to take my children to school and it constantly wants to be fixed. When someone dies in the family they will look at you and that person will be waiting at the mortuary for a week so that is why I took my child to school. I told him if he still want to go to school then he can continue and stop where he wants to stop. I told him that “I know that I get little money from the mines but I will try my best and do not compare yourself with other students that are driving their own cars (M: okay) don’t look at them; you must think about where you are coming from and also know that as I am giving you the money, I also have to support back at home so you must keep those two things in mind”. Do you get me?

M: Yes, I hear you bhut’ [name], you mentioned about your injury. Did you get injured at work now that you have bandage?

P: Yes, I got hit by the stones (M: okay) just a small stone but when a stone hits you it straight to another stone, there is no sand inside there it’s just stones.

M: As you said it is not nice to work in the mine and that it’s dangerous why do you continue working in such a dangerous place?

P: It is because there is no other way I can survive because I worked there from 1993 and in March 2009 I stopped (M: oh, okay), when I got home I bought a van and I worked with this van but I saw that I will not be able to take my children to school with the money I get from this van because if something huge happens to your car you will have to fix it with the money it makes and sometimes you have to use your own money. Secondly, you can never do groceries with that money. Thirdly it can happen that someone within the family dies and the car also need to be repaired what will you do? You know when you have a car in the location…I have a private car and a van …they think that you have money what they don’t know is that you have debts (Laughter).

M: Bhut’ [name] as we are talking you mentioned something like…for example you talked about death…what I want to know as you are a grown man what are the important things that you are supposed to be doing as a man here in Marikana and back at home?

P: Here in Marikana it is just a place of work, where I am supposed to do something is at home. I am working here so that they will not suffer back at home because if I am not working I will have to plough in the garden so that we can eat, not only that you still have to build your house so that when I see you in [place] and you need a place to sleep I can be able to help you and give you a place to sleep, then in the morning I will take you to the station so that you can hike (M: okay). You are also building a home for your children so that they will not suffer because my father and stepmother passed away it’s only my mother that is still alive but as I told you that she got married and has her own house but she is still my mother and I also do go to her house they don’t have a problem or discriminate me.

M: Why is it important for a man to do all these things you have mentioned?

P: Which ones?

M: That a man has to build his home. Why do you think it’s important for a man to do all these things?

P: It is very important for a man to build his home because there will be a time where you will not be working or a time where you will be sick. It is not good to be sick in your relative’s home whereas you were working before. Being sick in other people’s places is not good because you will depend to them for everything whereas when I am in my house my wife would wash me before the visitors come, even when you look at me you will see that I am clean in my own house and she will make sure that I am alright in my own house. I am also building a home for my children so that they do not go and stay with the relatives whereas I was working and now they are suffering, where they will also say “we are suffering because of our father”.

M: How easy is it for you as a man to do all these tasks?

P: When you are working as a man it is important to save money for example if you are getting R2000.00 take R500.00 and keep it in the bank, then take R500.00 and…and keep it with you, then take maybe R600.00 home so that they can eat. You must share your money but make sure that you have some money that you have put in the bank that is how you should budget. It is more like when you want to buy a car maybe next year you need to budget. You can get anything you want when you are working but if you are not working you will never get it instead you can become a thug. If you put your heart on something or maybe say I want the car that so and so is driving, when you are budgeting for it you will get it. You can do whatever you want at the end.

M: As you have mentioned that you have a home and you are renting here

P: yes I am renting here, I pay R350.00, so I wake up from here as you can see that there is nothing, I wake up and go to work. When I am on leave I go home I don’t stay here. I am not a Tswana I am from the Eastern Cape.

M: If a man is not doing all these important things that you have mentioned, how do you look at him?

P: I don’t see that man as a man because you can lose your job anytime, I would say he does not think about the future. If this job can end tomorrow what will he do? So he does not think about the future and these days jobs are scarce. If you have not built your home…to us we had to start everything from the beginning because what was important to our fathers was the cows so if you have not bought any cows you will be regarded as someone who is lazy (Laughs) so you had to buy the cows or you are not a real man if you don’t buy the cows.

M: I hear you very well bhut’ [name] but you told me that you are a hustler, how difficult is it to hustle? I just want to hear you experiences.

P: For one to have things it is difficult but not as difficult, as I have mentioned earlier that if you want something with all your heart you will get it unless you are not working but if you are working you will get what you want because you are working. If you don’t get what you want then it means you don’t want it. Did you hear me when I said I have a child that is at the university? Every month I have to pop out R5000.00 and remember that I still have to eat, back at home they want money and I also have instalments that I need to pay. I hustle because I want my child to be at school, for those at home to eat and I also have to eat I can’t work on an empty stomach, you see.

M: How do you feel as a man that can be able to do all those things at home?

P: It makes me happy and gives me strength for example next year I have planned to build a five room house, I have already bought the brick, roofing, parallel line and rafter are at home what is missing is the cement and the money to pay the builder. I have already budgeted for all that now in April for April next year. I bought all that stuff last year but because of some problems…when I start building I don’t want to hear that something is finished and we have to stop building. As I have said that the blocks, zinc, rafters and parallel line, nails…there’s only one load of sand and I still have to organize for the second load, then buy the cement and have money for the builder and then we can start building the house. I want to give the builder all his money and he start building then I will know that what is left is to do the plastering. You have to pan everything and not imitate other people because we do not have the same amount of money and our problems are not the same.

M: How does it make you out stand form other man there at Flagstaff the fact that you can do all these things?

P: It makes me happy as it is his last year now; it seems as if it is not coming to an end for me to be free. When I am counting these 4 years for example in his first year I paid R24000.00 and when I am sitting and calculating all these monies popping out R5000.00 every month where would I be now. Maybe I would be elsewhere because I am a man that has visions and one of my visions would have come through by now.

M: How do other man and the community look at you in your area as you have achieved all these things?

P: No, no I wouldn’t say they are looking at me otherwise because we do sit and talk to each other but you will never know what they are thinking in their hearts. I know that when my son is working they will be jealous because we will both be working although they will not tell me but I know they will be jealous especially if God can be on his side and he gets a job in a government department because I don’t know what he is studying. I also tell him when we are going somewhere that they will not do anything to him because they will say all that you have are your father’s things. I love going with him wherever I am going but the time I was driving a van he would not want to go with me because he knew that we have to load some things (Laughter) he would ignore me and I would leave him alone. I would show him how to get money when the schools are closed in June…I was not affiliated to work daily, people would hire my van for special loads because the van does not enter in some other roads one had to carry a cement in his shoulders so he worked for one half day (laughter) and said I have to go home because they said they want to send me somewhere, then I gave him R50.00 to buy food and then hike home or I would ask the other cars to take him home and he said “I will take a hike” (laughter) and I knew that he has had enough.

M: I hear you well bhut’ [name] and I am happy and I thank you that you are free to talk as you said in the beginning. What I would like to know is your experience in working in the mines, how was your experience if you can just summarize it.

P: Working in the mine?

M: Yes, working in the mine as a man.

P: As a man working in the mine you must save money because there will be a time where they will say the mine is closed (M: mmm), you must save. As a man you must be able to say this year I am planning to do something (M: mmm) if nothing came up then you will be able to do what you wanted to do because as you are planning you are also saving some money, keep on saving and you will get what you want. My cars are old models I have [year] corolla and ford coria can you see that I have old models (M: hmm) and you are driving a polo or a VW and I want that VW but I don’t want to have an instalment, to at least drive that polo or VW I should save money and buy a second hand, a R100 000, R80 000 or R90 000 second hand will still be fresh at least for me to have a car that has a name but for me the cars that I have are enough for me. I am afraid of these new cars but when I am stack on the road with my corolla you would come and be able to fix it but when I am driving these new models I will have to take it back to the garage with a breakdown (M: laughs).

M: thank you bhut’ [name] let me get straight to the point. Does it give you dignity to work here in the mines both here in Marikana and back at home?

P: It does give me some dignity here at Marikana because most of us here are mine workers. At home it also gives me some dignity but I get undermined by my peers that completed the school but that does not bother me because I don’t ask for food from them or borrow money from them. If I have a problem that needs money I borrow from other mine workers (M: okay) eh.

M: Why have you calculated it that way? Why are you comfortable borrowing from other miners?

P: I am trying to avoid a situation where you would talk about me and say look at this join (mine worker) but the one we are working with at the mines for example if I go home or a week and be going back the following week and you have arrived this week and I tell you that I don’t have money to go back borrow me some money and you will get it in such and such a time you will just give me. Unlike that other one who will be going around saying this join (mine worker) was busy drinking beers thinking that he is clever (Laughter) only to find out that he is dome. My being dome is fine because I have children, a wife and a house, you on the other side you are educated and have too minds the educated one and the natural one and I have only one mind because I am not educated (M: laughs).

M: I hear you bhut’ [name] but I would ask you again the same question but in a different way. If a man is not working here at Marikana how would the community look at him both here in Marikana and back at home?

P: You see when someone is not working but has left home saying he is coming to look for a job and then does not find it but continue to stay here because he is still looking, what makes him not to have dignity is when he is not containing himself well that is number 1, do you hear me (M: hmm). Number 2 is when I am not looking after you and you suffer, you must look after that person by buying some food so that when you get back from work he has already cooked. But when you are off you must also cook because he is not your wife in that way you are giving him some dignity. Sometimes you take someone but when he gets here he goes to the sheeben to drink and sometimes you give him R200 to buy some stuff for himself maybe a t-shirt or a trouser and then he comes back and show you then you give him a pocket money of R100 (M: okay). So it differs there because people’s hearts are not the same, some are just coming to see Johannesburg.

M: You said something about cooking, you are a cook?

P: Yes, yes it is difficult when someone is staying with me and looking for work, when I am not at work, you should wash the pots and cook even when I am older than you I should give you a break even when you are cooking nicely, I should say help me with this and that and I should be able to copy from you the way you are cooking so that I can also cook and he can just take the plate and dish up, not that when I am here I am just sleeping and waiting for you to cook as if you are my wife. When you get hired…there are many that I have helped…when you get hired you can continue staying here but month end you must go look for a place to stay (M: okay) so that you can eat what you like, you must go away from me and go cook what you like for example you would cook pap and I like rice and this one is cooking some samp so stay away from me and get your own place. I would also be able to borrow R100 and I would give you back month end.

M: I like when you mentioned something about cooking, I can see that you are an old man, you have a house, children and a wife and you are staying here paying renting. It makes me curious that as a man of your age you are able to cook for yourself. How does it make you feel as a man who has a wife and has to cook for himself?

P: It makes me feel good because this stomach is mine, I must eat and then go to work the following day for example if my wife has been here for 3 weeks she would never say she saw another woman here or heard that I have a woman here, I cook for myself. If I have cooked today I will not cook tomorrow and I will cook the day after tomorrow (M: okay) I will take the food and put it in the fridge (M: hmm) when I am going to work and when I come back I will warm it up and eat then wash my pots. After work I cook again and not cook the following day. Not that I would meet someone and say come and cook for me, no I must only go to that person to sleep with only and nothing else

M: The reason why I asked this is because we have been groomed differently in our homes as man, we know that cooking is for women (P: yes) so how do you do it?

P: As I have said before that I grew up with my step mother and my grandmother. My grandmother loved me very much and I loved her too and I think if she was still alive I would have finished my studies (M: okay). She would show me how to cook and I know how to cook everything, she would show me how to cook pap and she would say pap should not stick in the spoon that you are steering with. So I would go with my grandmother to take some vegetables from the garden and we would come back with some wood. Even when I was in [name] I had no problem with cooking when I was working in the firms.

M: How are the other men looking at you when they hear that you are cooking for yourself?

P: As I am cooking for myself…we are at the mines here so most man cook for themselves. At times I would say to someone come over so that we can have a meal or maybe we are rushing to go and drink but I would cook first and then we can go because I know that some other day he will cook (Laughs) yes, yes.

M: There is another question that I wish to ask but before we go to it can I ask…each man or as man which I am also I know that there are goals that you have for your life, you want this to happen in your life, where you would be able to say…when you have retired…you have done this in your life. What kind of man would you like to be in your life, what kind of a man do you picture yourself as when you are about to retire?

P: About what?

M: About your goals where do you want to see yourself?

P: I don’t want to suffer when I have retired, I want to be able to buy myself anything I want and not depend on the grant money. I should be able to have my own money from the mines and not depend on the grant money alone that is my goal. Even if I die there should be some money that I will leave behind for my children and my wife as I have said that this is my second wife, so I should not leave my children suffering even the one that I trust maybe when he gets a wife we will start fighting.

M: I hear you bab’ u[name]. How far do you think you are with your goals?

P: Eh, start from [year] if I am not mistaken I am now left with 15 years (M: okay) before I retire here at the mines, it’s only 15 years that is left if I am not mistaken.

M: How far are you with reaching your goal?

P: To reach my goal is far as I have said I am trying to build my house and I am paying for my child’s fees (Someone came in and interrupted but they went out again) as I have said that I am focusing on building my house next year because I know that I would not be paying any fees so that is when I will see my direction.

M: As man we cannot stay alone for a long time so I would like to know if it is happening a lot here in the mines and what is causing it to happen?

P: It happens when you love sex, I came here in 1993 and the year would end without me having desire to have sex and I would only have sex when I go home but now the cats are staying with the mouse, these day we are staying amongst woman so in that way you have to control yourself as a man, if it happens that you sleep with a woman it must be just sex and nothing more and you can give her R100 when you see her but if she wants more than what we have agreed upon then we break up because if you let her rule you, your plans will not come through. As you are earning R8000 maybe this woman you are involved with has her own children so with the money you are getting…as I have said I have to give my son R5000 and also make sure that they have food back home…this other women will also want money from me to feed her children but if I am giving her just R50 or R100 that is enough. You must not be greedy when you are involved with another woman, you must not always call her to come, be scarce so that she can say you are scarce then you can say you will create a chance for her to come even so she must not come to your place, you have to go to her place because I wake up at 4h00 here and go to work so if she is here she will want to stay behind and sleep and when you return from work she has washed all the dishes, your clothes and cleaned the house too and that will cause a problem between you and your wife because people are watching and they will inform your wife via a cell phone or a letter.

M: As you have mentioned that you are staying amongst woman here, what is it that you think woman want to gain?

P: They are also looking for jobs in us, some have houses that are bigger than ours because they have succeeded getting money from us.

M: How regular is it from you as you have said it has been a long time since you got here?

P: When I first got here in Marikana I did all those things a lot but then I saw that I am not achieving anything from doing that so I decided to stop and it was just for a short while because when I got here I was staying in the Hostel so we would go and drink outside the hostel and then come back with woman and that is where you lose everything. So I decided to stop because I am not gaining anything from doing what I am doing. I was good in soccer so I got a job through playing soccer so I would go with my team to play and then get woman with them but when I stopped doing all that I stopped being friends with them but I did not stop playing soccer.

M: Would you say this thing of man getting woman is popular here?

P: I would say yes it is very popular even old man they are doing it, it is very, very popular. Single woman do get man here and you will be lucky if you could find a man staying alone here.

M: Where do you get these woman?

P: Around here, they are staying amongst us, so we get them from here. Some man take woman from back home.

M: We can continue now as I have asked you before to tell me about your experience in the 2012 strike and I said wait a little I first want to ask you some few questions. Can you now tell me about it, your experience and what you have seen from it?

P: We were forbidding people from going to work. If I am not mistaken it started around the 4^th^ or 3^rd^. Machine boys were the ones that were striking and I was working a night shift and I was not a machine boy. The following day they said all workers should go on a strike and we did. We were staying on that mountain, can you see it? (M: yes I can see it). Do you know [place]? (M: not exactly), there is mine that is on the other side of Marikana which is known as [place] and I was working on it. On the first day…we were represented by a union called [name of the union]…when we were going to our managers there were securities at the gates and they started firing the guns and we ran away. They also surrounded the place that we stay in so we decide to go to the mountain because they cannot do anything to us when we are staying on the mountain. Some were going to work so when we went to the 3 shaft we saw policeman and soldiers so we went to talk to them, five man would go and talk to them to tell them that we are not fighting we just want to pass so that we can talk with our managers, but we were also carrying our weapons because we saw that we were being attached. The head of the police said we should live the weapons behind and we said we will go and leave them in the mountain so he asked if he can accompany us to the mountain would we give him the weapons and we said, yes. There were only old people that were left in the mountain at that time. The police just said he will count to ten and if we do not drop the weapons they will shoot us and they started counting. When they reached ten they did not do anything but when we turned around the helicopters were above us and the police were surrounding us and they started shooting and we started using our weapons and 4 people died. The police that were killing us were those that were in the helicopter because those that were on the ground they ran away because people were now trying to take the guns from the police and the date was the 12^th^. On the following day we were going to [place] we went through the grounds but the police did not allow us and we forced our way through. When we reached the rank they were many of them and they talked to us and we answered all there questions and then we went back to the mountain. On the 16^th^ on Thursday there was every kind of a police man that you can think of and soldiers that is when they killed 34 people. They were riding on the hippos shooting everyone some were injured some were not and that’s where most people died and we ran away to the shacks until president [name] came to look for those that were hospitalized and he did not come to ask us what was the problem. Since then I don’t want anything to do with [ruling party] because other parties came to ask us what is the problem and we told them that we are not fighting with anyone but the police just come and shoot us.

M: Thank you very much for sharing, but how did you manage to survive the strike and not get injured as you told me that you were involved in everything?

P: I also don’t know all I can say you have to run away in order to survive but I would also say it is because of the ancestors. There were soldiers that were shooting from horse but the ones that shot us more were those that were in a helicopter and in the hippo grinding people so I don’t know how I survived. Running away save me I must just say.

M: We also watched what was happening and we were scared but what made you to stand your grounds whereas you can foresee that this will not be good?

P: It is because when the police started shooting we would sit down and they will shoot and shoot but we will be sitting down so that we can find a way to run away. The boer would say shoot and someone form us would tell us to sit down and they would shoot and shoot until its dark and the boer would say stop and we would get up and at the same time they would be going back. We did that until the way was cleared and we could run away that’s how we survived.

M: Would you define the man that were there on the mountain during the strike as brave?

P: No, they were not brave because we were fighting for our rights, we were fighting for our rights. It is very painful to work underground for just R4000…R4000…R4000 and in this R4000 you are supporting, taking your children to school and you also have to eat, so I wouldn’t call it bravery but fighting for your rights, so you must die for your rights.

M: I want to know how were you feeling the time you were there, were you brave or afraid?

P: We were brave because we were fighting for our rights, we were really brave. We even said even when they can bomb us it does not matter because we are fighting for our rights because it was not nice to work for little money and not be able to build your home whereas you are working. The neighbors would mock us and say look at him he is working but there is no sign not knowing how much I am earning.

M: Have you noticed any change from other people maybe say you know that this man is a forgiving man but after the strike they have changed and became bitter?

P: Yes, there was a change after we were given the money.

M: I am asking whether were there any changes before the 16^th^ of August when you were still in the strike where you find that man are ready for anything?

P: Yes, when you are going to a war you are expecting anything, life or death. You don’t even get time to wipe your nose when you are fighting because we were not fighting anyone but our rights. We wanted the government (police) to accompany us to the CEO to talk to him and they said you are not going to talk to anyone and we said it is fine we will stay on the mountain until we get answered.

M: Were you not aware of the danger that the police were putting you in when they surrounded you?

P: We were aware because they surrounded us with the razor wire and we had to go over it and they strayed us with hot water where it would live yellow or blue marks on your back. They were killing us, I know that the [ruling party] solved the problem but what they did to us…they were really killing us. If I can show you the video you will also see that we were fighting for our rights and running away helped us, those that did not run away they died. We were running away with our weapons because they were spraying us with water and on the other hand shooting us.

M: How is all that affecting you mentally?

P: It left me...in a way that there were sounds of guns in my ears, it was Friday, Saturday, Sunday, Monday and on Tuesday I went home and on Sunday I came back until the matter was resolved. Those who were there would tell you that there were gun sounds in their ears. They were using AK 47 were it would just be dark when it is used like its August where there is dust because they were shooting at the same time you would hear khakhakhakhaaa (sound of a gun) hey it was bad (M: what you are telling me is painful). Have you seen the CD?

M: No I have not seen it, I will look for it.

P: You can get it from the boys that are selling on the streets around here. Just tell them that you want a Marikana CD they will give you it’s only R10. You will see it yourself if you can watch it.

M: Yes it was a very sad thing I remember it well. Let me thank you for your time and for opening up to me thank you very much. Do you have anything that you want to add on top of what we have talked about?

P: No, no I am fine now.

M: Oh, thank you very much. As your wife is here we can go back so that I can explain if you would like us to do that.

P: Eh…no she can hear it but you can leave the paper behind so she can read it by herself because she is educated.

M: Okay, there is no problem I will do as you wish (P: yes, so that you continue with your work). Thank you very much I wanted to make sure that she also understands that I did not force you to talk to me. You have a right not to sign that form as I have mentioned before so I will just explain it to your wife and she can just call me if there is anything you are not clear about. Thank you very much.
