# Supplementary material for: “You see this thing is hard… ey, this thing is painful”: The burden of the provider role and construction of masculinities amongst Black male mineworkers in Marikana, South Africa
Source: PLoS One. 2022 May 23;17(5):e0268227. doi: 10.1371/journal.pone.0268227 (PMC9126392; doi:10.1371/journal.pone.0268227)
Supplement: S1 Data — (ZIP) [file pone.0268227.s002.zip › Anonymised Transcripts/INTERVIEW 711_0152_anoymised.docx]

**INTERVIEW 7110152**

**Codes**

***M = Moderator***

***P= Participant***

**M:** Okay Sir I will put this Dictaphone next to us so that it will be able to pick up all our conversation however we must speak loud just to ensure that it picks all the information. Let me thank you for giving me your time. I really appreciate your effort even coming here to seat with me and have this conversation. I would like to welcome you please feel free as we are about to start with our conversation, please answer the questions as much as you can.

**P**: Yes, however I will not answer questions that I do not know (Laughs)

**M**: Exactly (laughs)

**M**: Please can you provide me with your age, which year were you born?

**P**: I was born in [year].

**M**: Are you married?

**P**: Yes, I am married.

**M**: Are you married traditionally or legally?

**P**: I am married traditionally.

**M**: What is your highest qualification or highest grade you passed?

**P**: I passed grade 7 it was then called standard 5.

**M**: When did you arrive in Marikana?

**P**: I only arrived here in [year], I was working in [place] before.

**M**: When did you started working in mines?

**P**: I started in [year], which was my first time. I never worked anywhere else I came straight to mines after I dropped school.

**M**: You were very young I must say.

**P**: I think I was 20 years of age then, you can count.

**M**: In [year] you were 10 years of age; you cannot be 20 in [year].

**P**: I am not sure but I know it was after the independence of Mathanzima *(Kaiser Daliwonga Mathanzima was a Leader of Abathembu in the Transkei who’s indepence was given in 1975)*

**M**: The Independence of Mathanzima was in 1976 right?

**P**: Yes, the independency was in 1976 it was just after that, but I know I started working in mines between the ages of 19-20 year of age. Taxies were very scarce then we were commuting by trains.

**M**: You have been working for quite some time isn’t it so? What tribe or ethnicity do you belong to, would you say you are Xhosa, Sotho, Tswana etc.

**P**: I am Xhosa

**M**: The sun is very hot this side can I move away. It’s much better here thanks, alright! First and far most please understand that I have these questions that I prepared in my hand so that I remember the questions that I am supposed to ask as per my work requirements. Please feel free and speak openly this conversation will just flow on its own but guided by these questions. Please tell me about yourself where you grew up, about your family and how you grew up, briefly.

**P:** So you want me to tell you about the past (Laughs)

**M**: Yes, please (Laughs)

**P**: I grew up as a kid attending school, I will start with that point. There were no pre-schools back then, nothing. Kids were starting from school from Sub-A, and they would go and come back from school with other kids. They taught as well at Sub-A, passed to Sub- B all the time they taught us how to count eggs sometime with goat dung we were not using calculators. For our lunch boxes we were dished boiled corn made from mealies (Inkobe). Back then there was no catering at school, there were lot of thing that we were taught. We were instructed to make brooms and it was called handwork. I am narrating my childhood are we together?

**M**: Yes, I am listening.

**P**: After school we were sent to fetch livestock and if you do not bring the livestock home fathers were very strict they would beat us (punish) and sometime we would end up sleeping in the kraal and moms would snip and bring food for us. We grew up playing a lot, soccer was not popular the only popular game back then was stick fighting (Intonga)and we would fight amongst each other as boys. No one would go and tell parents if they are beaten up, instead fight. If one has a wound, we never went to hospital even if we are injured.

**M**: Even if you are badly injured?

**P**: Yes. We used to the put soil dug by mole on the wound and it would heal.

**M**: Is that so! So how many siblings do you have?

**P**: There were seven of us, five boys and 2 girls.

**M**: Are you the eldest?

**P**: I am a [number] boy but a [number] born, I have [number] eldest brothers, one older sister, myself being a [number] born, two boys after myself then my younger sister.

**M**: Are they all alive?

**P**: No three of them died, there are just four of us left behind and we all have our own houses.

**M**: What about your parents?

**P**: My father was born in [year] and only died in 2012.

**M**: Your father died very old!

**P**: Yes, but my mother died long time ago.

**M**: Do you have any relatives here in Marikana?

**P**: Unfortunately, I do not have any relatives here in Marikana. I am just renting for myself here.

**M**: Do you have any people that help you in times of difficulty?

**P**: Are you referring to my home boys?

**M**: Yes, any people that assist you whenever you need help.

**P**: Yes, there are people that I normally run to whenever I need assistance mostly financial assistance as you know things are not free at all nowadays, so I do have people that I normally run to when I need help whether I need maize meal or hundred rands.

**M**: So what kind of people are those, is it people you met here?

**P**: Yes, some and some are people that I met in my previous employment.

**M**: So here in Marikana do you stay alone?

**P**: Yes, I do not live with anyone not even a partner.

**M**: I’ve heard on previous conversation I had earlier that it is common for men to stay with partners that they met here In Marikana. How true is that?

**P**: Yes, but it happens with people who love such things, it happens more especially to people who needs shelter and they end up being offered a roof especially women. There is a lot of robbery here.

**M**: So why do you stay alone if such things happen?

**P**: No I am not that kind of a person, I wouldn’t get myself into unnecessary debts because women will be telling you that they mothers are sick or died and you’ll have to go make debts for them, no I can’t do that I can’t have debts that aren’t my own.

**M**: So would you tell me why you do not want to stay with someone, I heard you mentioning few reasons can you give me more reasons why you would never stay with someone Please explain further?

**P**: It’s like this, I do not know how to put it. I’ve seen it, it’s like creating an opportunity for some else to be able to go anywhere they like and do anything they love (Cheating) yet have a place to stay and the money they get they not going to show you, you’ll go and make debts yet they have the money they get from their secrete lovers. Even when you make groceries if you stay alone you’ll buy 60 eggs if you break two eggs, eat and go to work how many eggs will you have left?

**M**: 58.

**P**: Correct but let’s say you stay with a woman she is going to cook for both of you she prepares four eggs you eat, go to work how many eggs do you have left?

**M**: 56.

**P**: Yes, and then let’s say her friends come and visit her while you sweating underground and they make some eggs how many eggs will you have left when you come back? (Laughs)

**M**: (Laughs), so you took a decision that you’ll never stay with a women you meet here?

**P**: Yes, I would rather call my wife to come here or else go to her, but then it’s like that your wife will save for you they can send a child to a shop to buy food for yourself not these ones they will use all your money.

**M**: I hear you, so are you telling me that even thou this occurrence is common you do not do it and even thou lot of other men are doing it?

**P**: There you go! You starting inviting the devil (laughs) what I cannot cope with is staying with them (women) under the same roof. You see in the pubs we do meet people and the ones you get there will not take your cellphone number and they will not going to want to stay with you but it will be just one-night thing. So even when your wife comes they will not come here and disrespect her. Sometime we lie and tell them that we do not have wives and then when your wife comes they will ask who are these people and they will not want to go because they have their personal belongings in your house.

**M**: What you, you say are the intentions of these other women who stay with these men here?

**P**: Well I can’t say what their intentions are but what I see are people who are breaking other people’s marriages, because these men when they taking their leave they will have to divide their salaries and therefore by so doing they are not supporting their children. Otherwise you’ll be taking home a very small salary and you find that you wasting money feeding someone for 5 -10 years whom you do not even have a child with just a liability.

**M**: So what do they earn on these relationships with these man according to your own views?

**P**: Nothing but they are abusing these guys because these men are losing their marriages, you then see men who do not want to go home when we close work because they are divorced by their wives.

**M**: You told me that you are renting here, tell me do you have your own family back at home?

**P**: Yes I do.

**M**: As you said you are married can you tell me more about your marriage, your wife and kids?

**P**: I’ve got kids

**M**: How many kids in total?

**P**: I’ve got seven sons and two daughter.

**M**: Is it, wow! how old are they?

**P**: They are old two boys are married, other two boys are working others are in school and one girl has been married.

**M**: Now that you have a family and you are a grown man. What are the important things that you think you must do as a man in your home?

**P**: It is to build that home ensure that I provide for them, unite my family even when we have a cultural ritual be able to unite them and they should be able to respond when I need their assistance that is how I raised them. I have been always there for them and therefore they should be able to response when I need help. Even thou the other sons is mostly persuaded by his wife to build a house in town but he must just not forget us wherever he is.

**M**: You mention that it is important to build a home, why do you say it is important to build a home?

**P**: Home is like an umbrella, even if your kids houses can burn to ashes or if they fight with their spouses they are able to come back home. It’s like if all these shacks burn we can go and sleep in that hall while the shacks are being fixed. Even if you decide to go away from your home for years but once you decide to come back I’ll find home where it is.

**M**: Now that you have been working for quit sometime how would you feel if you fail to build your home?

**P**: Very sad, because I it would mean that I have used money recklessly or I would say I am bewitched even thou I have never seen devil or witch but when things go wrong we turn to blame the devil but not facing the reality that some things just couldn’t go well

**M**: How difficult it is as man working here in mines to achieve all the things you want to achieve, what are the challenges that you face as a man to achieve all that you want to achieve?

**P**: Before having challenges one need to plan, even if you want a car you can get it, but you need to prioritize do not want to achieve everything at the same time. It’s difficult sometimes when you do not have any one who shares the same vision with you example; when you send let’s say R4000 to your wife to help you buy material for building let’s say if they do not see things the same way as you they will end up doing something else with that money or even send the money to their family. So it’s important to have someone who will be on the same understanding with you. Those are the little things that contribute to greatness. It’s the same as planting spinach if there is no one who is going to look after it will not grow properly.

**M**: So since you are working here in Marikana, Lonmin, how does that makes people perceive you both here and back at home?

**P**: It makes me feel respected, more especially when working well with people respecting them and not having any kind of problems with people. One should speak well with people so that they respect them. Even kids when you have respect they will respect you. Back at home people respect you because you have respect, your kids have respect and because you livestock.

**M**: The men that doesn’t get respect here in Marikana and back at home are people that do what?

**P**: Those are the man that doesn’t have wives, those are the people that dance the whole night when they get paid, and they are the man that do not send money back home. They are only respected by the ladies at the Pubs but are told by us that they are just making money but not working because when a person is making money they just use their money recklessly but a working man buys tangible things with his money. How do you see it?

**P**: I am listening well I can say I am learning from your views. Tell me what are the thing that you can count that makes you feel you are the man that you are today because of them?

**P**: I would say I grew up obeying my parent’s restraints, I worked, I took a wife, I built my house and I educated my children and I had my livestock and garden. You must have your own things so that you do not buy every time.

**M**: You mention passingly that these days women must not be beaten, since you are a grown and have experience, how do you discipline your wife?

**P**: I call my parent I explain the situation to them, for instance if I gave my wife money for our house and she uses it on something else then I explain to my parents then they will at least speak with her that’s the reason I report her to them. I would never beat my wife because I would never want to see myself behind bars and if she takes me to police when I advise her then I will rather take my jacket and go somewhere else. Just like kids that do not want to obey their parent’s disciplines then they grew up corrupted and spoilt because there’s no one who advises and discipline them. Today’s kids are over protected by government and therefore they are no longer our kids but government’s.

**M**: If you have a grievance with another man in your work place or here where you rent how do you solve the issue between you?

**P**: I normally call other workers and tell them what I did not like, please speak with him tell them what you did not like so that they address the matter with that particular person.

**M**: I hear what you are saying and I understand its importance but when do you show the person who have wronged your strength or power?

**P**: My brother you do not just show other people your strength or power up until they start fighting. When you fight its either you are defeated, you run or you die so fighting is painful there is no turning point in fighting. In the olden days we used to fight and it was not ideal to report who ever beats you, but you just had to fight. Nowadays there is no respect even a young man can disrespect you and once you decline them you’ll be questioned.

**M**: Thank for your views, I understand you have been around here for quite some time please tell me what do you do when you want to relax your mind on your free time?

**P**: My brother I will be honest with you I do drink, on my spare time I go out and buy my drinks gets to the pub drink few bears, sometimes I go out and watch soccer even on TV sometimes or I watch movies. That’s how I relax my mind.

**M**: So when you drink where do you drink?

**P**: I buy and go drink in my place or drink very few bear at the pub

**M**: So where do most men spend their free time?

**P**: There is a big kraal here so most men likes standing there have a good conversation about the fights they had when they were growing up and they would tease each other about not owning cows even thou they worked for longer time. Sometimes we watch sport together, we even buy drinks and have good conversations.

**M**: I will ask questions very directed at you now, I know you have been around in mines for many years. According to your experience how was working in mines for you, how was working in mines in your own views?

**P**: Mines are not a good working environment, unfortunately because of my level of education this is the only place with my bread and butter, if you worked a long time in mines once you retire you do not stay long. We just working because we are in need.

**M**: If you do not mind me asking, why did you quit school?

**P**: There was no money back then because we our parents used to buy books for us and it was costly, secondly schools were far we travelled longer distances to reach schools. We were looking after our parent’s livestock sometimes waking up very early take the cows to the fields end up going to school very late and we were being punished regardless of where you were and our father would punish us if we do not look after livestock. Things are better now everything is for free and there are even meals. In our time there was no such.

**M**: You mentioned that it’s difficult working in mines, what is difficult? Secondly if its difficulty why did you work in mines for so many years?

**P**: I endure because I know I do not have any other source of income, if I retire I will stay at home if you notice our kids work for them and their families so who can look after me, and their wives would never do our laundry so that means I will have to employ someone to do the chores for us. So I need my job even thou we do not know at what point are we going to get our pension funds.

**M**: So now you saying you looking forward to your retirement, what kind of men do you want to be at your retirement?

**P**: I would love to seat in my house, look after my sheep, live with my grandsons and leave with my kids and travel with their cars if possible.

**M**: Reflecting from the answer above, how far do you think you are from achieving all of the above?

**P**: I am not far but I won’t look into many things however I still need to make a kraal. I have checked prices of cows at least I can buy few cows and make my dreams a reality.

**M**: Who is your role model or that one single man that you look up to like Michael Jackson or Mandela who are those people, be it they are from your community or politicians. Who is that person?

**P**: I would love to be a brave man in the neighborhood who solve other people problem and advise them. Understanding that I won’t get into Mandela’s level because those people were very educated. I would love to be a man who advises other people in the community because I do not like bad things happening to other people.

**M**: As a man who have worked in mines for a long time how does that make you seen by the people both at home and here in Marikana?

**P**: People can see your achievements they see when you making difference in your home. Back at home people even see what you bring home during your visits. I have never sinned any one I do not have any cases so people know me I am a good man.

**M**: Does working in mines gives you dignity?

**P**: Yes it does give me dignity because back at home I am given platforms even in the community meetings (Imbizo’s) and a make very good suggestions and I sometimes question their decisions.

**M**: Does having a wife also give some dignity?

**P**: Yes, having a wife gives a dignity and having kids too.

**M**: I would like us to move forward and knowing that you have been here for years, almost everyone in South Africa knows that there was a big strike here in Marikana in 2012. Now I would love to hear your experience as a worker here during that period. How was life here during that time?

**P**: I was admitted in hospital for nine months, at that time of my hospitalization it was when the strike arose so I do not have any information of the strike. So I cannot be able to answer your question because I didn’t witness anything during that period as I was hospitalized.

**M**: I am glad that you got well, I am very interested to know how you managed to leave your life during that time understanding that all employees were not paid at all during that time.

**P**: I called my wife when things started to be terrible, I asked her to sell some livestock just to ensure that we are living our lives normally and that helped us a lot. So we agreed and eventually she did sell some of the animals and that’s how we managed to live normally.

**M**: Do you perhaps know how other man survived during that time, do they at least speak about how they survived during that period when they were not paid?

**P**: Some were sharing bread it was difficult, some decided to sell steel and iron junk to the scrap yard just to make money to buy food whilst some were sent money from their home to buy food. Some were sent money to go back home it was very difficult.

**M**: Thank you for your views. I hear you very well I think we have spoken enough thank you for being open. And thank you for asking questions even before we started this conversation.

**P**: Yes more especially this Dictaphone made me very suspicious and uncomfortable

**M**: We are using this Dictaphone so that I picks up all this conversation. We wouldn’t like to forget the important points you raised. I would not like at all to forget even a word of this meaningful conversation so it is the reason we use it. I hope my explanation prior to this conversation made it clear because I explained prior our conversation right?

**P**: Yes I understood.

**M**: Is there any other thing that you would like to say or any last remarks?

**P**: No I do not have anything, unless there is something that you have in mind or you can remind me.

M: I think I have touched on most of questions that I was supposed to ask.

**P**: Life is very difficult, I really do not understand this democracy if you notice all the boys who are behind bars are the ones who were born after democracy. They are there one who never grew up looking after cows and they are not disciplined at all.

**M**: Yes. I understand you, one last question since you worked for a very long times here in mines. I would like to know, what kind is a man that is considered a feeble?

**P**: There is no weak man but there are lazy men, those are the people that are not willing to give their hand. It happens that at times tiny man work much harder than chubby man because maybe the chubby ones are lazy to do the job. If you are lazy you are lazy but there is no puny man.

**M**: Thanks once again.

**P**: Thank you to you too.
