# Supplementary material for: “You see this thing is hard… ey, this thing is painful”: The burden of the provider role and construction of masculinities amongst Black male mineworkers in Marikana, South Africa
Source: PLoS One. 2022 May 23;17(5):e0268227. doi: 10.1371/journal.pone.0268227 (PMC9126392; doi:10.1371/journal.pone.0268227)
Supplement: S1 Data — (ZIP) [file pone.0268227.s002.zip › Anonymised Transcripts/INTERVIEW 711_0153_anonymised.docx]

**INTERVIEW 7110153**

**Codes**

***M = Moderator***

***P= Participant***

**M**: Let me thank you sir for giving me your time to go through this interview together. Please relax feel free and understand that this interview is solely for research purposes there is no other reason and all information will be confidential. We would like to understand lives of man living in here Marikina, please relax there is no right and wrong answer, we just interested in your views, experience and life experiences. Please feel free and speak openly.

**M**: Firstly can you please give me your age or your year of birth?

**P**: My I.D Number is [number]

**M**: So you saying you were born in [year]?

**P**: Yes

**M**: What about your marital status?

**P**: I am Married

**M**: Are you married traditionally, legally or both

**P**: I am married legally

**M**: What about your educational status, which highest grade you passed?

**P**: Matric, I got qualifications for Matric

**M**: So you passed matric?

**P**: Yes

**M**: How long have you been living here in Marikina?

**P**: From 1996 up until now

**M**: How long have you been working in the mining industry?

**P**: I started working in mines in the early 1980’s, but I am not sure because I started in [place]

**M**: Can you guess?

**P**: I would say between [year] – [year].

**M**: Which ethnic group do you belong to? Xhosa, Zulu, Pedi

**P**: I am Xhosa

**M**: I have prepared all of these question, we do not have to ask all them but is just a guide for me so that at the end of the interview I know that I have touched on some of them. Please tell me about yourself, your family, where you grew up and generally how you grew up?

**P**: I grew up in what we call villages, I was born in the Eastern Cape. I attended school there up until I Matriculated. We used to lie to ourselves telling us that we are well educated when we just have matric so after I passed my matric I dropped off school and joined mining.

**M**: Why are you saying you lied to yourself when saying you thought you are well educated back then when you have matric, why do you put it that way?

**P**: In the olden days people with matric when taken very serious as well educated people and they would have that sense that they are very educated too, also my parents never said I should quit school after matric but I told myself that I am matriculated and that I have achieved what I wanted, but I lied to myself?

**M**: What do you see now that you saying you lied to yourself?

**P**: Today there are other challenges that requires further education, that I am unable to achieve and if maybe I proceeded with my school beyond matric and listened to my parents I would have been something else today. In The olden day even people with lower qualification were seen to be very educated.

**M**: Now that you talking about your parents, can you tell me more about your family, how many siblings do you have and how you grew up?

**P**: I grew up in my family with [number] siblings so there were [number] of us “children” and I am the lastborn, because of poverty in the olden days some of my siblings were given to other families just like my brothers who were given to other families, but I grew up with my parents. I didn’t grew up very hash and I was not treated badly.

**M**: Did your other siblings go to school?

**P**: Yes they went to school, at least they are able to write their signatures, some of them have standard four and five I do not want to say they are not educated but rather literate

**M**: So what was your reason to quit school and go to mines please elaborate?

**P**: The only reason was peer pressure, when I heard that my friends were in Johannesburg I also wanted to go, there was no major reason like poverty or anything else because back at home we were not starving at least we had some small farming to do away with poverty unlike nowadays, so I just wanted to go so to be with friends no one forced me to quit school.

**P**: Can you tell me about people that you consider as your relatives who are residing in Marikina?

**M**: How?

**P**: Okay, let me rephrase my question, are there any people who are your relatives that also reside here in Marikina

**M**: Yes, there are. Although you will find that I am their guardian

**M**: Are they staying with you?

**P**: Yes they stay with me but one lives on his own in the other street but he is on leave now

**M**: Now that you saying you marriage, tell me about your marriage do you have any children

**P**: Life is something else sir. How? After I married my wife we decided to build our own house together then we left my home. After we left my wife got ill and eventually God took her (died) leaving behind one daughter for me. Our cultural belief confines us to stay just a little while before taking another wife, I stayed few years “two years” and I eventually decided to take another wife because I realized that I am delaying myself. I took my second wife which is the one that I am staying with. So we went to the magistrate to get married legally. So my wife stays in my house in Eastern Cape and we have two boys.

**M**: So as you are talking about your family I hear that you have another house in [place], please tell me more.

**P**: I do have another house back at home, I do not want to lie but I am here for work purpose. I am here because I thought about the future of my wife, kids and myself so I am not here forever, I came here for work purposes. The land that we live in here is not ours however we stay because we want to support our families, as we speak my children are at school and they are doing everything that other kids do. My kids and their Mom are very confident that their father is at work.

**M**: How important it is for a man to have a house, kids and a home, how important is that to you as a man?

**P**: I would say to me it is very important, staying without a house as a man or relaying on somebody else is unmanly, you cannot take a wife whilst staying under someone’s else’s roof otherwise there will be conflicts. You have to have your own place for your wife and your kids. Your kids and your wife shall stay free under your own roof.

M: I am very concerned about the noise, do you mind if we close the door and for confidentiality purposes?

**P**: No, I do not mind.

**M**: Thank you for giving me your views as an experienced man, now I would like to understand from you as a man working in Marikina, what are your views about a man who has never built his own house?

**P**: There are lot of challenges with that I do have experience, and I know there are challenges with that for instance if a man decides to take a wife whilst saying in his home there will be challenges with that because there will be lots of conflicts in that home more especially if there are other people staying under the same roof and that man cannot do as he please, We do have people who do not have homes because they never had long term goals and they end up here. There are times of death were people have to go home to be buried but you find out some people do not have houses. At times we end up negotiating with the municipality just for them to be buried decent.

**M**: Which roles do you think are important for a man or those you should perform in your family?

**P**: Your actions should tell that you are a grown man with a house, a man need to build his own house and in my family I should make sure that there are no complaints, understanding that there will always be complaints but at least I should ensure that my kids get primary needs education being one of them. None of them should regret later on in their lives because I did not work hard to fulfil their needs.

**M**: Now that you are a working man and living here in Marikina, how difficult for you to perform your roles and responsible as a man in your family?

**P**: There are difficulties, but one needs to plan and should thoroughly prepare before doing anything. I just made a good example about building, I bought all the material needed as I was building a room. On the [date] of [month] in [year] I got a call from my wife crying she couldn’t talk, I called my brother even him was busy he couldn’t hear me, all of a sudden my house was struck by thunder and was in flames. I couldn’t blame anyone but those are the difficulties we face, but we decided again with my house to rebuild another house we bought a plan and started with the construction and eventually our [number] room house was finished, no one goes against Gods plan. I am not saying these things are not doable but it’s difficult. There is also a cultural belief that a man should at least own some cattle and I bought myself two calves but they not looked after by me because I do not have a Shepard, however my brother who doesn’t stay far from my house back in Eastern Cape is the one looking after all our family livestock.

**M**: Why are you mentioning livestock Sir when speaking about your manhood?

**P**: it is important to mention livestock following the way I grew up. There are some rituals that should be carried out and they never happen without spilling of animal’s blood and these are the things that we believe in culturally and requires a man to have livestock so that we do not buy every time when we have a ritual. We do not have to leave our customs, when we grew up we used bulls to plough our gardens not tractors as we see these days that’s why we shouldn’t leave our customs I say those time have not passed entirely livestock is very useful.

**M**: On your own views and those of the community you grew in how does a community view a man who doesn’t have Livestock and or doesn’t and or doesn’t even have a house, how is that man seen or perceived by the community?

**P**: There many things that happen to that man, sometimes you find that he gets shy to attend some community events because he doesn’t have anything to talk about and even exclude themselves in community events or rituals so those are the challenges and he never gets free because he has no livestock.

**M**: Now that you say he gets shy, is it the community that makes him shy or is it something that come within him?

**P**: No, it’s not a community but even you yourself let’s say you do not have kids you can’t have a say when other people are speaking about children or getting school for children so it’s the same. Man would feel shy to do some other things in the community because there are things they haven’t done or achieved as man.

**M**: Let’s come back here in Marikana, a man who is working but doesn’t have any valuable things or do not show any characteristics of earning an income, how is that kind of man seen?

**P**: Most of the time it’s not important to involve yourself in other people business. Yes, there are such people but its best if their family members intervene. Some Man stays here for many years but hardly go home even when they are on leave instead they decided to go to their partner’s homes.

**M**: You said something very interesting you mention man who involved themselves in other relationships leaving their families behind, I’ve heard this even on interviews I had earlier about people who lives their homes get here and start other relationship and even starting living together with their new partners, how common is this occurrence?

**P**: It is very common in this community. Why, because even ladies leave their homes come here with intentions of making money. This start when these girls get pregnant at an early age, they fail to feed their kids then decide to come here and stay with other man but with no intentions of loving but rather for making money

**M**: This is getting very interesting can you explain further sir, you saying they got other intentions?

**P**: Love is different from working. How, remember we said we will take everything confidential

**M**: Yes

**P**: There is robbery “being interested” and real love “reality”. Some other times man would even approach married women and these women would agree to be on a relationship with these men because they know they will get some of the things they would ask for. Women would agree on the basis that they know they will earn something from this relationship, it’s not a permanent relationship but a temporal relationship

**M**: Remember I said all of this interview will be confidential and I said when there is any question that you feel you do not want to answer you are free to do so. Now I would like to ask you a personal question. Since you have been working here for quite sometimes have you ever been involved in these kind of relationship in your personal experience and how did you get into such relationships.

**P**: My involvement? There is something that robs people off “interest”, sometimes we get robbed meanwhile we have wives back at home I talk from experience and you’ll find out that there will be consequences back at home which even leads us to visit traditional healers and they would ask if do the man have any other relationships with other women and you would know right there and then that it’s the person you seeing whom you do not even love but you were just “interested” to (lust).

**M**: What would you say are the interest of the women in these relationship?

**P**: I’ve said this earlier on I will personalize these questions. When these women date men around here there are petty things that they want to get, they are sometimes after titles as you they even know our pay dates. Sometimes when dating someone you trust them and you even send them to a store but you’ll find that they do not return all the change or they ask very small thigs that they do not have and they know you cannot deny them, it’s even worse these days’ money transfers are close by they can even send money back to their homes at Shoprite. Sometimes they can even discuss you on WhatsApp. So the petty things they gain makes lot of different in their lives.

**M**: I hear you very well, what are your personal or general views that causes man to chase after these women.

**P**: You see I seat on very confidential meetings as you know. We are very different in our families. I have experience on discussing very confidential issues of people’s personal fights. Nowadays such cases do not go to the police station straight away but start with ward counselors, people fight alone and eventually come for advise and it happens a lot. In other families people will even have children outside of their marriages and things can get very tough with such cases when people find out that their partners are cheating on them.

**M**: So is this occurrence is common with women coming here and where do you meet such these women on the streets or on pubs?

**P**: Most of the time I will personalize these question, it has happened with my first wife’s daughter as I was working shifts; afternoon, night and sometimes morning shifts it happened that she was dating someone here that time she was studying here as a results the guy she was dating even went back home to pay Lobola (*Traditional Marriage Negotiations Money*) for her and my brother who was seating on the negotiations accepted this offer because my daughter agreed that she knows this guys. I couldn’t say anything when she has agreed to marry this guy I even enjoyed and get used to the fact that my child was married I even gave her some advice. I saw her coming here visiting her husband often. I do not smoke nor drink but she happened to be smoking and drinking and her husband is just like me he doesn’t drink nor smoke. People started saying things that my daughter was drinking and smoking and she denied all of this. Until one evening when her husband came to me and confessed that his wife my daughter was drinking and they had problems in their marriage because of her behavior. I tried to speak with her I took her home for my family to speak with her too but after that she distanced herself from us and concentrated on her friends as a result his husband too got tired of her. I saw her starting to wear trousers right here in front of me and people were talking and I was talking a lot with her up until when rumors went around that I am also after her people were saying that I want to have sex with her so I also decided to distanced myself from her. As we speak she stays somewhere here with another man, only she knows how she got into that her new relationship.

**M**: Thank you for sharing this incidence, Can you share your experience of working in Mines, How has it been?

**P**: I worked in mines, initially when I came to London Mine (Lonmin) I came to my brother and I was into politics when I first got here one day my brother told me that shaft one was employing I didn’t even know where that shaft was but I asked people to accompany me. I went there it was very packed I saw people carrying wooden sticks as if they attend a traditional ritual. So three men came to me and it happened that we worked together previously in another mine they told me that I should submit my Identity document I was given a form and on the same day I was asked to recruit supervisors and they told me that I should start with myself. I went to training and I worked underground for 2 months only and I was re-deployed on the surface.

**M**: You told me that you worked in mines and I heard you are on another position now, tell me what it meant for you being a man working in mines?

**P**: I never had different perceptions of mines or people working in mines whatsoever. When I got here I never suffered and I wanted my parents to feel that I am working so that they and my home not be different from other homes, we were paid a penny but we survived.

**M**: I am interested on the fact that you wanted your parents to be equal with other parents, tell me how successful are you with things you planned to do?

**P**: I did lot of things for my home, somethings my parents couldn’t even afford to do. I took my self to circumcision school and I got married with the penny that I got in mines as I said earlier on planning is important and mines have help me a lot. Money is not enough but I planned with the little money I had and that has helped me a lot

**M**: Thank you for this response, understanding that man have goals that they set to achieve before the time they take their retirement, so tell me what plans do you have before your retirement?

**P**: Even thou I do not have lot of things in mind but I do have things that I would like to do before my retirement. I would love to have my own house and be on a good state of health and be able to do thing for myself and on my own.

**M**: In terms of having role models, which man inspires you in life or that you look up to?

**P**: I do not want to lie I won’t say that I would like to be a particular individual. I do not like having competitions with other people, most people fall into terrible situations in life because they are competing with other people, chasing glamourous things and other things that other people have.

**M**: Can we shift a bit and go back to the big strike that took place in 2012 here in Marikana were most people died, can you tell me more about that strike as a person who witness that occurrence?

**P**: Although I was not an employee that time, but I was staying around here. The strike started because workers were demanding better wages. Management couldn’t meet employee demands and they couldn’t promise anything close to the demands. Employees decided to stop production however the miners couldn’t follow proper lawful ways of raising such demands and negotiating with the management, there are constitutional guidelines that has to be followed to raise grievances and these are decided by trade unions and chamber of mines. What happened here miners didn’t have faith in the trade unions and miners had no peaceful ways of negotiating with management. There were differences between miners, trade unions and mine management. Union [name] was not recognized then and miners had no representatives. The striking group was forcing every employee not attend work and they even raised threats to all other employees who were not amongst the striking group and some people died during this period. Some properties were vandalized because miners were occupying other premise without consent. The management decide to remove the employees on its property because of vandalism. The mine management brought in mine security guards who were later assassinated by miners striking group. Then the mine decided to bring in South Africa Police Services to intervene, most miners were therefore killed by SAPS because of their resistance and they didn’t want to move away from the mountain were most meetings were held. However, there were wrong interpretation of this situation even now people are insulting the president that he instructed police to kill miners even if some can hear this recording they will have negative emotion about me speaking this way but I do not have any fear.

**M**: Well this recording is very confidential as I said before, please do not worry about that. I would like to know more from the fact that you said most people died, from your own assessment do you think was there any vicious or violent misconduct by the miners?

**P**: Good question, that happened and it even affected the entire community most people were forced into meetings so the miners didn’t conduct themselves very well.

**P**: So would you say most men who live here who you know that came here to work for their families, during that period behaved differently or was there changed in their behavior. Did you observe any changes in behavior during the period of the strike?

**M**: Not exactly but I would say that is how people are here, sometimes I even overhear people over conversations talking about how they speak to their supervisors sometimes refusing to take instructions from them. People who are in authority or managing staff at work are being intimidated here and they fear to discipline their subordinates because they do not know what might happen to them out here.

**P**: How much was intimidation and force participation to those that were not willing to join the strike?

M: It was very common, the other day after the meeting in the mountain two guys were gunned down near the kitchen because they went to work. My neighbor, Fikile was killed because he didn’t want to join the strike but was consistently going to work.

**P**: So would you consider the man who were leading or being part of that strike as brave?

**M**: No I wouldn’t say so because I was not even being part of that and there other thing I was also a known member of [Union name] and not in leadership

**P**: What kind of man were perceived or considering cowards or were coward considered to be part of the strike?

**P**: No, because there were very rigorous preparation takin place, traditional healers were sometimes involved to perform charms to protect these guys against bullets I do not know how but very vicious conducts occurred and these never required cowards.

**M**: Thank you for your time and views Sir, your views are very important I came all the way to hear your views. Are the any last remarks you would like to say?

**P**: The strike robbed off people’s lives and took away other women’s husbands and these guys were bread winners. Miner were addressed about their conduct many times even Mr. [Union leader] addressed them and pleaded with them however some couldn’t listen. I am glad that today people have been educated about how they should conduct themselves and how they should raise their concerns with the management. New leadership has been appointed today people here now will not just embark on any strike because they have good leadership.

**M**: Would you tell me why miners had weapons with them during the strike, can you help me understand?

**P**: People had weapons with them because they were intimidating those that wanted to go to work.

**M**: How is the psychology of the community after the strike?

**P**: The community is still in shock more especially when knowing that innocent lives were lost because most people died going to work not doing any harm to any one, you can imagine.

**M**: Thank you for your time and your views as the leader for the community, we appreciate your participation. I have gained more that I hope for and more especially your hospitality.

**P**: Thanks to you too for asking openly. I also appreciate your effort.
