# Supplementary material for: “You see this thing is hard… ey, this thing is painful”: The burden of the provider role and construction of masculinities amongst Black male mineworkers in Marikana, South Africa
Source: PLoS One. 2022 May 23;17(5):e0268227. doi: 10.1371/journal.pone.0268227 (PMC9126392; doi:10.1371/journal.pone.0268227)
Supplement: S1 Data — (ZIP) [file pone.0268227.s002.zip › Anonymised Transcripts/INTERVIEW 711_0154_anonymised.docx]

**Tape number: 711_0154**

**Codes:**

**M= Moderator**

**P= Participant**

M: As I have said [name] I want us to be free and talk freely and know that there is no right or wrong answer, whatever we discuss here will remain between the two of us. All I am trying to say is be free and know that your opinion is important to me and say everything because what you say is important to me. I have questions that I have prepared as you can see that they are from here to here. I will not ask all these questions but it is just for guidance so that I know that we have talked about this and that. Do not mind this we are using it because*… (Phone rings and someone answers it).* As I was saying [name] can we be free and talk like man and don’t mind about this recorder. Can you first tell me your age and the year you were born?

P: I was born in [year].

M: Okay, [name]. Are you married or not?

P: I am married.

M: Are you traditionally married or married a white wedding?

P: Traditionally.

M: Which highest grade did you pass at school?

P: It is standard 10.

M: You have passed standard 10?

P: Yes.

M: Okay, [name]. How long have you been here in Marikana?

P: I started staying here in Marikana in 2009.

M: When did you start working here in Marikana?

P: I started in 2009.

M: Which race do you belong to? Would you say you are Sotho, Xhosa, and Zulu?

P: I’m a Xhosa.

M: Alright [name]. I would like to know more about you, will you tell me who are you, where did you grow up and tell me also about your family, how many were you and how did you grow up and all those things.

P: I was born in [place] in a location called [place], born and raised by both parents a mother and a father. We are [number] children and I am the [number] child amongst those children. There were no problems when we were growing up, we would go to school then come back and do the house chores. My father was also a mine worker and he retired from the mines in [year].

M: Okay, in terms of your siblings how many boy and how many girls?

P: There are [number] boys and [number] girls. I am a second boy and my eldest brother and the one following me are also working here in the mines. My eldest brother is in [name of mine] and the other one is here in [name of mine]. My sisters are at home and are not married.

M: How come your brothers and you are here? What happened?

P: We are here because of work and because it was not easy to go to school and we ended up here. My brother stopped at standard 9 at school and he came to work in the mines and my father was still working in the mines at that time. The reason why my brother left school was because we were all at school and my father could not afford so he looked for a job for him and we continued with school and I passed my standard 10 in [year] and at that time my father had retired already. It became difficult for me to continue with school after passing standard 10 and I had to come look for a job here in the mines in [year]. From year, year and year I was looking for a job all over [province name], I even went to [province name] but did not find any job then I came here in 2009 and got a job in this company, [name of mining company]. My younger brother was also not at school at that time so when I got a job here I called him to come and stay with me and he got a job in 2011 in the same company as me. The main reason we are here it is because we could not study further.

M: From which grade did your youngest brother stop at school?

P: He stopped at standard 9, he did not pass standard 9 and he stopped going to school. The last boy is the one who managed to go to school because last year he was completing his [Course name], he completed N6, he is now at home doing nothing. He was studying at College in [name of town].

M: Did he complete?

P: Yes, he completed the course he was doing because it was N’s and he is now at home doing nothing.

M: As you and your brothers are working, what do you think are your responsibilities at home?

P: My responsibility before I got married was to support my parents, my sisters and their children but now that I am married I left home and looked for a place to stay with my wife and 2 children.

M: As you were growing up, what are the things that your mother and father hammered in your head that you have to do as a man or to show that you are a strong man.

P: My father said as a man you need to learn to be independent, know how to do things on your own because if you dependent then you will not be able to survive in life when the person you depend on is no more. He used to tell us that we need to disassociate ourselves from doing wrong things like partying a lot because when you party too much most of the things go wrong. We grew up attending church and I am still going to church even now so I am far from doing wrong things and I am still keeping the values that I got from my parents.

M: What kind of a man would you like to be [name]?

P: I would like to be a man that is able to stand on his own, be able to take my children to school so that they can study further and be in a place that I could not be.

M: How far do you think you are from your dreams as you said you have your own dreams in order for you to be a man? What do you want to achieve as a man?

P: What I want to achieve is…for example as I am working here in the mines I don’t see myself spending the rest of my life here. I told myself that I will gather some money and when I see that I have enough money then I will search for other ways to make a living so that my children can grow well.

M: Why is that important to you [name]?

P: It is important because it is not nice to work under the conditions that we are working in, life is difficult for example I stay here alone, my wife is at home with the children and they cannot come here because there is no space for them to stay. What I would really love is to stay together with my children and wife in one place making decisions together because some of the things I hear them over the phone and sometimes not believe it so those are the thing I would like to see happening in front of me. I do not want to stay here for long for example I last went home in January and my wife can’t come here because the children are at school and they cannot come study this side because we cannot all stay in the shack in just one room. That is why I want to go stay at home and look for other means to earn a living.

M: What kind of things do you want to do?

P: Because I never had a chance to continue with school so I would like to have a business for example there is some money that I am saving so that when I decide to stop working here I can be able to buy a van or a taxi that will make some money while I am staying at home.

M: You said you got a job and worked then took a wife, why was it important for you to take a wife?

P: What was important to take a wife was to see myself as a man that has responsibilities, have a family and children. That is what I told myself that now that I am working I need to start my own family.

M: Where I’m going [name], I want to understand the reason behind…let me ask it this way, a man like you or same age as you that is not doing what you are doing how do you look at him or the community that you are coming from how will it look at him? Say this man is working but does not build a house, does not have a wife or does not plan for the future, how would you look at him?

P: As a person there are things that you wish your life to be like in order for you to be happy. Someone who is not doing what I am doing it means he has his own ways that he want his life to be like because we see life in many different ways and you will never do things the same way. Some tell themselves that my life would be better when I am doing things this way. That is why I took this decision about my life that it will be better when I am where I am.

M: As the three of you, your elder brother, your younger brother and you are working, what are the things that are your responsibility back at home that you need to do so that it can show that you are working?

P: What we told ourselves as we were breed by our parents is that our home should always be alright and things at home should be fine also if they are not feeling well then we should be able to take a responsibility of sending them money to go see the doctor together with our sisters that are not working. Make sure that they have food to eat and they should not suffer or run out of anything and the children that are still growing up can continue with school. When death comes in the family we be able to take care of the costs and when things like those happen we sit down and plan for the way forward.

M: I hear you [name]. How easy or difficult is it to do your responsibilities at home?

P: It is easy in this way…as I have mentioned before that there are 3 of us that are working…the easy part is to sit down and plan how are we going to tackle this problem for example if we need to give out some money, how are we going to be able to give out that money. But that does not mean you cannot do your personal problems, those that have no personal problems will take care of what is needed at home and you could be free to do your personal problems. Life is easy to us in that way.

M: Tell me more about you going to church as you mentioned earlier, why did you chose that direction?

P: I chose that for example as we were growing up going to church with my father he became a good example to me, he was a different father to other fathers, he talked good thing over us not bad things as his children. We were never beaten up, my father would talk to us nicely and tell us that this and this will not work. We grew up in church so I also liked going that route hence I decided to take it just because I saw the way my father is handling us. I also wanted my children to grow up the way I grew up and be a good example to them and also for them not to see me doing bad things that I wouldn’t want them to do.

M: You also mentioned earlier that the church takes you away from many things so there is a time as you are staying here in Marikana at Nkanini, there are times where you are off but not go home stay around here. How do you spend your spare time? Just give me a picture of how you spend you spare time.

P: most of the time as I have told you that I will go to work even today, I am always busy at work but when I am not at work I am here in the yard doing nothing. I sit here and clean my place, the only way I make myself happy is to play a radio that’s it.

M: How do other man that live here in Marikana spend their spare time?

P: Most of the time people go to a Sheeben as it is a weekend I can see that some are drunk already that is where most people spend their time. There are those that are playing soccer but they are few most of the man spend their time drinking alcohol and with woman. There are a lot of single woman here who are not working and mostly are found in the Sheeben so most man get happy that way.

M: As you said there are a lot of single woman that are not working, how did they end up here?

P: There are many of the ladies that are not working here. Some are saying they came here to look for jobs but they ended up not looking for any jobs but getting involved with man and staying with working man. What they usually do as soon as they find someone to love they move away from the places they were staying in to stay with that man and this is very common here.

M: I would like to emphasize on that because I think it’s important to me and thank you for telling me. You said they came here to look for jobs (P: yes) and end up falling in love (P: yes), when they don’t get jobs how do they earn their living?

P: They stay with the person they fell in love with for example it I am in love with [name] I will depend on him, with the little money he is giving me I will be able to buy clothes for myself. But most of them for example if they are in love with me, there are certain things that they do so that their need can be taken care of like having another man on the side so that when I give her a R200 she can get a R100 from the other man so that she can be able to feed her parents and children back home but staying with me. When she finds herself in that situation she will not think about anything else even what she came here to do, she will end up living that life. Then if I have discovered those nasty things that she is doing then I kick her out of my house because I thought that as I am staying with her doing everything for her she will be with me only and she will end up staying with another man elsewhere.

M: What you are talking about [name] is it something that everyone knows here in Nkanini?

P: Yes people know about it.

M: How do these relationship start or where do they get these ladies? What kind of places do they get them from?

P: Some are found in Sheebens and around they are everywhere, you also see them on the streets and a place that is near here at Marika, that is where they mostly meet and they talk about love and they end up staying together.

M: What makes man to be involved in these relationships whereas maybe they have someone they are in love or married with back home and be able to stay with someone here?

P: What makes those things to happen is…how I can put it for example we grew up in different places, our fathers telling us…most of our fathers were mine workers and there were hostels at that time and they do not exist now, they have been changed to family units people are able to stay with their wives and children but only a few people managed to get those places. We believe that as a man when you are married you need to have a home so we believe that you will not have those things when your wife is staying with you so that is why man prefer that their wives remain at home and he will be here. As a man you have sexual needs and man end up falling in love with these ladies because he cannot control himself and six month ends without you being able to go home so they end up having those relationships.

M: I will be more personal if you don’t mind and ask you have you ever came across those experiences and how did you deal with them since your wife is far away from you?

P: For now I can say I am able to deal with them because since 2009 I have never been in such a mess and I am not saying I will never be but for now I can control myself. I spend most of my time going to church and at work and doing some over work because it keeps me away from all fights that are happening around here and I will hear about them when I get back from work that this and that happened without being involved. I like doing over times so that I keep myself away from all those things and from woman. That is the way I am trying to distance myself and that is why I told myself that this life will not take me anywhere. Let me try to be with my family and no see myself being involved in the things that I do not like.

M: Why are you avoiding this life? Please tell me more.

P: The reason why am I avoiding this life is because as I am staying here, woman are always coming here to lodge a case that “the person I am staying with does not give me money, does not support me, I have a family and so on”. With the money we are earning here it is impossible to support your wife at home and also support the one you are staying with here and you will never get anywhere in life so that is why I am distancing myself because my dream is to be able to support my children and for them to get everything they want. Some woman come here because their husbands are busy with other woman here and are not able to support them back at home so that is why I am distancing myself in such things.

M: Thank you [name] for sharing with me. As you are a married man there are things that come in between your marriage where you find there is a fight between you and your wife, how do you solve those problems?

P: There are problems like those where we don’t get along with each other at that particular time, what I usually do I leave her for 2 to 3 hour and then again sit down with her and try to solve the problem that made us to fight but it’s not a usual thing to fight with my wife we are always happy.

M: When it happens that there is some commotion between you and people of this community or at work with the workers, how do you solve that problem? If it happened you can tell me a story.

P: It does happen at work where you find that there is a commotion between me and a co-worker in cases like those we call a supervisor and tell him that we don’t see eye to eye about 1, 2 and 3 and he will try to solve that problem and we end up forgiving each other. I have never experienced it in the community even here in the yard there has never been a commotion between us.

M: I will talk about the mines. You started working here in the mines in 2009?

P: Yes.

M: How has been your experience of working in the mines? How have you experienced working here in the mines?

P: Working in the mines most of the time it is nice and at the same time not nice in this way, most of the time here we use explosives and they are affecting us health wise so it is not nice in that way. Even in the hospitals that we have here you would see that people are not alright although they will say its other diseases and you will find that it is because of these explosives most of the time because some people have weak immune system. TB is highest here in the mines because of the things we are working with in the mines, it is not nice in that way because you end up seeing that your life will be shortened because of your working conditions. It is nice on the other because you get money to continue with the living.

M: About the hard working conditions you are talking about that it is hard working here and there are risks involved in your life, what makes you to stay and work here in the mines?

P: What makes me work here at the mines is because I want to have money that’s the first thing. Even if I can say I am going to try something else still it wants me to have money. Another thing, when we were growing up the mines were not taken as a serious place to work in but as a place where illiterate people work in. That made us also to see that it is not a place where one would say “I want to spend the rest of my life here, it will just be for a short period of time”. Even if God has given you a long life because of the troubles that one faces at work it will be shortened and it is not nice that way.

M: Bhut’ [name] as you are working here in the mines, how does it portray you to other people that you are working here? (P: how does it?). Does it make you to be looked in a certain way [name] or makes you look like a certain mine working man?

P: Most people look at you as a working man who has a better life especially those that don’t know the conditions you are working under in your work place for example before I started working here I would perceive mines as a good and important place to work in because of the way people who work in the mines dress but when I started working here I saw that it’s a different place. Yes, back at home I would think that someone who is working in the mines has got a lot of money, off which there is money, but the conditions we are working under are not favorable.

M: Let me ask this in a straight way. Is working in the mines giving you any dignity?

P: Yes, it does give me dignity as a man because you can do things the way you want especially when you are a man it is important for you to do what you desire in life. It gives you dignity in a way that you are not depending on anyone but yourself. Some people may even come to ask for help from you as a working man because they can see that you can help them in a 1, 2, 3 way. That gives you power when you see that people come to you for help.

M: So other man see you as a different kind of man?

P: Yes, as an important man which is better than them.

M: How does that make you feel, [name]?

P: It makes you feel important when people come to you to seek for help and also to know how to do certain problems back at home and you see that you are a man when you can do 1, 2 and 3.

M: I hear you [name], [name] in terms of your projection, in terms of…role models which man do you think you would like to be your role model? What kind of a man or which man?

P: Oh, I will not mention anyone’s name but I would say I would like to be like people who are working in a better job than the one I am in now, people who can do things that I wish for, for example if I see a man that has a big beautiful house, driving a nice car of his choice, taking his children to the schools that he wants to take them to, those are the people I would like to be like. Sometimes when you don’t have money you tend to see yourself as a failure especially when there is something at home that they want from you but you cannot give them because of the little money you are earning and you would then see yourself as failure. So I desire man that are able to build their own home and also take their children to school.

M: Let me ask you the same thing [name], How much is important to you or does it give you dignity or not, to be a married man? If it does how.

P: It gives me dignity because you spend a lot on marriage and so if you have managed to get married, have your own house and stay with your children you will have dignity. That is also what our parents wish for us to do as their children, that we can find a job, get married and have him own family. For example now that I have managed to do that, my father is very proud of me, that his child has a job, his own house and raising his children. It gives me dignity in that way and it makes me feel like a real man.

M: As you are staying here alone and also mentioned that your family is back at home, who are your support system here at Marikana, people that you know that you can run to when things get tough? Maybe people I can say you consider them as your family here is Marikana.

P: People that I can run to when it is tough at that particular time for example as I am staying here, if there is something that is bothering me or if I need something I go to chancellor [name] (M: okay). He is the person I run to even when there is something bothering me at that particular time, I talk to him and he ends up advising me that “I must do 1, 2 and 3 this way” and I become free in that way.

M: How is your relationship with your brother that is here?

P: Our relationship is very good but as I am staying here in [name of place] most of the time, he is staying in a place called Marikana West. Yes we do see each other but I search for someone who is closer to me at that particular time when I have a problem.

M: I hear you well [name], I hear you. Ohm…as I have said earlier we will talk about the strike that happened here, we were watching it where we were for example I was in [place] but you were here (P: yes). Can you share with me your experience, what did you see and how did you experience it.

P: Mhmm, yes there was a strike in 2012 and I was there. *(P: Talks to someone else and the moderator asks that the door be closed).* I was there when there was a strike in 2012 and what happened was very painful especially to us who were there when other people died. What I have experienced is that when you want something you need to follow correct channels that is what I saw. Yes, what happened, happened that was wrong but I also think that we were not supposed to behave the way we behaved for example before the police came what happened was not correct because some people died before other people died on that day and they were killed by us workers. We did something that was wrong because everyone has a right to do what he wants for example people can choose to join any union that they like they should not be forced to do what they don’t want. People who were not working were also affected because as we were staying in the mountain some people would come to the houses to collect those that are left behind to go and join us in the mountain. Those people had rights to remain behind if they do not want to be involved in the strike. That is what I experienced and that it is important to follow correct channels if you have a problem.

M: According to you what did that strike mean to you?

P: what the strike meant to e is that people who are leaders of the companies do not care for us workers, about the conditions we work under and how much we are being paid because what lead to the strike was that the money that we are earning is little than the work we are doing. Working in the mine means that you have taken a big risk and then after taking such a risk you get to be paid little money so I saw that we are not taken care of as mine workers even those people we are working for do not care for us.

M: [name] as you said you were there, how did you experience that strike?

P: The strike affected me badly because at the end of the day people died and there are children who have no parents today because of that strike. At the end of it all the strike came with a change in our lives.

M: How did you get away [name]?

P: I would say that it was just luck for me to get away from that strike and maybe being a coward because I was there when the association leader was talking and when he finished talking he said “workers please move away from this place, and the police were many at that time, because they have already taken a decision to kill you”. When he finished saying that I got scared and the police had a gathering after that they went to their hippos, when they entered the hippos I started going and the other workers were shouting asking where are we going because it was not only me who left. Just when we were in a short distance the police started shooting and that is how some of us got away.

M: How did you feel as your leader was telling you that you should move away because the decision have been taken?

P: I did not feel good because as the leader was saying that, they took it as if they are just scaring us because he mentioned that as the police are here they have been mandated to come and kill you. Some people said they should come because they took it as if they are scaring us, they will not do anything to us. As I said that some of us when the president of the union told us to move we moved and some thought they are just playing nothing is going to happen.

M: As you were in the mountain were you scared or brave? What was happening?

P: Bravery was there but some were afraid.

M: How important was it for man who were there to be brave or how do you see it? What kind of men was needed in that strike?

P: Brave men were needed but I wouldn’t say being brave is to be able to carry a weapon for example and tell yourself that you will do things by force. According to me a brave man is someone who is able to stand on his word and say 1, 2 and 3 should be done and we are not backing down and that would not mean that you will be violent towards the people you are dealing with for you to be seen as brave. To me a brave man is someone who do things in a correct manner and stand on what you want even if they are telling you that it is not going to work out.

M: As we were watching the television we saw workers carrying weapons, please tell us why man were carrying the weapons.

P: What made man to carry weapons ohm…let me start from the beginning, on the first day the workers who were RDOs gathered together…

M: Who are RDOs?

P: Machine boys…they went to complain that they are working hard and they want a salary raise so they took that decision for us and they told themselves that we have different unions here but when they are going to where they are going they are not going to include any union, they are going to get together as RDOs and go to the company’s offices to raise their concern. As they did that it was find out that they have taken a wrong step because one of the leaders of the union that exist here said “you cannot come here on your own without whereas we are here as your leaders”. The workers agreed and went back and they said as from today no one will go to work until our concern is taken care of. Then later in the day we heard one of the union leaders through the speaker saying we have to go to work and not listen to those people, if there is anything we want we must go to them. After we heard that, it was decided that we are going to gather here at Vonderkop and talk about the way forward. It was then said that the union leaders are saying we cannot go there ourselves to demand money, what is better is for us to go to the union office to tell them why we have decided to go there on our own. I was also part of that group when we went there although some workers were not all there because we met most of the workers when we were coming back. When we were about to reach the offices the group that was in front…we were not carrying any weapons on that day…the group that was in front was shot at and we turned and ran away and they also came running. We ran a distance from the rank here a Vonderkop upwards and when we reached the top we discovered that we don’t know where the gun shot came from because in the union offices that we were going to there is a police station next to it, so I thought that maybe it’s the police that are shooting because they don’t want us to go there. When we were here in Nkanini on the grounds we saw the men that were shooting at us and they were not the police but leaders of the union that we were going to. The workers took a decision to take their weapons and fight back because it was clear that those men were fighting with them. As they were chasing us from the mines grounds until here in Nkanini, one of the workers the following day suggested that we must go find a place where we are going to stay in and decide what on our next move. That is where now we decided to fight back because they are the ones who said we must not go to the employer ourselves we must go to them and now they are the ones that are shooting at us when we are going to them. That was the course of the weapons that were carried there.

M: At that time how regular was the use of weapons?

P: It was regular because as I have mentioned that the way things happened it was not supposed to happen that way. There were people who were working and when it is heard that a particular person is working, it was decided that we must search for him and be killed. The weapons were regularly used at that time.

M: Were these weapons used, when the decision was taken, by people who are violent by nature or were they changed by this strike?

P: The strike changed them, what made people angry is what the ruling union at that time, as we saw these man as people who can talk on our behalf we elected them and they saw us as people that are not important to them because when we went to them they could not sit down with us instead they shot us, so we decided to fight back. The strike changed the workers and it was the circumstance that made them to be brave. Because we elected them so if they don’t want to listen to us let us also be violent towards them as they are violent towards us. I am saying so because the following day we decided to take our weapons along with us when we are going to the office because they were chasing us with guns the previous day.

M: How ready man were in using the weapons at that time?

P: It was high because even those man that did not have the weapons thought that they were not strong and not man enough, so they decided to get the weapons and join other man.

M: How was the living for a man who became a coward and not join the strike?

P: Man who were cowards were not involved in the strike but most man felt that if they do not become part of the strike it would be like they are against it so they decided to join. And besides you will be seen as a man that is weak if you are seen around the houses not doing anything or they will say you are a coward so no man wants to be seen as a coward (Laughter) because there is a saying that says “you must be brave as a man and be able to face any obstacle”.

M: People died on the 16^th^ of August if I’m not mistaken (P: yes you are correct) you said you were there and you ran away, psychologically what happened to you after seeing that?

P: It was very painful when that thing happened in such a way that after what happened late on the 16^th^, early morning on the 17^th^ most people took their bags and went home because no one knew what next was going to happen *(Alarm disturbing in the background).* It was very painful and sad. When I got to my place I was thinking, because I got away from what was happening, I want to go home but because I did not have money I didn’t go then in few days at home they deposited money for me to go home and I left because of the painful thing we saw. It was not nice to see people dying in front of us, so that left us not knowing what will happen next and how safe are we since we escaped.

M: How was the stay here at Marikana after that?

P: After that violence became more here at Marikana, people were killed and it was not easy for the police to come search for those violent people because we hated the police for killing our brothers so they should not come close to where we are staying. Wrong people took advantage of that because they knew that the police were not wanted so they did what they wanted and that is how violence became more.

*(Talking to a phone)*

M: *(Apologizing)* [name] we are about to finish in fact we are finished. Thank you for your time and I thank you that you have honored our appointment, you were not forced but you gave me some time to come and listen to what I had to say, thank you. I hope that you will do the same to other people. Thank you also about the way you were free and voiced out your views and telling us your experiences. It is important to us to know how man live, what are their challenges and what can be done to make their lives better. We hope that with the information we gathered from different people we would be able to make sense out of it and write some reports but when we writing them we will not mention anyone’s name or things that will identify that is was Yandisa and [name] that were talking here. We are done now, is there anything that you want to say or add on what we have talked about?

P: What I would like to emphasize is something about the strike for example we had meeting recently and we have a mentality that says we must use force in order to get what we want. As we are going to sit and talk about money even this year, I have heard that we will go on strike and I am afraid that this one will not be like the first strike and with many workers it seems as if violence is the correct way to get what we want but for now I would say violence has decreased.

M: Would you say Nkanini is a place violent place or it was just because of the strike?

P: I see it as place where police must always be near it because there is violence and places that sell alcohol are the contributing factors to this violence because they close down very late and some people change their behavior when they are drunk.

M: Thank you very much [name]
