# Supplementary material for: “You see this thing is hard… ey, this thing is painful”: The burden of the provider role and construction of masculinities amongst Black male mineworkers in Marikana, South Africa
Source: PLoS One. 2022 May 23;17(5):e0268227. doi: 10.1371/journal.pone.0268227 (PMC9126392; doi:10.1371/journal.pone.0268227)
Supplement: S1 Data — (ZIP) [file pone.0268227.s002.zip › Anonymised Transcripts/INTERVIEW 711_anonymised.docx]

**INTERVIEW: 711_0146**

***CODES:***

***M: MODERATOR, P: PARTICIPANT***

M: Alright Sir, we are starting now. Like I’ve explained, this is a recorder to record our conversation. So please don’t mind it, just talk freely. I will put it over here. I know that Zulu is not an easy language for you but don’t worry you can mix with Xhosa and Sotho where you get stuck with Zulu. You see I’m writing 001 here meaning you are the first person I’m speaking to. Let me start by asking your age please.

P: I was born in [year].

M: Oh you were born in [year]. Ok Sir. Are you married Sir?

P: Yes but just a traditional Sotho way.

M: Oh you married traditionally?

P: Yes traditionally.

M: Ok. What is your last grade at school?

P: Standard 7.

M: Oh Standard 7?

P: Yes.

M: Ok. How long have you been staying here in Marikana?

P: From 2003

M: Ok. How long have you been working in the mines? When did you start working in the mines?

P: 2006.

M: Ok. You started working in the mines in 2006 but staying in Marikana you started in 2003?

P: Yes.

M: Ok. So are you a Pedi or Venda or Sotho?

P: I’m a Sotho man.

M: Ok. Thank you Sir, I have your details now. We will continue with the interview now. Like I earlier explained there is questions I would like to ask you. There are a lot but if we make them conversational, you won’t need to answer all of them one by one, they will be covered on the conversation.

P: Ok.

M: Now Sir, please tell me about yourself, your family, where you come from and who are you staying with here.

P: I grew up in Free state and my family is here but just a few, most of it is in Lesotho. I grew up in Free state and came here to look for work in 2003. But I stayed here looking for work from year, year, year and only found work in 2006.

M: Oh you started working in 2006?

P: Yes. I work for a contractor though, not the mine.

*(Too much background noise: a woman chatting to another one)*

M: Oh there are people working for the mine and people working for a contractor?

P: Yes. I work under ground for a company called Rawson. But the money we get as contractors is very little compared to the people employed directly by the mine.

M: Even though you all go underground?

P: Yes the miners get more money.

M: How do you feel about that?

P: It’s sad knowing that you doing the same job but the other people get more money than you.

M: I can imagine.

P: But work is difficult to find, so you just need to endure.

M: Yes Sir I hear you. Now tell me, you’ve been staying here for a long time since 2003, do you have people that you consider your support system since you are far from home? People that you go to when you have a problem?

P: I don’t have a family here in Marikana. We just two here, the other one is on leave and went home. But life is life it depends how you leave it.

M: This person you talking about is your family member, are you staying together here?

P: No he’s not staying here, he’s a man just like me. He rents somewhere else.

M: Now since you are staying here, do you have people you leave with here that you consider family even though they are not your biological family? Tell me about those people.

P: One of those people is the one I was telling you about, my brother and others are our home boys. I go to them when I have a problem because sometimes problems just come unexpectedly. They always help me.

M: So when you tell them you have a problem, they come and help you?

P: Yes Sir but not all of them. Some hide, some come, you know how people are.

M: *(laughing)* Yah I know. Let me thank you once again because I can see you are talking freely and I’m sure it is because before we started you asked me a lot of questions wanting to find out if this interview is legal.

P: Yes.

M: Now Sir, please tell me about your family back at home: your childhood, the people you grew up with, going to school…you said you were born in [year], so please tell me about your life from there until you came here in 2003.

P: Eish! Growing up was difficult because my father was not working. He used to work at [place] but lost the job when the strike broke out. There was a strike there in 1977, that’s when he lost his job. And he passed away last month.

M: Oh I’m sorry to hear that.

P: He was not working, it was very hard to continue with schooling that is why we ended only in standard 7 and did not continue with schooling. It was very difficult growing up. We were hustling for small jobs while growing up until today.

M: I hear you Sir. Do you have siblings and where are they now?

P: My elder sister passed away. Now it’s only me and my brother, the one I come after. He is the one I was telling you that we are together here.

M: Oh he is the one you are staying with here?

P: Yes. Otherwise others are just relatives. They are working here and the other one is in Cape Town.

M: Working there?

P: Yes working there.

M: I hear you Sir. Now can I please ask you…as a man, what do you think is your role in your family?

P: My role?

M: Yes, I mean like your responsibility, what is your responsibility?

P: It’s working for my family and taking care of their needs. Making sure life is easy at home because you are working. You see, it’s like that.

M: When you saying life is easy, what do you mean?

P: You see Sir, when you working there must be a difference between you and the person not working. You are able to buy groceries and send money home, but if you not working, you can’t do that. But some people don’t send money home and now their kids suffer, they walk bare feet to school and at home there is no food, but the man is working. That is not right.

M: A man that is failing, like you said, he is working but the community sees him as a failure, what would that person be like? I’m trying to say, in order to be a good and providing man in your family, how must you be like?

P: Firstly, as a man you must have a home, build yourself a home. After that you must be a man that goes home to see his family and then come back to work. You must work for your family. Yah now you will see that you are a man. And you must not walk around going to wrong places at night, a man doesn’t do that.

M: Wrong places, what do you mean by that?

P: There are people that would go to the tavern and come back at night. There is no man like that, no. You come back at night and the kids are watching you. Sometimes you even come back home drunk and abuse the children. You see, things like that are not right.

M: Oh I hear you clearly Sir. But if you look now Sir, you’ve been here since 2003, the Marikana man, how easy is it for them to do these things you were talking about, providing for their families and raising their kids?

P: We are not the same as people, our backgrounds are different and we were raised differently. Some people like nice times, they forget where they coming from and where they going.

M: Now you’ll see that I’m interested in the part where you talk about nice times. Please tell me more about that. What are these man doing in those nice times? What’s happening in their lives?

P: You see, some people when they get paid they don’t come to work. They ‘groove’, they call it ‘groove’.*(having nice time with friends)* A person takes money and go to [place] to have fun, because he got paid, but he stays here in [place]. And he will spend the whole weekend there spending money. If the money is not finished he won’t come back. If he comes back, he will spend it here having fun and not going to work until the money is finished. Sometimes he will spend the whole week here spending money. When he comes back he comes back with a sick leave.

M: Sick leave?

P: Yes sick leave. You see, a thing like that.

M: So Sir as you were saying that you are trying to do the best for your family, how difficult is that?

P: It’s very difficult Sir because the money I get here is very little. Even if I want to build a house or buy a car, I can’t do it because of the little money. I have two kids, they go to school and they use transport that I pay monthly. I buy clothes for them and food, but other things I don’t manage to do for them.

M: I hear you Sir. But now, tell me, as you are saying the company disappoints you by giving you little money, what makes you continue waking up to go to work?

P: It’s being poor and this little money I get here, no one is going to give to me and help me when not working. So this little I get, if it manages to help me buy clothes for the my children and for myself, then it’s better than nothing.

M: So now Sir, your wife and kids know that you are working but when they call and ask for something you don’t have money for at that time, how do you feel?

P: I tell them how much money I get and if they need something that costs a little bit much than I have, I ask them to ask in advance so that I can budget for it.

M: And if your child calls you and ask for something and you don’t have money for it, how do you feel about that as a father.

P: Eish! It’s very painful.

M: Now Sir, you have spoken about how you would like to leave a better life. In order for you to say ‘I’m now a successful man and leaving a better life.’ What kind of life you want that to be or how do you think you can archive that?

P: Just having a better life and manage to do all the things you wish to be able to do.

M: Ok I hear you Sir. Now I would like us to shift to another subject, but thank you very much for sharing your story with me. If there is anything you would like to add, you can add it later. You already told me about the men around here how they like to have fun and all that. Now please tell me more about that. When the Marikana men are not at work on weekends, what do they do?

P: It depends on each person how they want to spend their weekend. For example I do my washing and cook on weekends and then just rest. And others they do their own thing. Some go and drink and have fun.

M: This thing of alcohol, is it a norm here with the Marikana man?

P: It’s alcohol *(laughing)* there isn’t nothing else they have fun with.

M: *(laughing)* Ok.

P: You see. Just like me, if I have something I’m thinking about and it’s bothering me, I go and buy one or two and come drink in the house, just to forget this thing I’m thinking about.

M: Ok. You’ve spoken about how men here are…you see, I’m not from here, so please make me understand what activities do men do here when they are free? Those that can maybe say ‘ Yah I’m a real man on the right path.’ I know some maybe don’t like going out, instead they just buy one or two beers and drink at home. But just in general where do men go when they off work?

P: Ok. Others go home when they are off and those that didn’t go home they stay here, drink and have fun the whole weekend.

M: So please explain to me Sir, where they go to have fun, is there a tavern or sport watching?

P: Yes there is a tavern and a sport ground, so some go and play soccer. Like that.

M: Ok. I’ll go back to an issue you already spoken about, please don’t get bored that I keep going back there. Alcohol is another issue we think is important. How big of a problem is alcohol use between men and women here, if it’s a problem?

P: How big of a problem?

M: Yes.

P: It’s a problem because others drink at night and when they drink it night it happens that they end up stabbing each other. You things done at night are not right anywhere.

M: So from your point of view, what do you think causes the men of this area to drink alcohol too much?

P: It’s because of money, these people have money and some they use money as if they borrowed it…

M: As if they borrowed it?*(laughing)*

P: Yes as if they borrowed it. They abuse money, they use it to finish it now. They do things like that.

M: As if they borrowed it?*(laughing)*

P: Yes they abuse it even though they know how hard we work for it.

M: You see some men they go drink with women and spend money on them, how popular is that here?

P: It’s very popular, if you are a man and drinking you won’t stay in a tavern at night alone. Those that stay there until late night, they stay there because they have women they drinking with. You see if you drinking alone, you will finish early and go home because you know you are alone. But if you are with a woman and now you want to go home, the woman says ‘no just wait a little bit.’ Because she hasn’t had enough. And now you end up very drunk.

M: Ok, I hear you clearly Sir. Now I would like to ask you about relationships. You said to me you are married. You are married and maybe have a mistress here as well, I would hear that from you if you are comfortable sharing it. But what I want to know is, if you have problems in your marriage or other relationships you might have with other women, how do you resolve it?

P: When you are staying with a person in the house and eating from the same plate, what’s important is to seat down, talk and resolve the problem. The other person must not just be angry and keep quiet, that won’t solve the problem when you not talking about it.

M: Ok. Sometimes if this person you are trying to resolve the problem with, you wife or you mistress is slow, how do you resolve the problem when it comes to that?

P: When you are leaving with a person, you get used to that person and know how to handle things when taking them to the person. Because you know the person’s heart, you become patient with that person.

M: Ok. Now Sir I will base this question on you and the men you leave with here, your colleagues or your neighbors. If you have a problem with each other, how do you resolve it? Maybe you didn’t like the way the other man spoke to you, how do you solve that issue?

P: When you staying with a person you must talk to each other and resolve the issue. A person must not just get angry and keep quiet as that won’t solve the problem. The important thing is to seat down, talk and resolve the issue.

M: Sometimes when you see this person you are married to or your mistress you trying to resolve the problem with is slow, what do you do in that situation?

P: When you leaving with the person you get used to that person and know how to handle things that you take to that person.

M: Ok. Now as men, I mean the men you work with or your neighbors when you get into a conflict how do you resolve it?

P: We seat down and talk about it. If we can’t resolve it, we take it forward to our superiors and ask for advice. If it’s still not resolved then one will be fired and one will be given a warning. Something like that.

M: Oh, I hear you.

P: But we first talk to you as men and tell you that at work you must not misbehave.

M: And here in the community how do men resolve conflict, I mean here at [place]?

P: It depends on people if they want to resolve it, then they do. But if they don’t want then it goes to the police station in order for them to stop it.

M: Does it even get to the physical part, where they fight?

P: Yes.

M: Ok. Alright Sir, thank you. I would like us to move on now if you still ok to do so. Are you?

P: Yes. I’m alright.

M: Alright Sir. I would like to ask you some more questions and like I explained outside that I will also ask you about the strike. You as South Africans with beautiful hearts, black or white, we were really shocked when we had about the strike and that people were killed. So we want to know what can be done for people not to be killed that way? Because that was ugly. As we discussed earlier that the miners’ strike happened in 2012, as a man that was here at that time and saw where it happened, by that stone in the mountain. Please tell me Sir, you were here watching and saw how it happened. Could you please tell me how it happened? Talk about what you comfortable talking about.

P: You see that stone, I was seating on top of it watching. Many hippos came and it was soldiers. [Union leader] came to talk to them and then came to us and kneeled down and begged us “Please leave, these people have finalized about you, they will kill you.” You see. But you know people when they want a thing, they want that thing. They said “They can come kill us here.” And after [Union leader] left eish! It got very ugly. The hippo put a fence around us, so that we don’t come this side but go to the bushes side so that they can shoot us there on the bushes. You see, it was a thing like that. As for me, because I was seating up there on that stone, I could see that no these people are fighting for real now. So I got off and started walking away. I walked past those electric poles, you see them. I didn’t go to the bushes. You see, a thing like that. They now started shooting people and the helicopter opened a thing up there and started shooting “q, q,q,q” *(making a clicking sound)* People started falling down.

M: What was that Sir?

P: That thing the police use, the…*(thinking)* the tear gas.

M: Oh ok.

P: They poured a tear gas.

M: Sir please tell me about you and other people, but I guess you can talk mostly about you.

P: Eish! I was very scared Sir. When a person is holding a gun, that person is not playing.

M: So Sir when [Union leader] told people to go, you think people were brave or they were scared?

P: They were scared. And they were not happy, because here at the mine Sir we suffering a lot. We are working very hard and in a very dangerous place. And there is no money, you see. Like me now I can tell you that I take a lift and go down very deep in the mine hole, you see. And taking a lift is not nice because it gets stuck sometimes and not go down. And I’m working for R4 000.00

M: Yho! Mmh!

P: You see, it’s something like that Sir. You hear Sir what I’m talking about?

M: I hear you Sir. On this thing you were talking about, we will go back on the money issue When you were seating there on the stone and seeing all this happening and [Union leader] came to tell them to go, but they said ‘no let them kill us here, it’s better to die here.’ What did that mean? Because you said people were scared, yet they continued to say ‘it’s better to die here.’

P: They are tired. You know there are times as a man that you say ‘no let me endure’ but you get tired when you don’t know until when are you going to endured Sir. You see, it’s a thing like that.

M: The time you were seeing that these police are putting fence around you and you saw that things are really getting ugly, was there any talking amongst men?

P: No there was no talking. Each person saw that it’s really ugly now and they started running to different sides. And now the police were also just doing their job and also figuring out how to kill us.

M: Ok, I hear you Sir. How did you feel after that happened Sir?

P: I was afraid Sir. I came back to the house and that time my wife was with me here. I came inside here full of tear gas.

M: In the chest?

P: Yes in the chest. When I got here I asked for water and I was very scared. And the next day I went home.

M: Mmh.

P: Yes. I saw that, no here we will be killed for real.

M: I hear you Sir. You as a man Sir what do you think you did as a mine to survive that strike and not die there.

P: I ran Sir. It’s running that helped me there. And running knowing that you will be killed if you run that way, so rather run to the shack side. Because if you run to the bushes, eish! Those people were too many. There were ones that riding horses, others with dogs and others up there with the helicopter. Eish! And others were chasing us with hippos. And now if you run to the bushes they chase you easily, but if you run through the shacks, they can’t easily chase you. That’s what helped me. Others were shot and injured, a lot of them and some died right there.

M: The strike happened and all the dangerous happened. What did that strike mean to you?

P: Nothing, because that strike didn’t make any difference for us as contract workers. The miners are the ones that benefited and got what they were striking for, even though it wasn’t exactly what they demanded, but they were fine with what they got.

M: When we were watching on TV we saw that it was men that were striking, how important was it for men to go on the strike?

P: You see as a man when there’s a strike you must go and be part of it because if you don’t go other men will come back for you and say ‘we went to fight for our rights and you didn’t go fight with us, why?’ So that will make you not to be in a good standing with other workers. You see, that is why when people are going it’s important that you go with them. Because you know when people are mixed with others their minds are different, others are like this and others are like that.

M: So Sir there are places you would prefer not to go to but because you are man you feel obliged to go.

P: Yes, it’s just like the strike thing. We all knew how dangerous it is to go there but something forced us to go there. You see, it happens that sometimes the miners stop us from going to work even though the strike is not ours, just because the mine is theirs and this is their place, they would just force us to stop going to work. It’s a thing like that Sir.

M: I hear you Sir. How did it happened that there were no women involved in that strike?

P: Women from the mine are not many, that’s why they were not involved in the strike. That strike Sir was very dangerous, women were not going to cope because of the way people were being killed there, eish!

M: I hear you Sir. Ok. When you looking at it Sir, the men that went to the strike as I’m assuming that not all men went, how were they viewed by the community, as brave and the ones that didn’t go as cowards? Was there a talk like that?

P: Yes there was something like that and they were even saying ‘if you are a coward go back home and stay there, you will hear about this on the radio. We don’t want a coward here that will make us loose what we want.’

M: So how the ones viewed as cowards treated at the time of the strike? As a man would you have been comfortable to say to another man ‘no I’m scared I’m not going there?’

P: No, you would never say that because people will laugh at you. You can keep that in your heart.

M: And the ones that went on strike, wont they come back and treat you bad?

P: No they wont treat you bad. The only thing they fight for is when there are people going to work while others are on strike. If you are at home it’s up to you how you feeling about being at home while others are on strike. And you keep wondering if they come back and find you seating in the house what will they do to you?

M: Aah! Ok. The time the strike happened Sir, do you think after the strike men changed in behavior and became violent people always ready to fight and use weapons? What do you think? Do you think the strike changed the way they used to be?

P: Yes Sir, they changed because they were serious about what they were doing.

M: What I’m trying to say Sir is, did you see a change in people? For example [name], let’s say you know him in a different way but now you see he has changed, he’s fighting and using weapons. So that time of the strike did you notice any change on these men? If they were humble and good people but after the strike they changed and used weapons?

P: People changed that time, even the church-goers. Everyone changed. They were all carrying ugly things, you couldn’t even tell this is a church-goer.

M: What do you think changed them that time?

P: I think it’s because of working under bad conditions Sir. Here at the mines Sir they don’t care about us. We just work because we are poor otherwise mine work is not for human beings. But because we are poor and there is nowhere else we can go, we just work.

M: I hear you Sir.

P: Mine work is a very big risk Sir.

M: Sir I haven’t worked in the mine but I hear you keep mentioning how dangerous it is to work here. Could you please explain to me how is it dangerous?

P: You see sometimes these big stones they fall on top of people sometimes. And space we work on is too small, you can’t even work standing. We work kneeling down on the ground.

M: Yho!!

P: The whole 8 hours you work kneeling like that.

M: Yho!!

P: The are some that work standing because the places are not the same.

M: I hear you Sir.

P: It’s like that Sir.

M: So Sir let me ask you something, we will get back on the Marikana strike issue. Now please tell me, as a man working in the mine, what has working here done for you.

P: I managed to marry my woman and build a two roomed house. You see, that’s what I did since I worked here.

M: Now since you managed to do that as a man, how does your community view you? As you are a man that has a wife, kids and has built a home. What kind of a man does your community see you as?

P: People just look at you, they don’t look at what you do or don’t do. And the strike thing drew attention for us, because now people say [place] has money. They now look at what you are achieving or not achieving. You see. And people don’t know that there is mine workers and contract workers. They just know that you working in [place], so you have money. They don’t know there is different companies here and they are many. Maybe I have a neighbor back home that is a mine worker and I’m working for the contractor. That neighbor will archive more things than me, because I work very hard to save money to archive my things. As for my neighbor, his things will done easily because he gets more money to be able to do a budget, unlike me.

M: I hear you. So Sir, I’ll go back to the question I asked you earlier on. You said there’s a certain type of man you would like to be. How far do you now think you are from being that man you want to be?

P: Yho! I’m still far from it Sir. There are dreams I would like to be able to make true. As a person there are things you wish to archive, then you can say ‘Now I’m alright.’ But now I’m not managing to archive those things because of money. You see Sir, when you working, you must have a budget. But now our salary doesn’t allow us to have a budget. When you come back with this money you find food prices have gone up, so now you can’t buy groceries with R 1000.00 because that won’t last for the whole month. You must at least buy with R 1 800.00- R 2 000.00 to last for the whole month. On the other side there is school kids needing uniform and month end the school transport wants money. You see.

M: That Sir, when you are thinking and judging yourself, how does it make you feel when you can’t reach your dreams?

P: Eish! Sir, I can just say maybe it’s God who put me in this position, so I must not complain and cry too much. I must cry moving forward and not cry standing in one place. In Sotho we say *“ Ho tsoswa moketa o e tekang.”(*the trying one get’s help)

M: Oh. I like that.

P: You see when a cow is very sick and is lying, it must show signs that it want’s to get up. When you go help it, you must see that it’s struggling but it wants to get up. That’s what I mean.

M: I like that. *(laughing)* So even if things are hard, you must keep on trying.

P: Yes keep on trying and not just give up.

M: And the man that is like that cow that struggles and give in, how is he viewed?

P: In Xhosa they say *‘Yindoda emathileyo’(a weak man)*

M: *(laughing)*

P: A man like that Sir…that’s why I was saying it depends on how we were raised. You see.

M: Ok.

P: As a man you mustn’t give up easily. You must keep on trying.

M: I hear you Sir. We almost finished, I know we’ve been talking for long. Can you please tell me about social clubs here, as you told me that sometimes people get off days from work. So when they are off which social clubs, soccer clubs or *‘stokvels’(*saving clubs) do they join here in Marikana? You’ve been here since 2003, you might know something.

P: Yes there are some I go to when I’m off. I go to the stadium and watch soccer after that I come home. Some prefer to different things on their off days. Some go visit friends or families in [place] and some in [place]. We not the same so we don’t like same things. Some like live soccer and some like televised soccer.

M: What about saving clubs where you see them meeting sometimes as a group, is there something like that here?

P: There is Sir. Like in my case, I have people that I save money with. That makes things a little easy.

M: In these saving clubs, let me ask Sir, there are many people here and diversity, Xhosa’s, Sotho’s, Zulu’s and etc. When you are looking do Sotho’s save alone and Xhosa’s alone?

P: Yes Sir, that’s exactly how it happens here.

M: Please tell me why is it like that?

P: We seat down and discuss that we should start a saving club and we agree on that.

M: By why Sotho’s alone and Xhosa’s alone?

P: The thing is, we don’t trust each other. One trust his own people. And now this thing we doing it’s not like in the bank where you will get there and use a deposit slip. Here there is no deposit slip, so you might find we give a person R 10 000.00 as his share when it’s his turn. But now when it’s his turn to give other people their share, he doesn’t want. So where will we go report that person because this thing we doing is illegal. We can’t go to the police and say this person took our money. You see a thing like that.

M: I hear you Sir. It’s not legal?

P: Yes this thing we doing is not legal and that’s the first thing we must look at.

M: Ok. Just the last thing Sir and we will finish now. I want to ask Sir, in the community you find that there are men dating women but there are also men dating men. Does that happen here?

P: I’m not understanding Sir.

M: Alright Sir, what I’m trying to ask is, back then our fore fathers use to tell us stories about how men date other men in the mines because you find there is no women. Does that happen here?

P: Oh now I understand what you asking. No that thing doesn’t happen anymore here because we have our women here now. The mine has changed the old place to nice houses now. You can even stay with your wife and kids here. Even if there is someone doing that thing nowadays, that will be because he is used to it, otherwise we don’t have that that thing here anymore.

M: But you know this thing I’m talking about?

P: Yes I know it because when I arrived here in 2003 they were still doing it.

M: Was it a well known thing, everyone knew that it happens here?

P: Yes it was happening but a long time ago not now. It was happening a lot because women were not allowed here, it was only men staying here.

M: So this thing does it happen in the house or somewhere on the side?

P: No they can’t do it in the house because there’s a lot of people staying here. They can only hide and go do it there in the dark.

M: Not in the house.

P: No.

M: Alright I will ask you a last question. You spoke about men, I know men talk and I also have a wife I know men talk about things. So I want to ask, I know a man would have a wife at home but here he will have a mistress, how popular is that here, does it happen or it doesn’t happen?

P: It happens a lot because most people we say they leave together. They are not married they just leave together. But some stay with their wives here, you call your wife to come here, just like me, I call my wife to come here and she stays here for a month or two then she goes back home. Mostly she comes when the schools are closed because if she comes now there won’t be anyone preparing the kids for school. So I’m the one that goes home to see them and then when the schools are closed I ask them to come visit me here.

M: They come here?

P: Yes they visit me here and go back when the schools re open. You see.

M: Ok.

P: So now the others that are just staying together don’t call their wives to come visit here because they staying with their mistresses. They just go home when they get time.

M: They don’t call wives to come here?

P: No.

M: Where do they get these mistresses?

*(Interview ended just like that)*
