# Supplementary material for: “You see this thing is hard… ey, this thing is painful”: The burden of the provider role and construction of masculinities amongst Black male mineworkers in Marikana, South Africa
Source: PLoS One. 2022 May 23;17(5):e0268227. doi: 10.1371/journal.pone.0268227 (PMC9126392; doi:10.1371/journal.pone.0268227)
Supplement: S1 Data — (ZIP) [file pone.0268227.s002.zip › Anonymised Transcripts/MARIKANA INTERVIEW 4_anonymised.docx]

**INTERVIEW 7110152**

**Codes**

***M = Moderator***

***P= Participant***

**M:** We can start (**…)**, I want you to tell me your name, where do you live **(00:15)** and where do you come from Bro (**…)?**

**P:** I was born in Pukeng (M: okay), my mother got married here in **(…),** but I was still young, I grew up here in [place], I come home to visit the time I was studying (M: okay Bro**…**), when schools were closed, my grandfather would let me to go see my mother (M: okay), then I grew up like that until now, but now I’m no longer staying in [place], in [place], I stay here with my mother I am not married (M: okay), I don’t have a wife, she passed away when I was still unprepared to marry her, I have one child with her, now this child is a female and she also has a child (M: hmm), then my child also passed away then I am left alone with my grandchild, now I am staying alone, the grandchild was taken by her grandmother they are staying in Pretoria.

**M:** So, you are staying alone here?

**P:** I am staying with my mother.

**M:** Oh, with your mother?

**P:** Yes, the one who gave birth to me.

**M:** Oh, I hear you very well, how old are you Bro…?

**P:** laughs…I am pensioner type, I am [number] years (M: oh, that’s right)

**M:** when did you get out of school?

**P:** At school I dropped out in standard 7, the problem was, I had no money to go to school and I was not in a good health and also the elder people, you see the elder people, those who are too old, they do not understand very well, they do not want to hear from you but you hear from them, they disturbed me from other things like school, there were so many cows here, so every time when I’m from school I had to fetch them from the veld/forest and milk the cows in the morning, so I grew up like that, before I go to school I had to first milk the cow and we used it in tea and the left one we make it amasi to eat with pap, again afternoon when I came back from school I went to fetch the cow and milked it, like every day, when I grew up and in high school I saw that this job was difficult for me (M: hmm), It was hard for my studies, we ended up fighting with my grandfather, he told me ‘it’s better to leave, you don’t want to stay with me here, you don’t want to listen’, and I was confused what to do because at home they would fight me again because I left school and I left grandpa, and would ask me what do you intend to do, then I ran to [province], I didn’t even know [province] I was just going there, I took my small bag and find a transport that sent me to Krugersdorp, when I arrived there, I got piece jobs and I was struggling very much, I didn’t get the proper job (M: hmm), up until now, I came back to mines in [place], It was in [year] when I started working in [place], now, that time they were **trampling** on us **(05:58- 0618),** that time we were not familiar with mines (M: hmm) they taught us , you kneel down, we put other things here ***(pointing)*,** you kneel down pick **(……)** and throw it that side, I worked there until I found that in 3 months I can’t go back there, it was not nice and I resigned. That time there was Mangope, Mangope was the president, he didn’t want to see the Mtswane doing nothing (**07:09),** when you are not working, and you are Mtswane, they arrest you and beat you up and say go out of the mine and work in the construction, do you have a passport. If you got lazy at work they would **bind/arrest** you at that time and say why are you lazy at work and you’d stay up to 3 months and to that 3 months they sell you to **emaplazin**i, you will work there in **emaplazini** for 3months, when that period ends they take you back to the mine again to work there, when I was tired, I resigned and the following day when I wanted the job, they employed me again (M: oh), yes they did, but it was hard because I thought maybe if I can change the section and go to another, I would found a better job than this one but all of these mines were the same, when I changed and go to another one it was the same, but I never worked for myself and bought myself something I like, or maybe just the wardrobe, I just bought a bed once and that time I was dating, I was forced by that woman to buy a bed, from then I never get anything, even after she passed away I never had a luck to get the job, I suffered until this time, if you don’t have a bonus you can’t get into mines, I always had piece jobs until I got the pension.

**M:** So, at that time you were already pensioned during the strike of 2012?

**P:** No, I was not, I pensioned in 2014

**M:** You were still working during the time of strike?

**P:** No, I wasn’t.

**M:** Let me ask you Bro… as a man, and you said you never get a proper job for a long time, how does it make you feel that you have nothing you’ve worked for?

**P:** Ey, I don’t know how I can tell you this, because you will take as if I am being out of control to all those things I wanted. I wanted to have my own car, my house, a wife and the children, but it didn’t happen and I’m in a bad space now, anytime I can die even if I can be sick my heart will be full, and I struggle, even if I get the pension grant to buy food and buy small things, that is not the money, you can’t buy anything.

**M:** So, Bro…as a man, how do you look at yourself as you didn’t achieve the things you wanted?

**P:** if I had money I would have my own house and found a woman even if I don’t get children but just to stay happy and not struggle that much.

**M:** As you are not working do you think that makes you not to get a woman to stay with?

**P:** Woman are no longer in love with man, they sell, if you ask for love they do not do that, but you can get by lucky, but these days no, they are many and they want you to buy, you give her money and she gives you her thing and that’s the end, then you go in separate ways, they are not up to this thing of loving each other, staying together, you can get one but they are no more.

**M:** You Bro…here in Marikana how people look at you as you are a man who is not working?

**P:** They see that this one is not working, and they see that if you are not working there’s something you are up to, maybe you are stealing or if not, you are doing bad things, people will never take you serious, they will laugh with you but when they are not in front of you, they are talking about you, they will call you names, saying that this person is not working maybe there’s something he does to survive, maybe he is a thief.

**M:** Bro…I want us to talk, you were there, during the 2012 strike you were around.

**P:** I was around but I was in [place] that time, (M: hmm), I saw here in [place] what these people did, like here I just heard when they told me from home that It was like this, and I also told them where I was, it was like this, you see, (M: hmm), because this thing didn’t happened in **(16:05)** it also happened in Pukeng (M: okay), in(**16:13**) in Mpala.

**M:** tell me about it, what was happening during that strike?

**P:** Others were saying that we must all go there and strike but others refused to go there (M: hmm), they didn’t knew what was happening, others thought maybe they will die there, they didn’t go there, and then if you didn’t go there, if they found you going to work and ask you where are you going, you replied saying you are going to work, they will be like, oh we are striking and you going to work, they will search your bags and find your work things and kill you, I remember another time they killed another woman, they cut her into two pieces (M: two pieces?), yes (M: from the heard?), from here in the neck, they cut her with knifes **(17:50),** sometimes they found you from the shop maybe to you bought your house things like washing powder, sugar, tea and they **(18:13),** they’d take your cooking oil and tell you to drink the whole bottle and tell you to go and others were killed straight.

**M:** Why did they do that?

**P:** They were worried that others are getting more money than them, because they were striking for money but others were not, others thought if they go to strike they will be wasting time because the children want money and back home they are not working but those people were blocking them on the way, saying that we are not going to work, we are striking, and you are not striking that means you are given money and after that they’ll kill you. They killed a lot of people even here in (**19:57**) there’s another mountain called **(20:03),** they stay on top of the mountain and striking there, fighting with the police**,** they were shot by the police **(20:24)** were shooting each other (M: with the police?), yes, then [police commissioner] came, she saw that my people will die so its better to shoot back (M: hmm).

**M:** Those people you are talking about, those of them who were forced to drink cooking oil, I heard that they were called as Amagundwana.

**P:** Amagundwana?

**M:** Yes, other brothers that I talked to, they said they were called as Amagundwana.

**P:** *Coughing*, I don’t know why they were called as Amagundwana, that mean it was just a name they gave them (M: okay), because amagundwana are the people who are working underground (M: hmm), yes, those were the ones who were called as amagundawna.

**M:** you told me Bro…about those who were working during the strike and others were forced to eat what they bought from the shop, and others were killed, what did they do to others they found from work?

**P:** They killed them very bad, they said you are going to work and we are busy striking and you think you are better and they kill them.

**M:** What did they used to kill them and Where did they kill them?

**P:** Wherever they found you, others were shot, and others were stabbed by knifes and thy cut the neck, and others were stabbed many times until they died, there was something called a **(23:18),**

**M:** What’s that?

**P:** Something that is sharpen pointed, it’s a sharp pole weapon, (M: okay), they used it to stab and kill you (M: something like **incula**), yes, it’s like a spear, if they slaughter you they will come out with anything inside you.

**M:** So, those who were doing this, are also other workers?

**P:** That also amazes me, why they did so, because those people worked together but they were upset by the fact that others go to work while others were not, others were allowed/permitted to go to work, so those who were fighting I don’t know what was happening to them, for just staying and wait for those who came from work and kill them like that.

**M:** How did they survive, those who were going to work, are there any people who survived?

**P:** Yes, there are those who survived, the road was closed, so they used another route, they went to the forest and come another side without being seen, because if they see you, you are dead.

**M:** When you think about them do you think those people were already bad people or people who always do this.

**P:** When I look it carefully, it was not the workers only, maybe they were called to come and do this **(M: 26:10-26:12)** because I don’t think if we work together and you make a mistake, I can come to you and kill you, other people were called to do this, and were told that this is your job and its your time to work this is your time to do so, because we are striking, those people don’t come without carrying the guns, sharp weapons and all these things, they came with their weapons to kill people.

**M:** That day when most people were killed here in (**27:17),** what happened to those workers who were in the mountain, to end up being shot, were they fighting back or?

**P:** They were busy engaging with the management and they disagreed, that is when they started to shoot the police and they killed the police, from there, they killed each other, that is why most people died that day.

**M:** When I think because I wasn’t there, and I want you to tell me, the people who were in the mountain, did they saw that there’s danger, why did they stayed there whilst the saw that those police would hurt us.

**P:** You know when I go with you carrying a gun with me and I say to you, do not be scared, when we fight you must fight back, if you run they would kill you, and they came with their traditional healer, s/he told them that they will not be hurt, only water that will come out of the police guns , they go with that say, but when I heard they also killed that traditional healer, because what s/he said didn’t work out.

**M:** After the strike how was the community?

**P:** I don’t know what to say because now when a person leaves, you see this place there on top, if they don’t know you, you won’t come back with all your stuff, they will rob you and kill you, even now if you go to work, at night they will kill you and sometimes you coming back from work they want money and they see you dressed up for work, and also at home they also do this but its better because they are scared, they do it here because there is full of sharks here, and they are not allowed to stay here **31:25-32:20**

**M:** So, people, after that strike, do they trust each other after that violence?

**P:** I don’t see people trust each other, there’s no such thing, it ended long time ago, you can trust me if we stay together but not other people, I’ll never trust other people, we can trust if we know each other but if not, ill never trust you or borrowing you money whereas I don’t know you, it’s better to take your id and bank card and keep them to me so that ill give you when you come to pay, people are untrustable.

**M:** Let me thank you for your time and you waited for the whole day so that I can finish with others, thank you very much for that, you would have told me that you don’t have the whole day, but you stayed and wait for me. Thank you, I don’t know if you have something to talk about with regard on what we were talking about or you ok.

**P:** I don’t have anything to say I was waiting for you to ask what you asked and give you the answers.

M: Okay thank you very much Bro….
