# Supplementary material for: “You see this thing is hard… ey, this thing is painful”: The burden of the provider role and construction of masculinities amongst Black male mineworkers in Marikana, South Africa
Source: PLoS One. 2022 May 23;17(5):e0268227. doi: 10.1371/journal.pone.0268227 (PMC9126392; doi:10.1371/journal.pone.0268227)
Supplement: S1 Data — (ZIP) [file pone.0268227.s002.zip › Anonymised Transcripts/MARIKANA INTERVIEWS 3_anonymised.docx]

**INTERVIEW 7110161**

**Codes**

***M = Moderator***

***P= Participant***

**M:** There it is Bro [name], we can start now. Bro [name] like I said please be free.

**P:** Okay no I am free

**M:** Okay Thank you very much. Can you firstly provide me with your age, are you married or not and what is your highest grade you passed?

**P:** I was born in [year], I am married I have [number] children (M: okay), my wife was born in [year], my first was born in [year], second born in [year], third born in [year], fourth born in [year] and another one in [year] yes, they are [number].

**M:** Where are they Bro [name]?

**P:** They are in Eastern Cape in Mount Frere (M: okay), but another one at the University in Queenstown, we are struggling with her, the one who was born in [year] but we are doing our best with my wife, another one is married, and the boy is busy with piece jobs and others are still in lower grades in school (M: okay), yes.

**M:** So, you saying when did you drop out of school?

**P:** I was doing standard 6

**M:** Why did you drop out of school?

**P:** The thing is, I impregnated a woman then I ran to look for a job in Gauteng (M: hmm), yes that was the problem.

**M:** When did you started working in mines?

**P:** I started in [year] in [place] in [place], then in [year] I took a leave, when we came back there was a strike, they were killing the Sothos then we got fired, we were given our belongings and we came back home by the same bus that took us from our homes, the ***Kostina,*** but we didn’t get even a cent, they robbed us, it was a year then I came back in 1989, I came to Western (M: okay), in Western I got employed in 1990 till 2002 when I was fired from the strike in the mountain (M: 2012?) yes 2012 I meant, so even then I was from the leave (M: hmm), it was also during the strike when I came back, then I decided to go to the mountain as I was told that all workers are there, I was going with others from the **Nkomponi,** they said they don’t want anyone with jewelery and the silver coin when you going there, then I turned back to put them in my hostel even my wedding ring, then after I put them when I was locking, in my hostel I always have my (**skomb…3:22**), when I was reaching the hostel I heard that something was happening then I came across the police van (M: okay), another one hitted me by the gun at the back then my teeth broke and I fell down, they took me and put me in the **guruguru**, it’s something big like a bakery you see these bakeries that sells bread (M: hmm) ,yes they throw me there, I was on the top and when I got there, there were also others who were inside and it was very dark in that car, they took us while I was hurt and the blood was bleeding I couldn’t even see others because it was very dark inside, I had no clue where we were taken to (M: hmm), then we arrived in Bethany, when I got there I saw that we are in jail now and we were taken there as many of us, we stayed there about 4 to 5 days (M: hmm), then they got us out, all people from **place** came with buses to take us, they were striking , when we came back we were discharged from work, we were always reminded that we will go back to work, we were not getting paid and others decided to go find job in Johannesburg and some in Cape Town or maybe others went to their relatives for an example (M: hmm). We didn’t get it even now, we were promised for the 12 between the 12 and 13, and now they are saying it’s handed to CCMA in [place], you see (M: okay), when I check to [company name] offices to ask how are things and they must give us money if we don’t get back to work, they said they are renewing it, they will call us I don’t know if on the 12 or 13 I will ask again but its next week (M: okay), that is the thing. I don’t know what happened I turned back to leave the things that were not allowed there, then I got injured. I was just coming back from the leave (M: hmm), I haven’t claim for my teeth I was not paid for that I didn’t get nothing (M: even now?) until now, [name] said I must take my teeth out but I said no wait Nozi they have to see it first, she said I must go to the clinic and I said no I still have to win the case first (M: okay), and show them how did it happen, if I take them out they will say no we know you were like this even before, but now they are not right, it’s irritating me, now its winter they are swelling I’ll have to put the scarf, that is the problem I’m facing, I don’t have another thing

**M:** I would like us to get back to the strike thing, so that we talk about it furthermore, Bro [name] you told me that you have a family (P: yes), when you look, what is the role of the man in the family, what is it a man supposed to be doing in the family?

**P:** He is supposed to support at home, the money should come from the father, everything, when you are not working, everything stops nothing continues (M: okay), so the time I came to work I had cows so when I have nothing I sell the cow, you see (M: hmm), I also don’t have money and I don’t know how to steal and I never stole, we sell the cow so that children would be able to go to school (M: hmm), that is the problem we are facing, you see.

**M:** I was going to ask exactly that Bro [name], so please explain it furthermore, as you were working all these years now suddenly you are no longer working, how do you survive as a man?

**P:** Now what I am doing, I have a house, I have 3 bedrooms in this house (M: here?), yes here in **(place**) so now I am renting out to two people, one from another room, they pay me R300 from each, so it’s the money I eat monthly, then at home if it’s the month end and we need something, I make another plan, I take the cow and sell it, you see, hoping that things will work out here but nothing is better, that is the problem.

**M:** How does it makes you feel as a man Bro [name] that you can’t survive without money?

**P:** Ey, you see my heart is weeping, now I can even work in the construction, there’s no other way I can survive, you see, but when I think again if I sell this house and go back home, but what am I going to do at home without job, you see (M: hmm), I will end up doing wrong things at home, you see, (M: hmm), they will say you are a thief, you see this thing hurts me, now I’m waiting for my child to finish her studies, this is her third year, if she can finish at least things will not be the same, there’s no way that I can send maybe at least R1000 because I’m not getting the old age grant because I’m still young for it (M: hmm), even if it’s a cent but things won’t be the same (M: hmm),that is like that.

**M:** Bro [name], as you worked for many years and then you lost your job, how the community looks at you as you are no longer working, are they looking you differently compared to the time you were still at work, or even at home?

**P:** Now that I am no longer working, friends are distancing themselves, it’s not the same as the time I was still working, you see, now I am tired of bothering people or even if I don’t have a soap I know that I’ll get this money and I’ll buy a lot of stuff I need without bothering people, because I see that people distance themselves and are saying “so even now you still not working no you don’t want to” (M: jho) and I’m starting to get confused because there’s no place that is recruiting around, you see, (M: hmm) I go all around this place but people say no you are not serious, things aren’t the same, people changed (M: hmm), no its very wrong to live this life, it’s very bad.

**M:** So, Bro [name] it happens, and I would imagine sometimes, something comes from home that, we need this and that and you cannot help that time how does it feel as a man?

**P:** Ey, it is hurting me very badly, you see, when she says I need something here at school, maybe it’s the money for rent or something and we are still in the middle of the month, and I see that there’s no other way I can do, I just ask someone back from home to give me R2000 or maybe depending on the amount she needs then I replace that amount with something, so I always make such plans of always calling people to give me the money, and I replace it with maybe a cow or sheep, that’s how I live (M: yes), if I had no cows it would be more worse.

**M:** Hmm, I get you Bro [name], I can imagine that all of us as we are growing up and as men, there are things we would like to achieve in life (M: yes), that, I like to be this kind of man, what are your goals in life as a man, what do you want to achieve?

**P:** Eh, you see what I wish for, eh my home is not right, if I can work I will have to build a big house then after that I buy another livestock because it helps me (M: hmm), nothing else, even the car is not a problem.

**M:** When you look Bro [name] at your dreams, where you are now and the situation you are in today, do you think you can still cover them?

**P:** Eh, now the age, but there are other things I can cover now but others I can’t because of age, because once you reach 60 years the company will retire you, (M: hmm), then you’ll get a pension fund, so from the years that are left, I am [number] years I can still cover in this [number] years left and try to squeeze things in so that I can get what I want, because there’s no other thing I’m looking for.

**M:** I hear you Bro [name] (P: hmm), I hear you very well, eh Bro [name] can we go back a little bit to the strike of 2012, you are saying Bro [name] you were coming from home……

**P:** Yes I was coming from home I was in the work leave, I’m not sure if it was Monday I don’t remember, when I left here there was a strike already but I was given a leave, then I went home and came back, but when I was coming back, on Sunday I called to check if they are still striking, then Monday I went to report in hospital that I am back, providing my papers and sick note, then because of strike they said I must come back another week and they said I can’t report because there’s still a strike, but this is the date and they put a stamp so that when the strike is over I can come back and report, then I came to my hostel to leave my things, I went to the colleagues in the mountain they when I was near I remembered that eish I still have my ring and it was said that they don’t want a silver coin, then I came back to my house to leave everything, and they caught me without my clock card even the bus fee from the jail, but fortunately we were taken by those buses who came to fetch us, I knew nothing other than that because I was coming from home, people were injured and they were only staying in the mountain, others were still at home on their work leave, but my leave days were over then I decided to come back, another problem when the strike was over I went back to the hospital, my record paper was not found so that means they took it, when I was on strike they stole it, then the company said I overspent my leave and I was like how, because it was this date when I came to report and I showed them, they said you overspent your days and they dismissed us, then I came back to the office it was [union name] but now is the [union name], I went back to the offices to give them my statement and showed them my papers stating that it was a strike and others were killed and you were there, they said I must come back to the hospital, the hospital sent us to the manager and the manager sent us to CCMA and so on, then it ended like that, to all of us who were arrested. I’m sure we were 68 (M: hmm), but the 49 of us were on this case and the rest got employed by another **(16:58)** they went to work there, and we waited like that until others looked for another job, it is like that. I don’t know nothing about what happened in the mountain because I was from the leave, there’s nothing I know there, yes people got injured as you saw but I know nothing I was still going there (M: hmm).

**M:** Tell me about, as you were on the work leave, tell me about the strike let us talk about the violence, tell me what made them to use violence.

**P:** What happened there, when I heard this, the workers as they were demanding the certain amount, they were paid a very small amount of money, so they were striking because they were demanding 12,5 that was the money we didn’t get we were only given R3500 to R4000 you see, so now the workers searched it and they found that we were robbed, they heard that from [union leader]**,** about what was happening and that you are robbed, the money you are supposed to get is 12,5, that is why when the whites told them to go to work, and they refused they wanted this money up until they signed it, so people were injured because they said they are not going to work, so when they were removing them from the mountain they used the hot water and they were shot and killed, the police were wrong because they were shooting them and killing them in the sharks (M: they were fetching them from the sharks?) yes, others were here, it was very bad, even now I don’t think such thing will happen again because we’ve lost so many people, our friends no it was very bad.

**M:** So, you saying people Bro [name], they removed them from the mountain by pouring the hot water?

**P:** Yes, they used hot water, it was hard to find them because there was no distance, and the mountain is very far, so they were trying to make the plan to reach them, they were running around the mountain. When I heard, they came for the first time, then those who were in the mountain came down to the police and the police can’t shot them they ran with their vans to find another plan, and they decided to remove them by pouring out the hot water and others were still in their sharks to get something to eat and the police took them out, you see, they wanted to kill everybody (M: hmm) yes, that was the problem.

**M:** So, Bro [name] you mentioned two things that anything that is silver was not allowed there and secondly, that they were not shootable, how did that happened, why those things were not allowed in the mountain?

**P:** In the mountain what happened was that, it was their traditional healer so he said as they were in the mountain, the money, phone and anything that is silver was not allowed, you see (M: okay), so this healer said, when you are going to the police, you must go without turning back even if you coming back you must not turn, you must just move with your backs and go back to where you staying and when you see an animal maybe a rabbit you must not kill it, even if you see a snake do not kill it, so now other Sothos after they were poured with hot water they saw a rabbit passing, they caught and killed it, that’s why people were killed and they wanted the meat (M: hmm), so they killed those animals and they were told not to kill any animal they see, they should have just left it.

**M:** Bro [name] as we are talking about people who were there, people who went to the mountain, I am trying to know if they wanted people who were brave, I am trying to think that there was already a threat that those people can hurt, so what kind of man did they wanted in the mountain?

**P:** As they were going there, they wanted every men who is a worker, you see (M: hmm), so they used that **muthi** when they arrived there, so everybody was told to come with his weapon, whatever you have be it a gun or **Umkhonto** so that they will use that **muthi** to protect from being harmed by any weapon and were told not to sleep with the woman, they must sleep there in the mountain, only woman who left in the shark, if a man slept with a woman he must not go there to the mountain. At first it happened, and the police were harmed/injured, then secondly, the police poured them with hot water and when they were running down, others saw this rabbit and they killed it, that’s why people were killed, people would have survived, and the police would have been injured more (M: oh).

**M:** So, Bro [name], like I said I have conducted these interviews last year (P: yes) I know that there was a conflict between the workers who were in the mountain and those who were going to work that time, so what was happening?

P: Let me say those who were in the mountain, like it was agreed that this is because they wanted money, but others were pleaded by the Boer**/Bhulu** people, they promised to pay them and if you have done a job you will get R500 today, others were injured they went to job because of that amount, then they went to attack the buses and beat people, it was the Amagundwana who went to work while others were not (M: okay), people were starving they ran to work to get the money, it was said that once you done with today’s work you get R500 same time, that’s why people ran there.

**M:** Tell me Bro [name] as you heard, like you were in jail that time, how were they dealing with Amagundwana?

**P:** They were attacked in their houses, they were beaten up, burnt and killed, and majority of them after this went successful, they resigned from work because they knew that they will be killed, they were left with a note in their rooms stating that “we know that you were working so we will come for you”, then they move and ran to the police stations, they resigned and went home to their children.

**M:** How do they knew that this one is a Gundwana?

**P:** The problem is, there was a clock card when you enter the gate, so they went to tape everything, you see, when you want the list of all those, because the clock card contains all your information, your name and time, so they can see that you reported at 3 and left at 10 you see, (M: okay), they went to the gate and tape everything and they came back with the list of [names] **27:13**, they went to all the sites of Lonmin, they knew that in that site we know who was working there, its so and so and these are their names, all of them, that is why they went to plug the note in their houses, saying that you sold us you didn’t want the money, we are busy striking but you allowing the Boere to use you, you working and when you knocking off you are paid R500 you solving your problems, so that’s why people were injured.

**M:** So, where did they found them?

**P:** Others were found in their houses, some were attacked from the buses, you see (M: hmm), they were targeting the bus and follow it to see where are they going and wait for them when they were coming back.

**M:** Bro [name], I’m interested in this thing because those people are colleagues, but they were separated because others were striking, and others were continue going to work, because now you are talking about the burning and killing, why those who killed others had the guts to kill someone they know, like they knew that this is you, but they ’ll kill you now.

**P:** They said you are a spy you see, you are on the Boers side, you cannot, while we are striking and you allowing them to tell you to go to work, that is why they did it because they hated that thing, it is hurting because they went to fetch another one from the site, they asked him, and told him that his wife is in the mountain, they killed him there, and he also revealed others when he saw that he was about to be killed, he had no choice he was told to write down the names of others who are also working, they were staying in different places, he wrote a long list then they killed him. Those people when they plan on doing something, they do, that **muthi** was very wrong, if they decided on killing you they’d not change their minds and forgive you, if you are in the car they would burn the car.

**M:** Exactly Bro [name] you are touching on what I’m saying, for an example, a man come from home to seek work and had a goal that I am working for my children, so the guts of killing a person where did it came?

**P:** I see that, it comes from that **muthi** they used there, you see that muthi it is exactly the reason to do evil things, if they were told to do this they do it (M: there’s no turning back?), there’s no turning back.

M: So, as you are explaining that, this one who was killed in the mountain was painfully killed, I am thinking maybe he was crying asking for forgiveness and all that.

**P:** He was crying, he was doing everything there, (M: hmm), they said when they call him and telling him that his wife is also in the mountain, ‘’we know your list and we’ve long sending you messages asking you to come so that we can talk’’, you were working right, then that’s ok we don’t have a problem but we want those people you are staying with in [name]**,** he named all of them then he died and they burnt him there.

**M:** How was he killed?

**P:** They slaughtered him using **Imikhonto,** all of them, and with that blood not knowing if they drank it or what they do with it (M: hmm), they burnt him there, then they came to [place] to look for others and others ran.

**M:** Others were working?

P: Yes, others were working, and others left, you see mostly are Amampondo, others said they will kill them back at home once someone ran, and others left to find other places (M: others said we all coming back from Eastern Cape?) yes, we will finish them there. When you had money, you would buy a house here in Gauteng and move with the family to live in Gauteng, you are trying to escape being killed at home with your family (M: hmm), those people could have come to place a bomb in the yard, and look for anyone who comes out of the house and they’d kill him/her, be it a mother or a child (M: hmm), that thing was very bad.

**M:** Those people who killed Amagundwana, how is their behaviour in the community, are they known that so and so was in the forefront of killing people, like how is this situation?

**P:** Yes, now you see it’s not good, but these people are known, all of them, like if I’m from maybe in [town] they would not reveal me, but hide me, and I would have to kill someone from another place if they heard that you are from maybe Bizana they’d send people from a different town to kill that person.

**M:** As we were far away from it, we watched it on the tv news and the newspapers, saying that others were undressed and humiliated.

**P:** Yes, that one was done in Mpala you see, (M: okay) and also in Lonmin, in Mpala they said when the Ngundwana goes to work they were undressed in their houses and forced them to have sexual intercourse with their wives, then after that, they kill them both, they were told to sleep together while others were watching and kill them because the husband is working you see, (M: hmm).

**M:** What were the reasons Bro [name] of forcing them to do this thing?

P: It’s like they wanted to take pictures of them and circulate it everywhere via WhatsApp then they killed them.

**M:** Their reason was to humiliate them or?

P: The way I saw, they said they were humiliating them and killed them after (M: After everything they have done?), after everything they have done, they didn’t leave without killing them.

**M:** Bro [name], you were just coming from home you ended up finding yourself in this situation, you got injured and you went to jail, how do you feel?

**P:** I feel painful, I just wish if I had just stayed at home and not coming back but I was interested because when people are striking, the workers, I thought I should be next to them without knowing that it will end up hurting me and got retrenched from work you see, I never went to use their **muthi** I didn’t know that, but I heard from people that this and that is not allowed there, then I went back to leave these things and I got injured (M: hmm). It’s very painful, more worse to a person who has done nothing, but they were also workers and I didn’t want to go back to work while others weren’t, so I decided to join others you see, (M: hmm), and when they didn’t see you in the mountain maybe 2-3days, they’d start checking you and think you are working.

**M:** Oh, when they don’t see you in the mountain for a couple of days, then they’d start checking you?

**P:** Yes, they think that you are working, like here we have houses and we have neighbors, if they don’t see you they ask to our neighbor, and find that you are working and they sent someone to check everything about you, if you are going to work at 3 then that person makes sure that he waits for you before that time, maybe at half past 2, or maybe he tries to talk to you by asking anything just to check where are going to and then he knows that you going to work, and he will report back the information that, around this time he goes to work, they plan to come to you maybe four of them, when you wake up trying to prepare, they wait next to your house and when you come out they take you to the mountain to make the list of others who are also working and after they kill you.

**M:** Did they left them there?

**P:** Yes, they left you in the mountain (M: jho), it is like that.

**M:** it is very bad what you are saying.

**P:** jho it is.

**M:** I would like to hear from you Bro [name] as you were here in Marikana and what you heard, those people who were in the mountain, did they go with their weapons to the police?

**P:** Yes, they carried their weapons, with **Umkhonto,** the first group was unshootable they injured the police, I am not sure how many police man did they killed, then secondly, it was them when they were poured by hot water and ran around the mountain, and others catch the rabbits and that is when they got injured and killed.

**M:** Bro [name] thank you very much, the way you are so free and you see me for the first time, the way you talked to me, thank you very much (P: I’m also thankful), like I said this information we want it for the research we are doing, it ends between the two of us, like I said the people I’m working with are my colleagues (P: Yes I know them). So, I’m trying to say Bro [name] thank you very much for your respect, it was your right to say no I’m busy and do not disturb me, but you gave me your time and spent your day, thank you very much. I don’t know if you have something to explain further, about what we were talking about, before we close.

P: No, it’s alright, there’s nothing more. Thank you.

M: Thank you very much Bro [name], have a good day.
