# Supplementary material for: “You see this thing is hard… ey, this thing is painful”: The burden of the provider role and construction of masculinities amongst Black male mineworkers in Marikana, South Africa
Source: PLoS One. 2022 May 23;17(5):e0268227. doi: 10.1371/journal.pone.0268227 (PMC9126392; doi:10.1371/journal.pone.0268227)
Supplement: Attachment — Submitted filename: 2021 - PLOS ONE-African men and migration.docx [file pone.0268227.s003.docx]

Peer Review of PONE-D-20-24827 - “You see this thing is hard… ey, this thing is painful”: The burden of the provider role and construction of masculinities amongst Black male mineworkers in Marikana, South Africa”

The authors draw upon 13 qualitative interviews to describe the construction of masculinity among South Africa mine workers in the context of poverty, migration, and family. The authors identify five key themes, which are clearly defined and identified for the most part. The sample illuminates the perspectives of a severely marginalized group of men, and as such is of interest to scholars in many fields, including gender, poverty, labor, and migration.

*The abstract and introduction* are clearly and concisely written to provide a roadmap to the paper’s scope and findings, situating the discussion of masculinities in Connell’s landmark theoretical framework. The authors also offer a succinct and well-organized description of the empirical context of men migrating for work in an area where economic opportunities are few and masculinity is rigidly defined.

*Material and Methods* – the author state that they interviewed 13 men, and “almost all” had families far away, and “almost all” had participated in the strike. If the point of the study is to examine how men felt about their long-distance provider roles, then all men in the sample would have to be separated from their families. I’d suggest that the sample is some number less than 13 then. Furthermore, unless the authors do more to explain the conceptual importance of the strike, this criterion is less relevant to the sample.

The description of the miners’ strike is compelling, but they do not really explain why the strike is important to understanding masculinities among their sample. How is the backdrop of a historical mineworkers’ strike connected to the study? Why did respondents need to have resided in the area for at least five years? What is it about firsthand experience with the strike and long-term migration patterns of 5+ years that would have made the manner in which these men understood and constructed masculinity different from men who had not been in the strike or had been migrating for less than five years?

Pg. 11 – this paper is about the intersections of marginalized masculinity, occupation, and health vulnerabilities. As such, the discussion of health vulnerabilities of mine workers needs to be expanded.

Theoretical framework – the concept of “hegemony in marginality” is particularly interesting as a way to understand how these men understood their roles and their status vis-à-vis women and other men. This should be discussed in far greater detail to enhance the overall contribution of the paper.

Analytical strategy – there is little description of the actual methods employed (lines 202-206). This portion of the paper needs to be expanded and better explained.

Line 219 – the authors state that they have used pseudonyms for the two locations interviews took place – is this accurate? If so, why was this necessary?

*Findings*

Distant fatherhood - The authors state that men felt very detached from their children, but do not offer much in the way of discussion or quotes in this section to make this point. This section should be revised accordingly (lines 470-474).

Line 276 – this point about patriarchy is one of the key findings of the paper, and this point should be drawn out to greater effect, as should all theoretical considerations of patriarchy and challenges to it throughout the paper.

Like 282 – I would argue this IS hyper-masculinity, not akin to it.

Lines 306-307 this is an excellent articulation of the one of the key themes in the paper, and something that should be addressed directly in the discussion and conclusion as well.

Line 310 – hypermasculinity was a strategy for gaining prestige among other men. Was it uniformly met with disapproval, as the authors write, or was it a matter of degree? In other words, were these behaviors tolerated, even admired, as long as the men in question sent money home?

Line 327 and line 426 – this is fascinating! That men took pride in performing traditionally feminine tasks because it allowed them to avoid other behaviors associated with the ‘wrong’ kind of masculinity (financially supporting a woman to care for/have sex with a man and neglecting his family back home). The temporal aspect of this is very interesting – I assume these men did NOT perform these tasks when they were home with their wives.

Line 402 – there needs to be a summation at the end of this section detailing the overall significance of the findings and tying it in to the rest of the paper.

Line 536-539 – the discussion of men’s willingness to see women become providers, and even be provided for by women is a significant challenge to patriarchal norms, a key theme in the paper. Draw this out in more detail. Check to make sure the titles of the subheadings for the five themes actually match the content of each section – some of them are not quite precise enough.

The term mistress is quite sexist. Is this what the men called their extramarital partners? If so, the authors should make this clear and only use the term when directly quoting the respondents. Otherwise, a more neutral terms like extramarital partner should be used.

*Discussion and Conclusion* – these two sections of the paper are a bit jumbled. I think you could smooth it out by following the organization of the previous parts of the paper to speak to your final conclusions and the implications of your research more systematically. The authors introduce new material and citations that are not clearly connected to the literature review or study. I suggest removing this part of the discussion and focus instead on findings and implications that have been demonstrated by the paper itself.

Again, without additional build up and discussion, I think referencing these constructions of masculinity as gender-transformative is going too far– it is more of an uneven challenge to masculinity. This is still quite important, as this is how patriarchy is broken down a piece at a time, but some more nuanced phrasing will help better frame the contribution of the paper in this area.
