# Supplementary material for: “You see this thing is hard… ey, this thing is painful”: The burden of the provider role and construction of masculinities amongst Black male mineworkers in Marikana, South Africa
Source: PLoS One. 2022 May 23;17(5):e0268227. doi: 10.1371/journal.pone.0268227 (PMC9126392; doi:10.1371/journal.pone.0268227)
Supplement: Attachment — Submitted filename: Response letter_30_06_2021.docx [file pone.0268227.s004.docx]

Dear Prof Kathleen Ragsdale

A sincere thank you to you for giving us the opportunity to have our paper entitled “You see this thing is hard… ey, this thing is painful”: The burden of the provider role and construction of masculinities amongst Black male mineworkers in Marikana, South Africa’ revised and resubmitted to PloS One.

We considered the reviewers’ comments and feedback to be very valuable and have summarised the amendments in the table below.

All the reviewers’ comments are listed and responses from the authors are given accordingly.

| **Editor** | **Response from authors** |
| --- | --- |
|  |  |
| We note that you have provided funding information that is not currently declared in your Funding Statement. However, funding information should not appear in the Acknowledgments section or other areas of your manuscript. We will only publish funding information present in the Funding Statement section of the online submission form. | Thanks to the Academic editor for pointing this out. We have now removed the funding information in in the manuscript. |
| Please include your amended statements within your cover letter; we will change the online submission form on your behalf. | The amended funding statement has been included in the cover letter. Thank you. |
|  |  |
| **Reviewer 1** | **Response from authors** |
|  |  |
| The present manuscript addresses a very interesting area of inquiry into masculinities among Black male mineworkers in Marikana, South Africa. The current version of the manuscript, however, needs to be improved. | We thank the review for this comment. Having addressed the comments from both reviewers, it is our view that the manuscript has been greately improved and is much stronger. |
| This manuscript is well structured and is analytical with comparative analysis conducted. | Thank you. |
| The manuscript needs improvement to ensure better reflection and synthesis. | With the revisions we have made, we think that the manuscript has been improved in these aspects. |
| The manuscript needs to be reviewed by a copyeditor. | We have carefully read the manuscript to correct the language and other typographical errors. |
| The introduction is well written. However, there is a need for the authors to see if information/description on the 2012 protest and social unrest is to be included in this manuscript. If yes, then the findings and discussions should reflect the impact of the incident on the mineworkers. | We thank the reviewer for raising this comment. The reference to the 2012 strike is relevant to the analysis done in this paper. Literature from South Africa has shown that poor Black men often engage in strikes or social unrest as a way of improving their social, political, and economic conditions (Moodie, 1986; 2005).  Furthermore, we have revised our findings section and it now clearly demonstrates how the 2012 mine workers’ strike was linked to some of the themes presented in the findings section. Specifically, we have expanded the ‘Providing is an inescapable demand’ theme to include findings on the reasons for the 2012 strike and how men’s participation in the strike meant they had to go without adequate food and income for several weeks, with this significantly impacting on their ability to provide for their families. |
| Descriptions in the methods sections could be improved. In-depth interviews with a small sample of respondents would have resulted in findings which should be richer in content than what presented in the findings section. I believe this could be because the data for question number 5-7 (in the attached interview guide) is not presented in this manuscript. | We thank the reviewer for raising this point. However, questions 5-7 in the IDI guide focus are on the actual strike and our view is that only the parts about the reasons for the strike and its impact on men’s provision are relevant for this paper rather than the actual descriptions of what occurred during the strike. We have now incorporated the relevant data which describes men’s reasons for engaging in the strike (e.g. low pay, poor living and working conditions) and its impact on men’s ability to provide for their families during the protracted period of the strike. |
| The discussion section summarizes and reflects back the findings. The authors further supported their arguments with past studies. However, this section could benefit more if theories/concepts of masculinities (eg. by Connell or by theorists on African masculinity/gender) were explicitly used to explain (also by means of agreeing or refuting etc.) the patterns in their findings and that from the literature. Overall, the authors need to take a step back and improve their Results and Discussion section. | We appreciate this motivation to include deeper and critical reflection in the Discussion on prior theories/concepts of masculinities that can explain the pattern of findings observed in our research. We believe that we have now reflected more the theories//concepts of masculinities in relation to our findings. We also use the Discussion to indicate potential avenues for intervention, based on our findings and on prior research. |
| L154: Any differences observed between the respondents based on their age, relationship status, and/or ethnic group? Findings presented based on men’s intersecting identities would be interesting to see if there are any similarities differences in findings due to these intersectionality. | Thanks to the reviewer for this comment. To address this comment, we have now added the following sentences in the Limitations section:  ‘Due to similarity in marital status and ethnic profiles among participants (who were predominantly from Nguni tribes – Xhosa, Pondo, Mpondomise), the data did not allow us to explore differences related to intersecting identities (i.e., ethnic group, relationship status, migration history, socioeconomic status). Future studies are necessary to ensure inclusion of men from diverse tribes and regions to enable exploration of these intersecting identities among male mineworkers.’ |
| L175: Why use theoretical sampling technique? The technique is used for theory building in the grounded theory method. Kindly justify if this is the case. Otherwise, purposive sampling technique is fine. | Thanks to the reviewer for this suggestion. We have now revised the paper to reflect that purposive sampling technique was used in both phases of data collection. |
| L177: Since this is a sensitive topic, do you want to omit 'lay counselors'. I imagine there may not be many who are working with the mineworkers and they could be potentially identified? | We thank the reviewer for this suggestion. The sentence referring to lay counsellors has been deleted. |
| L180/187-188: Need findings and discussion to reflect this. | We now refer to aspects of the strike that relate to men’s provider roles and experiences. |
| L199: If codes/themes were derived from the interview guides, then consider not stating that you used an 'inductive' approach. | We concur with the reviewer that there were deductive elements to our analysis. However, we wish to point out that most codes and themes were not derived from the interview guides. As such, we have revised the sentence as follows: ‘We analysed data inductively employing a thematic analysis approach (40, 44), however, there were deductive elements to our analysis as we explored themes that were related to material provision and migration to the mines. |
| L202 – 205: Consider revising the statements - What you are doing here is analyzing the transcripts, assigning codes to data and phenomena to express it in the form of concepts. | We thank the reviewer for the suggestion to revise our description of the analysis approach. We have sustained the reviewer’s suggestion and revised the statements accordingly. |
| L207: Are these references of the 'similar published studies' or of publications on qualitative research which suggests to make such comparisons? If it’s the latter, consider making it explicit in that statement. | We have deleted the sentence referred to by the reviewer as we agree that it is not clear. |
| L222: Consider removing references from past studies in your Results sections. By having a rich description of the findings alone will enable you to compare the findings with past studies and provide an understanding using theories of masculinities. Also, consider only inserting one or two (preferably short) quotes which reflects each themes/sub-themes in the Results section. | This has been done. Thanks to the reviewer for these suggestions. |
| L268-274: Remove the question by the interviewer. An example for you to consider “Ey, you are not recognized [if you are not married, built a house and have a family], you know they do not……” | Thanks to the reviewer for this suggestion. It has been sustained, and a revision to the quote has been done accordingly. |
| L416-424: Please check the use of quotations and quotations within quotations - according to the format of PLOS One | This has been corrected throughout the findings section, in line with PLOS ONE’s format. |
| L564-568: Consider paraphrasing/summarizing the direct quote | We thank the reviewer for this suggestion. The quotation from Gibbs et al. has now been paraphrased. |
| L573: Why personal communication? Can this be replaced with any research? | We have now added references from published research. |
|  |  |
|  |  |
| **Reviewer 2** |  |
|  |  |
| The authors draw upon 13 qualitative interviews to describe the construction of masculinity among South Africa mine workers in the context of poverty, migration, and family. The authors identify five key themes, which are clearly defined and identified for the most part. The sample illuminates the perspectives of a severely marginalized group of men, and as such is of interest to scholars in many fields, including gender, poverty, labor, and migration. | Thank you for the positive feedback on our paper. |
| The *abstract and introduction* are clearly and concisely written to provide a roadmap to the paper’s scope and findings, situating the discussion of masculinities in Connell’s landmark theoretical framework. The authors also offer a succinct and well-organized description of the empirical context of men migrating for work in an area where economic opportunities are few and masculinity is rigidly defined. | Thank you. |
| *Material and Methods* – the author state that they interviewed 13 men, and “almost all” had families far away, and “almost all” had participated in the strike. If the point of the study is to examine how men felt about their long-distance provider roles, then all men in the sample would have to be separated from their families. I’d suggest that the sample is some number less than 13 then. | We were interested in men’s provider roles in general and not only among those that had migrated. However, having read our aim again, both in the abstract and the introduction, we recognise that the aim as was stated in the abstract may have given the impression that we were only interested in views and experiences about provider roles of men that had migrated to the mines. We have now revised the text in the abstract to make it relevant for the whole sample. We have also added the element of migration to the aim stated at the end of the introduction as it was missing. |
| Furthermore, unless the authors do more to explain the conceptual importance of the strike, this criterion is less relevant to the sample. | Reviewer 1 raised a similar point and we have now shown the conceptual importance of the 2012 strike to this paper, showing both how the reasons for the strike were linked to men’s inability to provide and how the strike, in turn, impacted on their ability to earn an income and provide for their families during the strike period. |
| The description of the miners’ strike is compelling, but they do not really explain why the strike is important to understanding masculinities among their sample. How is the backdrop of a historical mineworkers’ strike connected to the study? | The response above addresses this comment. A clearer connection between the 2012 mine workers’ strike and findings of this study is now provided in the Findings and Discussion sections. |
| Why did respondents need to have resided in the area for at least five years? What is it about firsthand experience with the strike and long-term migration patterns of 5+ years that would have made the manner in which these men understood and constructed masculinity different from men who had not been in the strike or had been migrating for less than five years? | We thank the reviewer for this comment, we have now provided the following explanation in the Limitations section:  ‘This criterion was essential to capture experiences of workers present during the 2012 mine strike and examine how the strike affected their livelihoods and their ability to provide for their families, which was an objective of the parent study for this paper. Thus, our findings are not generalizable to other African mine worker contexts.’  These data, drawn from the main aims of the study, are being analysed for a separate paper which focusses on [male] mineworker’s constructions of masculinities during the strike and their exposure to police/military violence and their own use of violence against police and other mineworkers during the strike period. |
| Pg. 11 – this paper is about the intersections of marginalized masculinity, occupation, and health vulnerabilities. As such, the discussion of health vulnerabilities of mine workers needs to be expanded. | We thank the reviewer for this suggestion. We have now discussed our findings on health vulnerabilities of mine workers. We have added the following sentences in the Discussion section:  ‘Research evidence from South Africa and elsewhere suggests that mine workers experience increased risk of developing respiratory and pulmonary diseases (66, 67). Nelson (68) reported evidence for the risk of silicosis in platinum mine workers and asbestos-related diseases in diamond mine workers. Our finding adds to this growing body of evidence, as we have shown that participants were retrenched by the mines or retired early due to diseases attributed to prolonged exposure to dust, minerals and toxic chemicals found in the mines.’ |
| Theoretical framework – the concept of “hegemony in marginality” is particularly interesting as a way to understand how these men understood their roles and their status vis-à-vis women and other men. This should be discussed in far greater detail to enhance the overall contribution of the paper. | Thanks to the reviewer for this suggestion, the concept of hegemony in marginality is now discussed in the conclusion section of the paper. |
| Analytical strategy – there is little description of the actual methods employed (lines 202-206). This portion of the paper needs to be expanded and better explained. | In addressing reviewer 1’s comment, which is somewhat similar to this comment, we have significantly revised the description of the data analysis approach for this paper. Please see revised text from line 199-208. |
| Line 219 – the authors state that they have used pseudonyms for the two locations interviews took place – is this accurate? If so, why was this necessary? | We thank the reviewer for pointing out this error. The names of the two locations in which we conducted the interviews have not been changed. This error has now been corrected in the paper. |
| Findings |  |
| Distant fatherhood - The authors state that men felt very detached from their children, but do not offer much in the way of discussion or quotes in this section to make this point. This section should be revised accordingly (lines 470-474). | We are grateful to the reviewer for this suggestion. We have now added an extract from line 433-435 which shows that these men were distant fathers whose fatherhood mainly centred on material provision. A discussion of this finding is presented from line 632-635. |
| Line 276 – this point about patriarchy is one of the key findings of the paper, and this point should be drawn out to greater effect, as should all theoretical considerations of patriarchy and challenges to it throughout the paper. | We thank the reviewer for this suggestion. However, in the process of revising the findings section, the sentence referring to patriarchy has been deleted. |
| Like 282 – I would argue this IS hyper-masculinity, not akin to it. | We agree with the reviewer and have now revised this sentence accordingly to be clearer that these men, through their practices, were enacting hypermasculinity. |
| Lines 306-307 this is an excellent articulation of the one of the key themes in the paper, and something that should be addressed directly in the discussion and conclusion as well. | We are grateful to the reviewer for this suggestion. We have addressed this some more from line 586-592 in the Discussion section. |
| Line 310 – hypermasculinity was a strategy for gaining prestige among other men. Was it uniformly met with disapproval, as the authors write, or was it a matter of degree? In other words, were these behaviors tolerated, even admired, as long as the men in question sent money home? | We thank the reviewer for raising this important point. Indeed, hypermasculinity was not uniformly met with disapproval by all men interviewed as we have shown that 1) some men interviewed did engage in transactional sexual relationships or had had extramarital partners in the mines, and 2) under the theme ‘constructions of masculinity straddling spaces’ we have stated that performance of behaviours like having sexual relationships with multiple women and spending money publicly were reported to gain some men admiration and recognition by some women and men in the mining environment. We have now revised the sentence to reflect that not all men disapproved of this behaviour. |
| Line 327 and line 426 – this is fascinating! That men took pride in performing traditionally feminine tasks because it allowed them to avoid other behaviors associated with the ‘wrong’ kind of masculinity (financially supporting a woman to care for/have sex with a man and neglecting his family back home). The temporal aspect of this is very interesting – I assume these men did NOT perform these tasks when they were home with their wives. | Thanks to the reviewer for this comment. Yes, the reviewer is correct, this behaviour or practice was temporal as men did not perform these roles or tasks when their wives visited them in the mines or when men visited their rural homes. |
| Line 402 – there needs to be a summation at the end of this section detailing the overall significance of the findings and tying it in to the rest of the paper. | Thanks to the reviewer for this suggestion. We have now provided a summation at the end of this section from line 431-436. |
| Line 536-539 – the discussion of men’s willingness to see women become providers, and even be provided for by women is a significant challenge to patriarchal norms, a key theme in the paper. Draw this out in more detail. | We thank the reviewer for this suggestion. We have drawn this out in more detail from line 566-569. |
| Check to make sure the titles of the subheadings for the five themes actually match the content of each section – some of them are not quite precise enough. | We are grateful to the reviewer for this suggestion. We have now revised some of our themes and reorganised them to match the content within them. |
| The term mistress is quite sexist. Is this what the men called their extramarital partners? If so, the authors should make this clear and only use the term when directly quoting the respondents. Otherwise, a more neutral terms like extramarital partner should be used. | The term mistress has been removed in the paper and replaced by ‘extramarital partner’. |
| Discussion and Conclusion – these two sections of the paper are a bit jumbled. I think you could smooth it out by following the organization of the previous parts of the paper to speak to your final conclusions and the implications of your research more systematically. The authors introduce new material and citations that are not clearly connected to the literature review or study. I suggest removing this part of the discussion and focus instead on findings and implications that have been demonstrated by the paper itself. | The discussion and conclusion sections have now been revised significantly to ensure that they are better organized and flow smoothly and in a coherent manner. Also, we have removed new findings in the discussion and conclusion section to ensure that we only discuss salient findings and draw implications therefore. |
| Again, without additional build up and discussion, I think referencing these constructions of masculinity as gender-transformative is going too far– it is more of an uneven challenge to masculinity. This is still quite important, as this is how patriarchy is broken down a piece at a time, but some more nuanced phrasing will help better frame the contribution of the paper in this area. | We agree with the reviewer that it is a big leap to see the constructions of masculinities by some men as gender-transformative. We have significantly revised the discussion and conclusion sections and in that process revised our argument that some men’s constructions of health promotion masculinities must be seen as an incremental challenge to hegemonic masculinities. |
|  |  |

We hope that this resubmission meets with the requirements of PloS One.

Kind regards,

Yandisa Sikweyiya
