# Supplementary material for: “You see this thing is hard… ey, this thing is painful”: The burden of the provider role and construction of masculinities amongst Black male mineworkers in Marikana, South Africa
Source: PLoS One. 2022 May 23;17(5):e0268227. doi: 10.1371/journal.pone.0268227 (PMC9126392; doi:10.1371/journal.pone.0268227)
Supplement: Attachment — Submitted filename: Response to Reviewers.docx [file pone.0268227.s005.docx]

February 24, 2022

The Editor,

PloS One

Dear Prof Vanessa Carels,

**Re: Response to reviewers’ comments on manuscript submission: “You see this thing is hard… ey, this thing is painful”: The burden of the provider role and construction of masculinities amongst Black male mineworkers in Marikana, South Africa.**

We thank the editor and reviewers for taking time to review our manuscript. Below, we provide our responses to the reviewers’ comments and detail how we have addressed each comment raised by the reviewers:

**Reviewer #1**: The authors have revised the manuscript and adequately addressed the comments raised in the previous round of review.

**Response**

*Thank you.*

**Reviewer #2:** The authors have returned a thorough, thoughtful review that fully addresses the reviewer‘s comments. In particular, the impact of the 2012 mine strike is thoroughly woven into the findings and selection of quotations. There is a more thorough overview of both empirical literature and conceptual frameworks, and theory is woven into the findings in a much more comprehensive matter. This in turn helps establish a clear contribution of the work.

**Response**

*Thank you.*

There are a few minor revisions that are still needed.

The authors should consistently use ‘mine work’ or ‘minework.’ The spelling varies throughout the paper.

**Response:**

*We thank the reviewer for pointing this out. We have now consistently used ‘minework’ throughout the manuscript.*

The word mistress appears on page 13. Please change it to non-marital partner or non-marital sexual partner, as the authors have done throughout the rest of the paper.

**Response:**

*The word mistress has been replaced with non-marital sexual partner throughout the manuscript.*

The order in which the authors indicate they will discuss the findings are not in the same order in which the findings were actually discussed. Specifically, the second and third themes are switched around.

**Response:**

*We thank the reviewer for pointing this out. We have now reorganized our discussion in the order in which we proposed to discuss them.*

The sections on provision as an escapable burden and hopes for alternatives for family are very short. In fact, they are really too short to fully make the authors’ points. In contrast, the discussion is four pages, The discussion should summarize the findings and explain the significance of the reader. Actual findings should appear above the discussion.

**Response:**

*We are grateful to the reviewer for this comment and suggestion. We have now expanded the sections on’ Provision as an inescapable demand’ and ‘Creating alternatives’. In both sections we now start by summarizing the key findings, discuss them in relation to relevant literature and then explain the significance of the findings and their implications for gender-transformative interventions.*

With these changes, I believe the paper will be close to ready for publication.

**Response**

*Thanks to the reviewer. We also hope that with the changes we have made in the manuscript, the manuscript will be found to be suitable for publication in PloS One.*
